# Rapid Access to Hydroxyfluoranthenes via a Domino Suzuki–Miyaura/Intramolecular Diels–Alder/Ring-Opening Reactions Sequence

**DOI:** 10.1021/acs.joc.1c03080

**Published:** 2022-04-07

**Authors:** Dilgam Ahmadli, Yesim Sahin, Eylul Calikyilmaz, Onur Şahin, Yunus E. Türkmen

**Affiliations:** † Department of Chemistry, Faculty of Science, Bilkent University, Ankara, 06800, Turkey; ‡ Department of Occupational Health & Safety, Faculty of Health Sciences, Sinop University, Sinop, 57000, Turkey; § Department of Chemistry, Faculty of Science, and UNAM, National Nanotechnology Research Center, Institute of Materials Science and Nanotechnology, Bilkent University, Ankara, 06800, Turkey

## Abstract

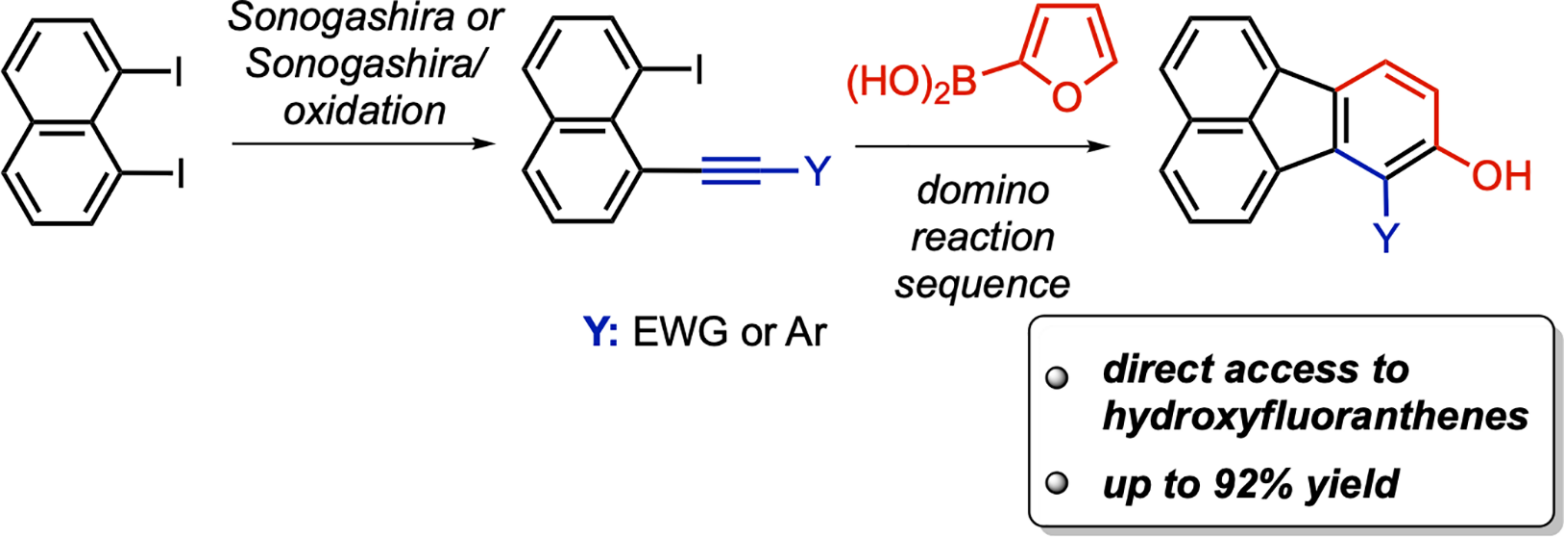

In this work, we developed an efficient method for the rapid construction of fluoranthene skeleton to access a variety of substituted hydroxyfluoranthenes. The 1-iodo-8-alkynylnaphthalene derivatives, which serve as substrates for the key fluoranthene-forming step, were prepared via selective monoalkynylative Sonogashira reactions of 1,8-diiodonaphthalene. The domino reaction sequence which involves a sequential Suzuki–Miyaura coupling, an intramolecular Diels–Alder reaction, and an aromatization-driven ring-opening isomerization has been shown to give substituted hydroxyfluoranthenes in up to 92% yield. This work demonstrates the utility of designing new domino reactions for rapid access to substituted polycyclic aromatic hydrocarbons (PAHs).

Polycyclic aromatic hydrocarbons (PAHs) represent an important class of organic molecules that attracted significant attention from the synthetic community due to their diverse applications.^1^ In particular, fluoranthenes constitute a widely encountered subclass of polycylic aromatic hydrocarbons, most members of which are fluorescent.^2^ The optoelectronic properties of substituted fluoranthenes have culminated in a broad range of applications including design of fluorescent probes,^3^ yellow and blue organic light emitting diodes (OLEDs, Figure 1, compounds **1** and **2**),^4,5^ thin film organic field-effect transistors (OFETs, compound **3**),^6^ and new materials for organic photovoltaic cells.^7^ Moreover, benzo[*j*]fluoranthenes comprise the structural skeleton of many highly oxygenated, biologically active fungal natural products including truncatone C (**4**)^8^ and daldinone B (**5**, Figure 1).^9,10^

**Figure 1:**
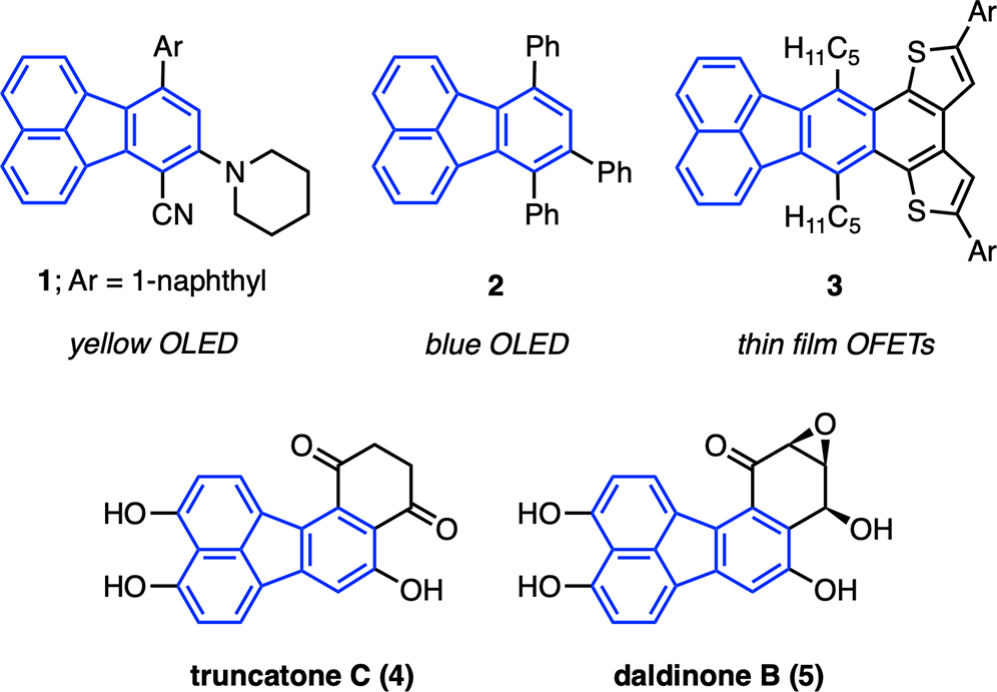
Important fluoranthene analogues.

Catalytic methods using transition metal complexes,^11^ Lewis acids,^12^ or Brønsted acids^13^ are among the most commonly applied transformations for the synthesis and derivatization of fluoranthenes. Cycloaddition and cyclization reactions have also been utilized for the construction of the fluoranthene skeleton from simpler building blocks.^3,4,14−16^ For instance, inverse electron-demand Diels–Alder reactions of cyclopentadienone **6** with alkynes at high temperatures (200–220 °C) were shown to provide multisubstituted fluoranthenes (**7**) in an effective manner (Scheme 1a).^5,17^ In a study reported by Lu, Wang and co-workers in 2011, the I_2_-mediated cyclization of dialkynylnaphthalenes (e.g., compound **8**) resulted in the formation of iodofluoranthenes (Scheme 1b).^18^ Compared to aryl-substituted fluoranthenes, direct access to hydroxyfluoranthenes has been rather underdeveloped. In a rare example of such a transformation, alkenyl ketone substrates **10** were reported to give complex hydroxyfluoranthenes **11** via an anionic-radical reaction cascade promoted by KHMDS (Scheme 1c).^19^ Against this background, with the goal of discovering a direct method to access hydroxyfluoranthenes without the requirement of protection/deprotection steps, we designed the reaction sequence shown in Scheme 1d. According to this design, 1-iodo-8-alkynylnaphthalenes (**12**), which were planned to be prepared by a selective monoalkynylation of 1,8-diiodonaphthalene, would be subjected to a Suzuki–Miyaura coupling with 2-furylboronic acid under Pd catalysis. In the coupling products **13**, furan and alkyne moieties are perfectly aligned in space for an intramolecular Diels–Alder reaction^20^ to give cycloadducts **14**, which were expected to undergo a ring opening reaction to release the ring strain and gain aromaticity to ultimately form hydroxyfluoranthenes **15**. Moreover, we hypothesized that these three steps could proceed under the same reaction conditions resulting in a domino reaction sequence,^21^ which would give directly the targeted hydroxyfluoranthene products. It is important to note that this hypothesis was based on the assumption that the reaction conditions for the initial Suzuki–Miyaura coupling would be suitable for the subsequent intramolecular Diels–Alder and ring-opening reactions.

**Scheme 1:**
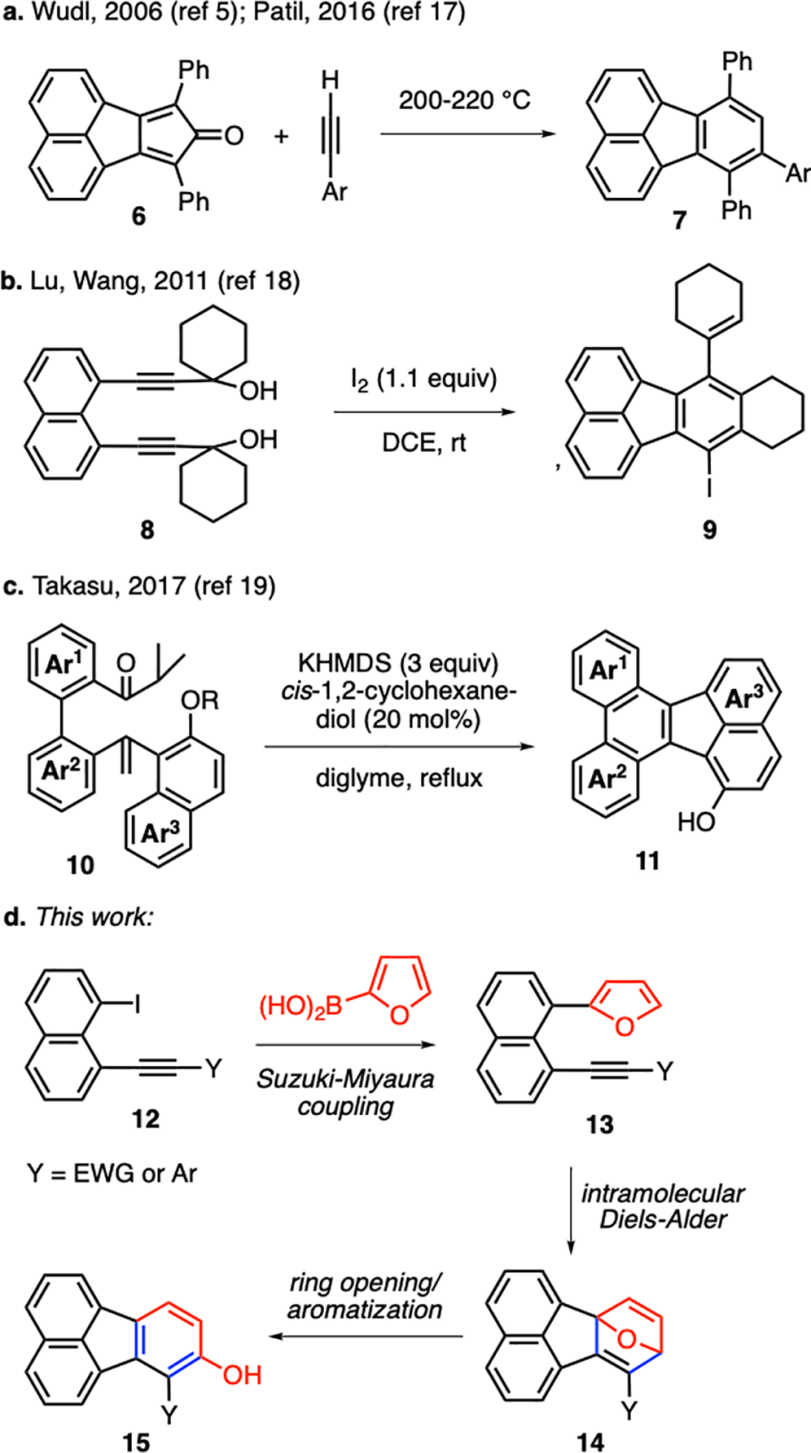
Synthesis of Fluoranthenes by Cycloaddition and Cyclization Reactions

In order to test our hypothesis, we first prepared alkynone **12a** to be used in the designed domino reaction sequence (Scheme 2). For this purpose, 1,8-diiodonaphthalene (**16**) was subjected to a Sonogashira cross-coupling with propargylic alcohol **17a** to afford the monoalkynylation product **18a** in 71% yield. It should be noted that, in the Sonogashira reactions carried out in the present work, the use of an excess amount of 1,8-diiodonaphthalene (4 equiv) was found to be crucial to minimize the formation of dialkynylation products. Pleasingly, an unreacted excess of 1,8-diiodonaphthalene (**16**) was isolated with 75% recovery at the end of its Sonogashira reaction with alkyne **17a**. Oxidation of alcohol **18a** proceeded efficiently with MnO_2_ giving the ketone product **12a** in 91% yield. In the key domino reaction step, we were delighted to obtain hydroxyfluoranthene product **15a** in an excellent yield of 92% upon the reaction of alkynone **12a** with 2-furylboronic acid (**19**) in the presence of Pd(PPh_3_)_4_ (Scheme 2). We reckon that the initial Suzuki–Miyaura cross-coupling would form **13a**, which would be both electronically and geometrically well-suited for an intramolecular Diels–Alder reaction to give pentacyclic intermediate **14a**. Finally, the spontaneous aromatization-driven ring-opening isomerization of **14a** would result in the formation of hydroxyfluoranthene **15a**. This three-step sequence takes place under the same reaction conditions in a domino fashion obviating the need for the isolation of intermediates **13a** and **14a**.

**Scheme 2:**
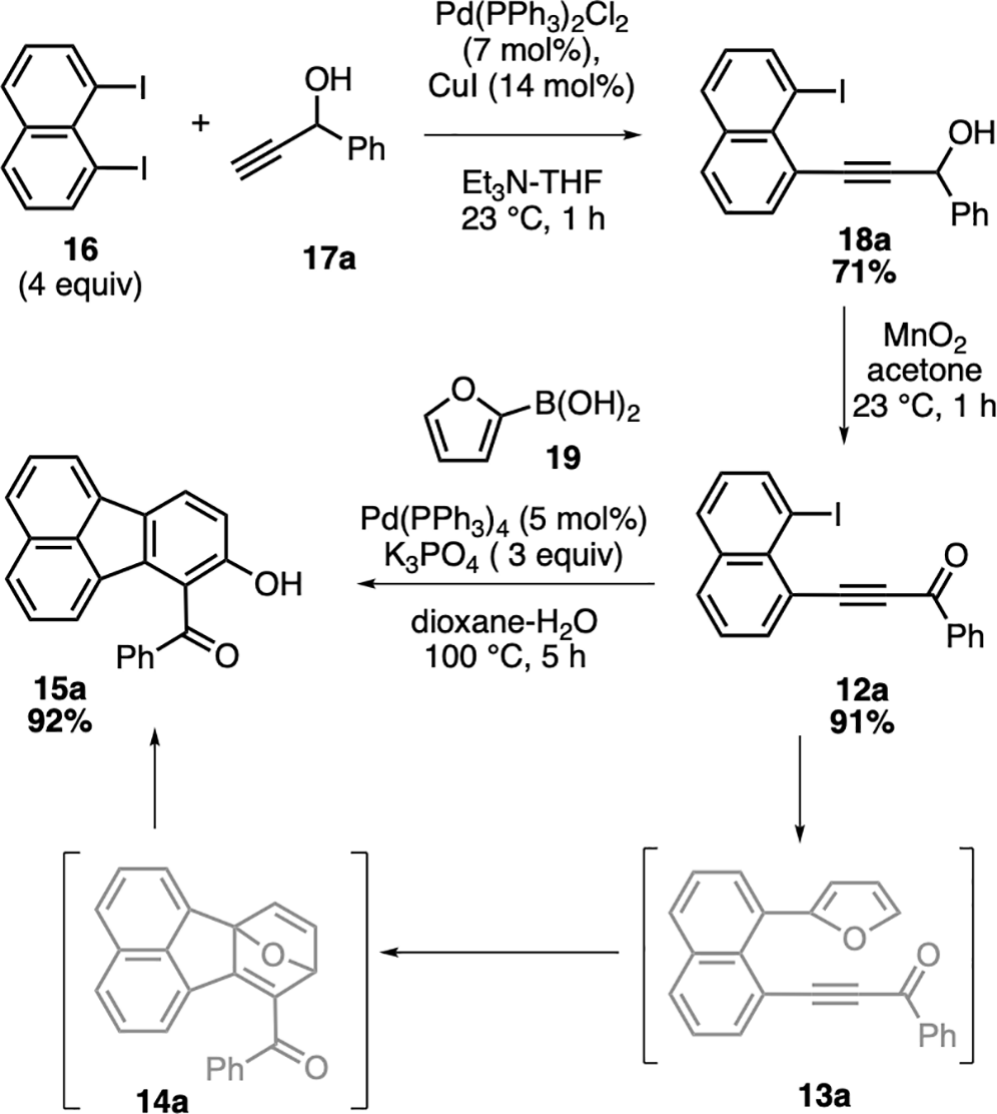
Synthesis of Hydroxyfluoranthene **15a**

With the validation of our hypothesis on the designed domino reaction sequence for fluoranthene synthesis, we next decided to investigate the scope of this methodology. To this end, we first synthesized 1-iodo-8-alkynylnaphthalene derivatives **12** which would serve as the precursors for the key domino reaction (Table 1). The aryl-substituted alkynones **12b**-**g** were prepared by an efficient two-step sequence. Initially, the highly selective monoalkynylative Sonogashira coupling reactions between 1,8-diiodonaphthalene (**16**) and propargylic alcohols **17** afforded monoiodoalkynol products in 70–85% isolated yields along with minimal formation of dialkynylation products (Table 1, entries 1–6). It is worth noting that this optimized protocol works successfully with both electron-rich and electron-deficient aryl groups as well as heteroaromatic substituents such as thiophene and furan. Among the methods tested for the oxidation of alkynols **18** to alkynones **12**, PCC (pyridinium chlorochromate)^22^ and Parikh-Doering oxidation^23^ methods worked with moderate reaction yields.^24^ On the other hand, both DMP (Dess-Martin periodinane)^25^ and MnO_2_ were found to be highly effective oxidants for this transformation with MnO_2_ providing slightly higher yields. Overall, the oxidation of alkynols **18b**-**g** to alkynones **12b**-**g** were achieved in 53–90% yields (Table 1, entries 1–6). Finally, Me-substituted alkynone **12h** and alkynyl amide **12i** were synthesized in 53 and 82% yields, respectively, via the direct monoalkynylative Sonogashira coupling of **16** with the commercially available 3-butyn-2-one (**20a**) and propiolamide (**20b**) (Table 1, entries 7 and 8). It is important to note that electron-deficient Me-substituted alkyne **20a** was observed to undergo decomposition when Et_3_N was used both as base and the reaction solvent in the Sonogashira coupling, possibly via an aza-Michael-type reaction pathway. This decomposition was circumvented with the use of the bulkier and less nucleophilic Hünig’s base (*i*-Pr_2_NEt) in the Sonogashira coupling along with DMSO as solvent.^26^

**Table 1:**
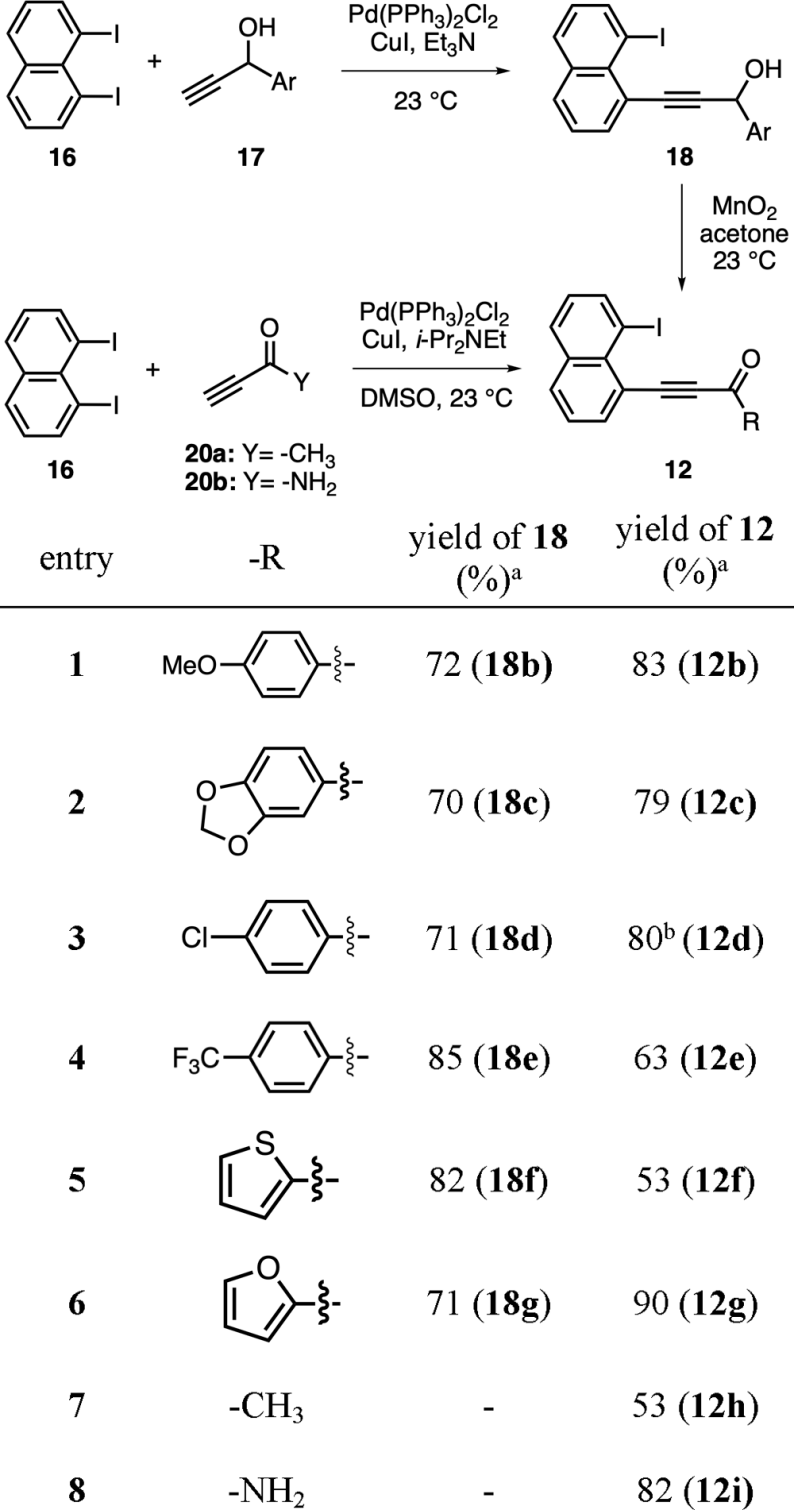
Synthesis of Alkynones **12** as Precursors of the Domino Reaction Sequence for Fluoranthene Synthesis

a Yields refer to isolated product yields after purification by column chromatography.

b In this reaction, Dess-Martin periodinane (DMP) was used as the oxidant.

With the alkynes **12b**–**i** in hand, we next focused on their reactivity in the key domino reaction sequence for the synthesis of targeted hydroxyfluoranthenes. When alkynones **12b** and **12c** bearing electron-rich phenyl rings were reacted with 2-furylboronic acid (**19**) under the coupling conditions, hydroxyfluoranthenes **15b** and **15c** were isolated in 73% and 90% yields, respectively (Scheme 3). The domino reaction was observed to work successfully with electron-deficient aryl rings as well affording fluoranthene products **15d** and **15e** in good yields (69% and 52%, respectively). Afterward, we turned our attention to heteroaromatic alkynone substrates. We were pleased to see that the domino reaction of thienyl-substituted alkynone **12f** gave the corresponding hydroxyfluoranthene **15f** in excellent yield (91%). Gratifyingly, the furan ring present in **12g** did not interfere in its reaction with 2-furylboronic acid (**19**), and the desired product **15g** was isolated in 72% yield. The methyl-substituted alkynone **12h** was also found to be a competent substrate in the domino sequence affording fluoranthene **15h**, albeit in a lower yield (38%). Finally, the domino reaction sequence of the amide-containing substrate **12i** led successfully to the formation of the desired product **15i** in 53% isolated yield.

**Scheme 3:**
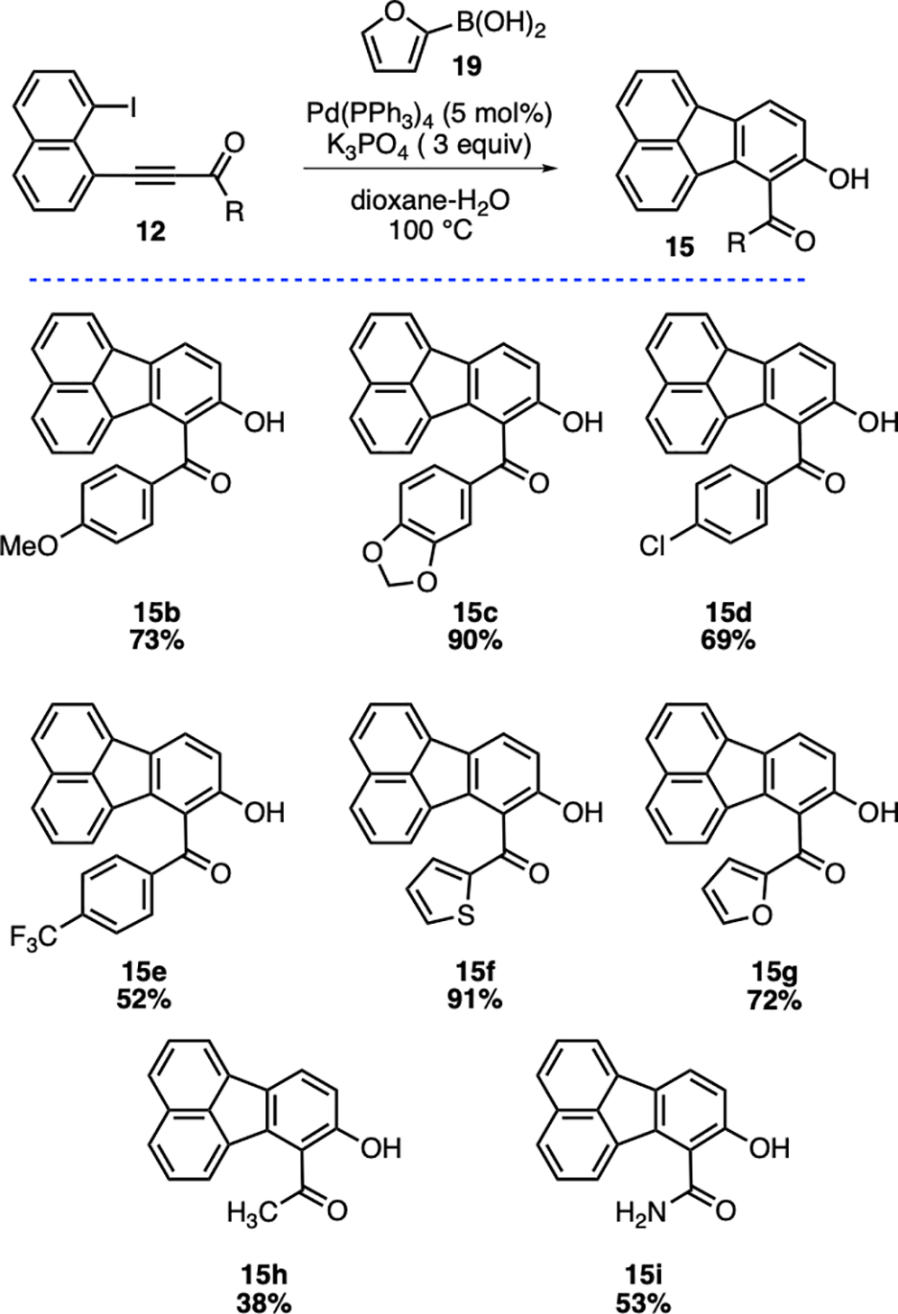
Scope of the Fluoranthene Synthesis

The phenolic −OH groups of the fluoranthene products **15a**–**i** were all observed to make intramolecular hydrogen bonds with their neighboring ketone or amide carbonyls as revealed by the corresponding signals between 7.99 and 10.53 ppm in their ^1^H NMR spectra.^27^ In addition, the structure of amide-substituted fluoranthene product **15i** was confirmed by single-crystal X-ray diffraction analysis (Figure 2a). The intramolecular hydrogen bond between the phenol −OH and the amide C=O is clearly observed in this structure with an O–H**···**O=C distance of 1.84 Å. Not surprisingly, the −CONH_2_ group is tilted with a dihedral angle of 35.6° with respect to the plane of the phenol ring, possibly to minimize the steric repulsion between the naphthalene C–H and amide −NH_2_ hydrogens. Interestingly, in addition to the intramolecular hydrogen bond with the −OH group, the amide oxygen participates in two additional intermolecular hydrogen bonds with the −NH hydrogens of two adjacent fluoranthenes with N–H**···**O=C distances of 2.10 and 2.21 Å (Figure 2b).

**Figure 2:**
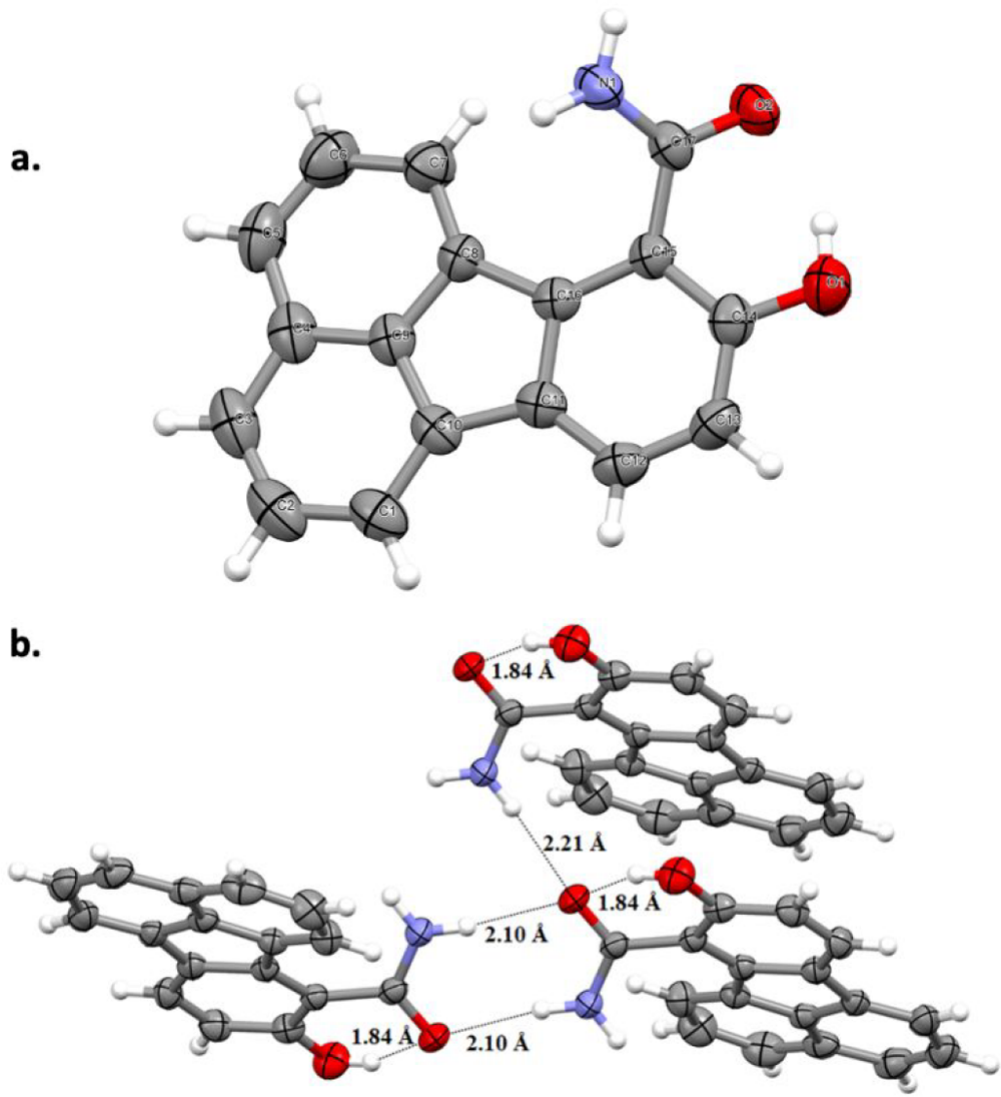
Crystal structure of fluoranthene **15i** (a) showing 50% probability displacement ellipsoids and the atomic numbering; (b) showing the formation of O–H···O and N–H···O hydrogen bonds.

Finally, we sought to test the effectiveness of the intramolecular Diels–Alder reaction of alkynylnaphthalene substrates without electron-withdrawing groups. To this end, we first prepared aryl-substituted alkynes **12j** and **12k** to be used in the domino sequence (Scheme 4). The monoalkynylative Sonogashira coupling between **16** and phenylacetylene proceeded smoothly under the optimized conditions to afford iodoalkyne **12j** in 84% yield. When **12j** was subjected to the standard domino sequence conditions, Suzuki–Miyaura coupling product **13j** was obtained as the major product in 92% yield (Scheme 4). This result is not surprising when the electron-rich nature of furan as a diene and the Ph-substituted alkyne as a dienophile in the structure of **13j** is considered. However, when a pure sample of **13j** was heated in mesitylene at 130 °C, fluoranthene **15j** was observed to form in 55% yield. This observation clearly supports the intermediacy of 1-furyl-8-alkynylnaphthalenes en route to the formation of fluoranthenes in the developed domino reaction sequence. It should also be noted that the reaction between **12j** and **19** with the use of Pd(PPh_3_)_4_ at 80 °C in EtOH/water gave Suzuki–Miyaura product **13j** in 53% yield along with the fluoranthene product **15j** (14% yield) with a reaction time of 50 h. Next, in order to see the effect of an electron-withdrawing group on the aryl ring, 4-nitrophenyl-substituted iodoalkyne **12k** was prepared in 86% yield via the Sonogashira reaction of **16** with 1-ethynyl-4-nitrobenzene (**21**). The reaction of **12k** with 2-furylboronic acid (**19**) gave hydroxyfluoranthene **15k** in 25% yield, indicating a slight benefit of having an electron-withdrawing −NO_2_ group on the aryl ring in the intramolecular Diels–Alder reaction with furan.

**Scheme 4:**
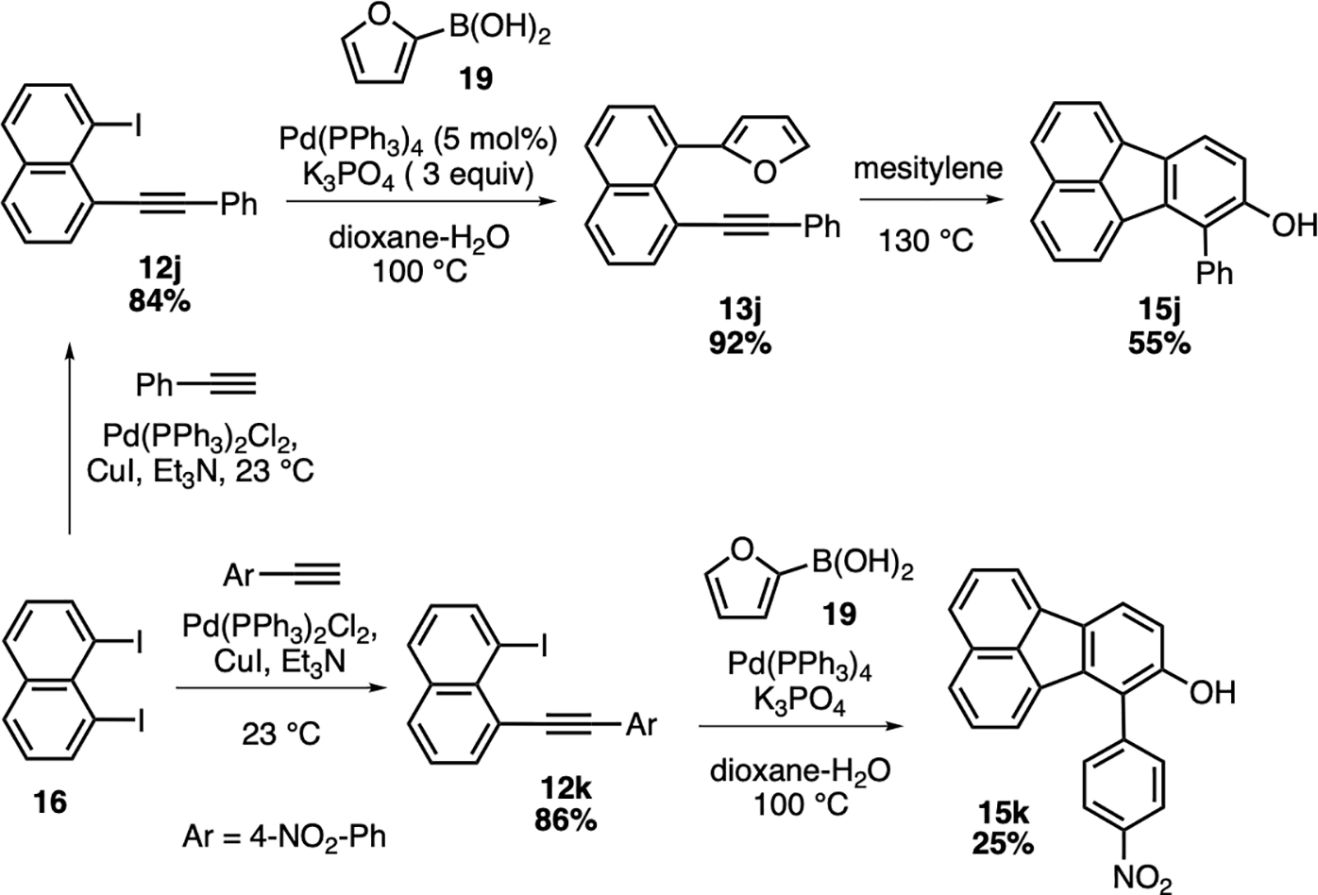
Intramolecular Diels–Alder Reactions of Aryl-Substituted Alkynes

In summary, we have developed a new strategy to rapidly access substituted hydroxyfluoranthenes via a carefully designed domino reaction sequence. The first step of the newly developed method involves a selective monoalkynylative Sonogashira cross-coupling of 1,8-diiodonaphthalene that leads to the formation of a variety of 1-iodo-8-alkynylnaphthalenes. The propargylic alcohol derivatives **18a**–**g** were oxidized to the corresponding ketones in high yields (53–91%). The key domino reaction sequence starting from iodoalkynes **12** and 2-furylboronic acid with the use of Pd(PPh_3_)_4_ (5 mol %) afforded hydroxyfluoranthene products **15** effectively in up to 92% yield. This domino sequence consists of a Suzuki–Miyaura coupling, an intramolecular Diels–Alder reaction, and an aromatization-driven ring-opening isomerization, which all occur under the same reaction conditions. It is important to note that this method obviates the need to have protection/deprotection steps on the −OH group, as it provides directly the hydroxyfluoranthene products at the end of the domino sequence. Studies to discover novel domino reactions to enable access to other types of polycyclic aromatic hydrocarbons are currently underway in our laboratory.

## Experimental Section

### General Information

All reactions except the oxidation reactions of alcohols **18** with MnO_2_ were performed using oven-dried glassware under an inert atmosphere of nitrogen. Aluminum-backed plates precoated with silica gel (Silicycle, 60 Å, F_254_) were used for reaction monitoring by thin-layer chromatography (TLC). UV light (254 and 366 nm) and KMnO_4_ staining solution were used for TLC visualization. Flash column chromatography was carried out using Silicycle 40–63 μm (200–400 mesh) flash silica gel. NMR spectra were recorded on a Bruker spectrometer at 400 MHz for ^1^H NMR spectra and 100 MHz for ^13^C{^1^H} spectra, and calibrated from an internal standard (TMS, 0 ppm) or residual solvent signals (chloroform at 7.26 ppm for ^1^H NMR spectra; chloroform at 77.16 ppm for ^13^C NMR spectra). For ^19^F{^1^H}-NMR experiments, trifluoroacetic acid (CF_3_CO_2_H) was used as external reference (−76.55 ppm). ^1^H NMR data are reported as follows: chemical shift (parts per million, ppm), integration, multiplicity (s = singlet, d = doublet, t = triplet, dd = doublet of doublets, m = multiplet, br = broad, app = apparent), coupling constant (Hz). Infrared (FTIR) spectra were recorded using a Bruker Alpha-Platinum-ATR spectrometer, and only selected peaks are reported. HRMS (high resolution mass spectrometry) analyses were performed on Agilent Technologies 6224 TOF LC/MS at the UNAM-National Nanotechnology Research Center and Institute of Materials Science and Nanotechnology, Bilkent University, and on Agilent Technologies 6530 QTOF-LC/MS at DAYTAM-East Anatolia High Technology Application and Research Center, Atatürk University. Single-crystal XRD analysis was performed at the Scientific and Technological Research Application and Research Center, Sinop University, Turkey. Melting points are uncorrected. Anhydrous CH_2_Cl_2_, THF, and 1,4-dioxane were purchased from Acros Organics (AcroSeal). 1,8-Diaminonaphthalene was recrystallized from *n*-heptane prior to use. 2-Furylboronic (furan-2-boronic) acid was purchased from Acros Organics and used as received. Furan-2-boronic acid pinacol ester was prepared following a reported procedure.^28^ Unless stated otherwise, all commercially available reagents were used without further purification.

#### General Procedure A for the Sonogashira Reaction Between Alkynes and 1,8-Diiodonaphthalene (**16**)

To a solution of alkyne (1.0 equiv) and 1,8-diiodonaphthalene (**16**, 4 equiv) in Et_3_N or THF/Et_3_N, Pd(PPh_3_)_2_Cl_2_ (7 mol %) and CuI (14 mol %) were added at 23 °C under N_2_. The resulting reaction mixture was stirred at 23 °C until TLC showed full consumption of the alkyne. Usually, a color change from yellow to orange was observed. Et_3_N was removed under reduced pressure. The remaining residue was dissolved in a sufficient amount of EtOAc or CH_2_Cl_2_ and washed once with H_2_O. The organic phase was dried over anhydrous Na_2_SO_4_, filtered, and concentrated under reduced pressure. The residue was purified by flash column chromatography. ***Note:*** The use of 2 equiv of 1,8-diiodonaphthalene was observed to afford the desired monoalkynylation product in considerably lower yield.

#### 3-(8-Iodonaphthalen-1-yl)-1-phenylprop-2-yn-1-ol (**18a**)

Product **18a** was prepared using 1,8-diiodonaphthalene (**16**, 1.15 g, 3.02 mmol), alkyne **17a** (100 mg, 0.76 mmol), Pd(PPh_3_)_2_Cl_2_ (37.2 mg, 0.053 mmol), CuI (20.3 mg, 0.11 mmol), Et_3_N (6 mL) and THF (2 mL) according to General Procedure A. The crude product was purified by flash column chromatography (SiO_2_; EtOAc/hexanes = 1:5) to afford pure **18a** (208 mg, 71% yield) as an orange oil. After column chromatography unreacted 1,8-diiodonaphthalene (**16**, 858 mg, 75%) was recovered. *R*_*f*_ = 0.31 (EtOAc/hexanes = 1:5). ^1^H NMR (400 MHz; CDCl_3_) δ: 8.26 (1H, dd, *J* = 7.4, 1.3 Hz), 7.86 (1H, dd, *J* = 7.2, 1.4 Hz), 7.82 (1H, dd, *J* = 4.3, 1.2 Hz), 7.80 (1H, dd, *J* = 4.5, 1.3 Hz), 7.70–7.66 (2H, m), 7.45–7.33 (4H, m), 7.09 (1H, dd, *J* = 8.0, 7.5 Hz), 5.81 (1H, d, *J* = 5.3 Hz), 2.46 (1H, d, *J* = 5.8 Hz). ^13^C{^1^H} NMR (100 MHz; CDCl_3_) δ: 142.9, 140.2, 136.5, 135.0, 132.1, 130.9, 130.3, 128.8, 128.5, 127.3, 127.2, 125.5, 122.0, 100.0, 92.9, 86.4, 66.1. FTIR ν_max_ (ATR, film)/cm^–1^ 3371, 2918, 2850, 1553, 1493, 1453, 1362, 1196, 1047, 1036, 1002, 947, 817, 758, 717, 698. HRMS (ESI+) calcd for C_19_H_13_INaO [M + Na]^+^, 406.9903; found, 406.9896.

#### 3-(8-Iodonaphthalen-1-yl)-1-(4-methoxyphenyl)prop-2-yn-1-ol (**18b**)

Product **18b** was prepared using 1,8-diiodonaphthalene (**16**, 291 mg, 0.76 mmol), alkyne **17b** (31 mg, 0.19 mmol), Pd(PPh_3_)_2_Cl_2_ (9.4 mg, 0.013 mmol), CuI (5.1 mg, 0.026 mmol), and Et_3_N (6 mL) according to General Procedure A. The crude product was purified by flash column chromatography (SiO_2_; EtOAc/hexanes = 1:9 → 1:5) to afford pure **18b** (57 mg, 72% yield) as a reddish orange oil. After column chromatography unreacted 1,8-diiodonaphthalene (**16**, 209 mg, 72%) was recovered. *R*_*f*_ = 0.40 (EtOAc/hexanes = 1:3). ^1^H NMR (400 MHz; CDCl_3_) δ: 8.24 (1H, dd, *J* = 7.4, 1.2 Hz), 7.84 (1H, dd, *J* = 7.3, 1.4 Hz), 7.82–7.75 (2H, m), 7.60 (2H, app d, *J* = 8.6 Hz), 7.38 (1H, dd, *J* = 8.2, 7.3 Hz), 7.07 (1H, dd, *J* = 8.0, 7.5 Hz), 6.93 (2H, d, *J* = 8.8 Hz), 5.76 (1H, s), 3.81 (3H, s), 2.65 (1H, br s). ^13^C{^1^H} NMR (100 MHz; CDCl_3_) δ: 159.8, 142.8, 136.4, 134.9, 132.6, 130.8, 130.2, 128.6, 128.4, 127.2, 125.4, 122.1, 114.1, 100.3, 92.9, 86.04, 65.6, 55.5. FTIR ν_max_ (ATR, film)/cm^–1^ 3399 (br), 1610, 1510, 1362, 1303, 1248, 1172, 1034. HRMS (ESI+) calcd for C_20_H_15_IO_2_ [M]^+^, 414.0111; found, 414.0116.

#### 1-(Benzo[*d*][1,3]dioxol-5-yl)-3-(8-iodonaphthalen-1-yl)prop-2-yn-1-ol (**18c**)

Product **18c** was prepared using 1,8-diiodonaphthalene (**16**, 345 mg, 0.92 mmol), alkyne **17c** (40 mg, 0.23 mmol), Pd(PPh_3_)_2_Cl_2_ (11.2 mg, 0.016 mmol), CuI (6.05 mg, 0.032 mmol), and Et_3_N (6 mL) according to General Procedure A. The crude product was purified by flash column chromatography (SiO_2_; EtOAc/hexanes = 1:4 → 1:2) to afford pure **18c** (68 mg, 70% yield) as a reddish yellow oil. After column chromatography, unreacted 1,8-diiodonaphthalene (**16**, 275 mg, 80%) was recovered. *R*_*f*_ = 0.48 (EtOAc/hexanes = 1:2). ^1^H NMR (400 MHz; CDCl_3_) δ: 8.25 (1H, dd, *J* = 7.4, 1.3 Hz), 7.84 (1H, dd, *J* = 7.2, 1.4 Hz), 7.81–7.76 (2H, m), 7.38 (1H, dd, *J* = 8.2, 7.3 Hz), 7.19 (1H, d, *J* = 1.7 Hz), 7.13 (1H, dd, *J* = 8.0, 1.7 Hz), 7.07 (1H, dd, *J* = 8.0, 7.5 Hz), 6.82 (1H, d, *J* = 8.0 Hz), 5.97 (2H, s), 5.70 (1H, s). 2.63 (1H, s). ^13^C{^1^H} NMR (100 MHz; CDCl_3_) δ: 148.0, 147.7, 142.8, 136.4, 134.9, 134.4, 132.0, 130.9, 130.3, 127.2, 125.5, 121.9, 120.8, 108.3, 107.9, 101.3, 100.0, 92.9, 86.2, 65.9. FTIR ν_max_ (ATR, film)/cm^–1^ 3396, 2961, 2922, 1553, 1501, 1486, 1442, 1362, 1246, 1093, 1038, 936. HRMS (ESI+) calcd for C_20_H_13_INaO_3_ [M + Na]^+^, 450.9802; found, 450.9796.

#### 1-(4-Chlorophenyl)-3-(8-iodonaphthalen-1-yl)prop-2-yn-1-ol (**18d**)

Product **18d** was prepared using 1,8-diiodonaphthalene (**16**, 273 mg, 0.72 mmol), alkyne **17d** (30 mg, 0.18 mmol), Pd(PPh_3_)_2_Cl_2_ (8.8 mg, 0.013 mmol), CuI (5.0 mg, 0.026 mmol), and Et_3_N (5 mL) according to General Procedure A. The crude product was purified by flash column chromatography (SiO_2_; EtOAc/hexanes = 1:9) to afford pure **18d** (53 mg, 71% yield) as a brown solid. After column chromatography unreacted 1,8-diiodonaphthalene (**16**, 210 mg, 77%) was recovered. Mp: 109.3–109.6 °C (CHCl_3_). *R*_*f*_ = 0.42 (EtOAc/hexanes = 1:4). ^1^H NMR (400 MHz; CDCl_3_) δ: 8.25 (1H, dd, *J* = 7.4, 1.2 Hz), 7.84–7.78 (3H, m), 7.61–7.58 (2H, m), 7.41–7.35 (3H, m), 7.08 (1H, t, *J* = 7.8 Hz), 5.78 (1H, s), 2.72 (1H, br s). ^13^C{^1^H} NMR (100 MHz; CDCl_3_) δ: 142.9, 138.7, 136.5, 134.9, 134.2, 132.0, 131.1, 130.3, 128.8, 128.5, 127.3, 125.5, 121.7, 99.5, 92.8, 86.6, 65.3. FTIR ν_max_ (ATR, film)/cm^–1^ 3365 (br), 1553, 1489, 1405, 1362, 1197, 1089, 1048, 1036, 1014. HRMS (ESI+) calcd for C_19_H_12_^35^ClINaO [M + Na]^+^, 440.9514; found, 440.9520.

#### 3-(8-Iodonaphthalen-1-yl)-1-(4-(trifluoromethyl)phenyl)prop-2-yn-1-ol (**18e**)

Product **18e** was prepared using 1,8-diiodonaphthalene (**16**, 214 mg, 0.56 mmol), alkyne **17e** (28.2 mg, 0.14 mmol), Pd(PPh_3_)_2_Cl_2_ (6.9 mg, 0.0099 mmol), CuI (3.8 mg, 0.019 mmol), and Et_3_N (5 mL) according to General Procedure A. The crude product was purified by flash column chromatography (SiO_2_; EtOAc/hexanes = 1:3) to afford pure **18e** (54.3 mg, 85% yield) as an orange solid. After column chromatography, unreacted 1,8-diiodonaphthalene (**16**, 151 mg, 71%) was recovered. Mp: 126–127 °C (CHCl_3_). *R*_*f*_ = 0.48 (EtOAc/hexanes = 1:3). ^1^H NMR (400 MHz; CDCl_3_) δ: 8.27 (1H, d, *J* = 7.4 Hz), 7.85–7.82 (3H, m), 7.80 (2H, d, *J* = 8.2 Hz), 7.68 (2H, d, *J* = 8.2 Hz), 7.42 (1H, t, *J* = 7.7 Hz), 7.11 (1H, t, *J* = 7.8 Hz), 5.86 (1H, d, *J* = 4.9 Hz), 2.64 (1H, d, *J* = 5.5 Hz). ^13^C{^1^H} NMR (100 MHz; CDCl_3_) δ: 144.0, 142.9, 136.6, 135.0, 132.1, 131.3, 130.5, 129.6, 128.6, 127.39, 127.36, 125.7 (q, ^3^*J*_C–F_ = 3.9 Hz), 125.5, 124.6, 122.9, 121.6, 99.1, 92.8, 86.9, 65.4. (Since the aromatic region is crowded, two quartet signals with ^13^C–^19^F couplings could not be identified with certainty). FTIR ν_max_ (ATR, film)/cm^–1^ 3267, 1408, 1323, 1159, 1107, 1067, 1047, 1034, 1014, 972, 967. HRMS (ESI+) calcd for C_20_H_12_F_3_INaO [M + Na]^+^, 474.9777; found, 474.9783.

#### 3-(8-Iodonaphthalen-1-yl)-1-(thiophen-2-yl)prop-2-yn-1-ol (**18f**)

Product **18f** was prepared using 1,8-diiodonaphthalene (**16**, 308 mg, 0.81 mmol), alkyne **17f** (28 mg, 0.20 mmol), Pd(PPh_3_)_2_Cl_2_ (10.0 mg, 0.014 mmol), CuI (5.4 mg, 0.028 mmol), and Et_3_N (6 mL) according to General Procedure A. The crude product was purified by flash column chromatography (SiO_2_; EtOAc/hexanes = 1:9 → 1:5) to afford pure **18f** (64.5 mg, 82% yield) as a reddish orange oil. After column chromatography unreacted 1,8-diiodonaphthalene (**16**, 223 mg, 72%) was recovered. *R*_*f*_ = 0.44 (EtOAc/hexanes = 1:5). ^1^H NMR (400 MHz; CDCl_3_) δ: 8.25 (1H, dd, *J* = 7.4, 1.3 Hz), 7.87 (1H, dd, *J* = 7.2, 1.4 Hz), 7.81–7.79 (2H, m), 7.40 (1H, dd, *J* = 8.1, 7.3 Hz), 7.33 (1H, dd, *J* = 5.1, 1.3 Hz), 7.31 (1H, dt, *J* = 3.5, 1.0 Hz), 7.08 (1H, t, *J* = 7.8 Hz), 7.01 (1H, dd, *J* = 5.0, 3.6 Hz), 6.02 (1H, d, *J* = 6.5 Hz), 2.77 (1H, d, *J* = 6.5 Hz). ^13^C{^1^H} NMR (100 MHz; CDCl_3_) δ: 144.3, 142.8, 136.6, 134.9, 132.1, 131.1, 130.3, 127.3, 126.9, 126.1, 125.9, 125.5, 121.7, 99.3, 92.9, 85.8, 61.8. FTIR ν_max_ (ATR, film)/cm^–1^ 3367, 1553, 1363, 1227, 1199, 1047, 1034, 997, 939. HRMS (ESI+) calcd for C_17_H_11_INaOS [M + Na]^+^, 412.9467; found, 412.9469.

#### 1-(Furan-2-yl)-3-(8-iodonaphthalen-1-yl)prop-2-yn-1-ol (**18g**)

Product **18g** was prepared using 1,8-diiodonaphthalene (**16**, 377 mg, 1.0 mmol), alkyne **18g** (30 mg, 0.25 mmol), Pd(PPh_3_)_2_Cl_2_ (12.3 mg, 0.018 mmol), CuI (6.7 mg, 0.035 mmol), and Et_3_N (6 mL) according to General Procedure A. The crude product was purified by flash column chromatography (SiO_2_; EtOAc/hexanes = 1:9 → 1:7→ 1:5) to afford pure **18g** (66 mg, 71% yield) as a red oil. After column chromatography, unreacted 1,8-diiodonaphthalene (**16**, 292 mg, 78%) was recovered. *R*_*f*_ = 0.37 (EtOAc/hexanes = 1:3). ^1^H NMR (400 MHz; CDCl_3_) δ: 8.25 (1H, dd, *J* = 7.4, 1.3 Hz), 7.86 (1H, dd, 7.2, 1.4 Hz), 7.81–7.76 (2H, m), 7.46 (1H, dd, *J* = 1.8, 0.8 Hz), 7.39 (1H, dd, *J* = 8.1, 7.3 Hz), 7.07 (1H, dd, *J* = 8.0, 7.5 Hz), 6.59 (1H, d, *J* = 3.3 Hz), 6.39 (1H, dd, *J* = 3.3, 1.9 Hz), 5.81 (1H, s), 2.73 (1H, s). ^13^C{^1^H} NMR (100 MHz; CDCl_3_) δ: 152.7, 143.1, 142.8, 136.6, 134.9, 132.2, 131.1, 130.3, 127.2, 125.4, 121.7, 110.6, 108.3, 97.7, 92.9, 85.5, 59.7. FTIR ν_max_ (ATR, film)/cm^–1^ 3385, 2363, 1553, 1495, 1362, 1143, 1047, 1005. HRMS (ESI+) calcd for C_17_H_11_INaO_2_ [M + Na]^+^, 396.9696; found, 396.9697.

#### 4-(8-Iodonaphthalen-1-yl)but-3-yn-2-one (**12h**)

Product **12h** was prepared using 1,8-diiodonaphthalene (**16**, 288 mg, 0.76 mmol), 3-butyn-2-one (**20a**, 13 mg, 15 μL, 0.19 mmol), Pd(PPh_3_)_2_Cl_2_ (9.1 mg, 0.013 mmol), CuI (5.1 mg, 0.027 mmol) DMSO (3 mL), and *i*-Pr_2_NEt (0.1 mL) according to General Procedure A. The crude product was purified by flash column chromatography (SiO_2_; EtOAc/hexanes = 1:19 → 1:9) to afford pure **12h** (32.4 mg, 53% yield) as an orange oil. After column chromatography, unreacted 1,8-diiodonaphthalene (**16**, 223 mg, 77%) was recovered. *R*_*f*_ = 0.53 (EtOAc/hexanes = 1:5). ^1^H NMR (400 MHz; CDCl_3_) δ: 8.31 (1H, dd, *J* = 7.4, 1.2 Hz), 7.98 (1H, dd, *J* = 7.3, 1.3 Hz), 7.91 (1H, dd, *J* = 8.2, 1.3 Hz), 7.86 (1H, dd, *J* = 8.2, 1.0 Hz), 7.45 (1H, dd, *J* = 8.1, 7.4 Hz), 7.15 (1H, t, *J* = 7.6 Hz), 2.51 (3H, s). ^13^C{^1^H} NMR (100 MHz; CDCl_3_) δ: 184.7, 143.3, 139.1, 135.0, 133.2, 132.7, 130.5, 127.7, 125.6, 119.7, 99.9, 92.7, 90.1, 31.9. FTIR ν_max_ (ATR, film)/cm^–1^ 2168, 1654, 1364, 1353, 1276, 1192, 1159, 1071, 970, 816, 757. HRMS (ESI+) calcd for C_14_H_10_IO [M + H]^+^, 320.9771; found, 320.9771.

#### 3-(8-Iodonaphthalen-1-yl)propiolamide (**12i**)

Product **12i** was prepared using 1,8-diiodonaphthalene (**16**, 440 mg, 1.16 mmol), propiolamide (**20b**, 20 mg, 0.29 mmol), Pd(PPh_3_)_2_Cl_2_ (14.2 mg, 0.020 mmol), CuI (7.7 mg, 0.041 mmol), DMSO (4 mL), and Et_3_N (1 mL) according to General Procedure A. The crude product was purified by flash column chromatography (SiO_2_; EtOAc/hexanes = 1:2 → 1:1→ 2:1) to afford pure **12i** (76.4 mg, 82% yield) as a pale brown solid. After column chromatography, unreacted 1,8-diiodonaphthalene (**16**, 343 mg, 78%) was recovered. *R*_*f*_ = 0.27 (EtOAc/hexanes = 1:1); 0.43 (EtOAc/hexanes = 2:1). ^1^H NMR (400 MHz; CDCl_3_) δ: 8.30 (1H, dd, *J* = 7.4, 1.2 Hz), 7.98 (1H, dd, *J* = 7.3, 1.4 Hz), 7.90 (1H, dd, *J* = 8.2, 1.3 Hz), 7.86 (1H, dd, *J* = 8.1, 1.1 Hz), 7.45 (1H, dd, *J* = 8.2, 7.3 Hz), 7.15 (1H, t, *J* = 7.8 Hz), 5.96 (2H, br s). ^13^C{^1^H} NMR (100 MHz; CDCl_3_) δ: 155.1, 144.2, 143.2, 138.5, 135.0, 132.8, 130.6, 127.7, 125.6, 119.6, 93.9, 92.7, 85.6. FTIR ν_max_ (ATR, film)/cm^–1^ 3454, 3352, 3194, 2925, 1650, 1598, 1440, 1396, 1280, 816. HRMS (ESI+) calcd for C_13_H_8_INNaO [M + Na]^+^, 343.9543; found, 343.9534.

#### 1-Iodo-8-(phenylethynyl)naphthalene (**12j**)

Product **12j** was prepared using 1,8-diiodonaphthalene (**16**, 149 mg, 0.39 mmol), phenylacetylene (10.0 mg, 10.8 μL, 0.098 mmol), Pd(PPh_3_)_2_Cl_2_ (4.8 mg, 0.0069 mmol), CuI (2.6 mg, 0.014 mmol), and Et_3_N (4 mL) according to General Procedure A. The crude product was purified by flash column chromatography (SiO_2_; hexanes) to afford pure **12j** (29.2 mg, 84% yield) as a yellow oil. After column chromatography, unreacted 1,8-diiodonaphthalene (**16**, 110 mg, 74%) was recovered. *R*_*f*_ = 0.43 (EtOAc/hexanes = 1:49). ^1^H NMR (400 MHz; CDCl_3_) δ: 8.31 (1H, dd, *J* = 7.3, 1.3 Hz), 7.93 (1H, dd, *J* = 7.2, 1.4 Hz), 7.84 (1H, dd, *J* = 8.2, 1.1 Hz), 7.81 (1H, dd, *J* = 8.2, 1.3 Hz), 7.68–7.65 (2H, m), 7.45 (1H, dd, *J* = 8.0, 7.4 Hz), 7.42–7.36 (3H, m), 7.11 (1H, t, *J* = 7.8 Hz). ^13^C{^1^H} NMR (100 MHz; CDCl_3_) δ: 142.8, 136.1, 135.0, 132.0, 131.0, 130.5, 130.3, 128.6, 128.5, 127.2, 125.6, 124.2, 123.0, 101.0, 93.1, 89.4. FTIR ν_max_ (ATR, film)/cm^–1^ 3054, 2923, 2851, 1597, 1551, 1489, 1441. HRMS (APCI+) Calcd for C_18_H_12_I [M + H]^+^, 354.9978; found, 354.9973.

#### 1-Iodo-8-((4-nitrophenyl)ethynyl)naphthalene (**12k**)

Product **12k** was prepared using 1,8-diiodonaphthalene (**16**, 517 mg, 1.36 mmol), 1-ethynyl-4-nitrobenzene (50.0 mg, 0.34 mmol), Pd(PPh_3_)_2_Cl_2_ (16.7 mg, 0.024 mmol), CuI (9.1 mg, 0.048 mmol), and Et_3_N (6 mL) according to General Procedure A. The crude product was purified by flash column chromatography (SiO_2_; EtOAc/hexanes = 1:19) to afford pure **12k** (116 mg, 86% yield) as a bright yellow solid. After column chromatography, unreacted 1,8-diiodonaphthalene (**16**, 391 mg, 76%) was recovered. Mp: 163.3–165.0 °C. *R*_*f*_ = 0.35 (EtOAc/hexanes = 1:5). ^1^H NMR (400 MHz; CDCl_3_) δ: 8.32 (1H, dd, *J* = 7.4, 1.2 Hz), 8.25 (2H, d, *J* = 8.9 Hz), 7.96 (1H, dd, *J* = 7.2, 1.3 Hz), 7.87 (2H, d, *J* = 8.2 Hz), 7.78 (2H, d, *J* = 8.9 Hz), 7.48 (1H, t, *J* = 7.7 Hz), 7.15 (1H, t, *J* = 7.8 Hz). ^13^C{^1^H} NMR (100 MHz; CDCl_3_) δ: 147.2, 143.1, 136.8, 135.1, 132.1, 131.7, 131.5, 130.9, 130.5, 127.6, 125.6, 123.9, 121.8, 98.9, 94.7, 92.8. FTIR ν_max_ (ATR, film)/cm^–1^ 2185, 1592, 1506, 1347, 1339, 1311. HRMS (ESI+) calcd for C_18_H_10_INNaO_2_ [M + Na]^+^, 421.9648; found, 421.9647.

### General Procedure B for the Oxidation of Propargyl Alcohols with MnO_2_

To a solution of propargyl alcohol in acetone (0.025 M), MnO_2_ (20 equiv) was added at 23 °C. The reaction mixture was stirred at this temperature until TLC indicated full consumption of alcohol which occurred within 1–2 h. The reaction mixture was then diluted with CH_2_Cl_2_ and filtered. SiO_2_ was added to the resulting solution, the solvent was removed under reduced pressure, and the obtained solid was loaded directly to the column. Purification by flash column chromatography on SiO_2_ afforded the desired ketone product.

#### 3-(8-Iodonaphthalen-1-yl)-1-phenylprop-2-yn-1-one (**12a**)

Product **12a** was obtained from alcohol **18a** (530 mg, 1.38 mmol) using MnO_2_ (2.82 g, 27.6 mmol) and acetone (30 mL) according to General Procedure B. The crude product was purified by flash column chromatography (SiO_2_; EtOAc/hexanes = 1:7 → 1:6 → 1:5 → 1:4) to afford pure **12a** (527 mg, 91% yield) as an orange oil. *R*_*f*_ = 0.23 (EtOAc/hexanes = 1:19). ^1^H NMR (400 MHz; CDCl_3_) δ: 8.32 (3H, app d, *J* = 7.4 Hz), 8.10 (1H, dd, *J* = 7.2, 1.3 Hz), 7.94 (1H, dd, *J* = 8.2, 0.9 Hz), 7.89 (1H, dd, *J* = 8.1, 0.8 Hz), 7.64 (1H, tt, *J* = 7.4, 1.3 Hz), 7.57–7.48 (3H, m), 7.18 (1H, dd, *J* = 8.0, 7.5 Hz). ^13^C{^1^H} NMR (100 MHz; CDCl_3_) δ: 178.1, 143.3, 138.6, 137.1, 135.1, 134.2, 133.0, 132.8, 130.4, 130.0 128.8, 127.8, 125.6, 120.3, 99.2, 93.1, 92.5. FTIR ν_max_ (ATR, film)/cm^–1^ 2174, 1632, 1597, 1578, 1449, 1363, 1339, 1313, 1286, 1226, 1170, 1046, 979, 817, 756, 698. HRMS (ESI+) calcd for C_19_H_12_IO [M + H]^+^, 382.9927; found, 382.9927.

#### 3-(8-Iodonaphthalen-1-yl)-1-(4-methoxyphenyl)prop-2-yn-1-one (**12b**)

Product **12b** was obtained from alcohol **18b** (15 mg, 0.036 mmol) using MnO_2_ (74 mg, 0.72 mmol) and acetone (1.5 mL) according to General Procedure B. The crude product was purified by flash column chromatography (SiO_2_; EtOAc/hexanes = 1:5 → 1:4) to afford pure **12b** (12.4 mg, 83% yield) as an orange oil. *R*_*f*_ = 0.53 (EtOAc/hexanes = 1:3). ^1^H NMR (400 MHz; CDCl_3_) δ: 8.31–8.27 (3H, m), 8.06 (1H, dd, *J* = 7.3, 1.4 Hz), 7.91 (1H, dd, *J* = 8.2, 1.1 Hz), 7.86 (1H, dd, *J* = 8.2, 1.2 Hz), 7.48 (1H, dd, *J* = 8.1, 7.3 Hz), 7.15 (1H, dd, *J* = 8.1, 7.4 Hz), 6.99 (2H, app d, *J* = 9.0 Hz), 3.90 (3H, s). ^13^C{^1^H} NMR (100 MHz; CDCl_3_) δ: 176.6, 164.5, 143.0, 138.3, 134.9, 132.6, 132.5, 132.2, 130.4, 130.2, 127.6, 125.4, 120.3, 113.9, 99.1, 93.0, 91.6, 55.6. FTIR ν_max_ (ATR, film)/cm^–1^ 2174, 1627, 1596, 1572, 1508, 1290, 1258, 1235, 1162, 1028. HRMS (ESI+) calcd for C_20_H_14_IO_2_ [M + H]^+^, 413.0033; found, 413.0041.

#### 1-(Benzo[*d*][1,3]dioxol-5-yl)-3-(8-iodonaphthalen-1-yl)prop-2-yn-1-one (**12c**)

Compound **12c** was obtained from alcohol **18c** (22 mg, 0.051 mmol) using MnO_2_ (102 mg, 1.0 mmol) and acetone (1.5 mL) according to General Procedure B. The crude product was purified by flash column chromatography (SiO_2_; EtOAc/hexanes = 1:3) to afford pure **12c** (17.3 mg, 79% yield) as a yellow oil. *R*_*f*_ = 0.66 (EtOAc/hexanes = 1:2). ^1^H NMR (400 MHz; CDCl_3_) δ: 8.30 (1H, dd, *J* = 7.4, 1.3 Hz), 8.06 (1H, dd, *J* = 7.3, 1.4 Hz), 8.01 (1H, dd, *J* = 8.2, 1.7 Hz), 7.93 (1H, dd, *J* = 8.3, 1.2 Hz), 7.87 (1H, dd, *J* = 8.2, 1.1 Hz), 7.69 (1H, d, *J* = 1.7 Hz), 7.49 (1H, dd, *J* = 8.1, 7.3 Hz), 7.16 (1H, dd, *J* = 8.0, 7.5 Hz), 6.92 (1H, d, *J* = 8.2 Hz), 6.08 (2H, s). ^13^C{^1^H} NMR (100 MHz; CDCl_3_) δ: 176.2, 153.0, 143.2, 138.4, 135.1, 132.9, 132.7, 132.4, 130.4, 128.7, 127.8, 127.7, 125.6, 120.4, 108.7, 108.2, 102.2, 99.1, 93.1, 91.8. FTIR ν_max_ (ATR, film)/cm^–1^ 2175, 1625, 1598, 1502, 1486, 1444, 1362, 1289, 1262, 1250, 1037. HRMS (ESI+) calcd for C_20_H_11_INaO_3_ [M + Na]^+^, 448.9645; found, 448.9652.

#### 1-(4-Chlorophenyl)-3-(8-iodonaphthalen-1-yl)prop-2-yn-1-one (**12d**)

To a solution of propargyl alcohol **18d** (25 mg, 0.06 mmol) in CH_2_Cl_2_ (1.5 mL), Dess-Martin periodinane (27.9 mg, 0.066 mmol) was added at 0 °C. The orange reaction mixture was stirred for 15 min at 0 °C and then warmed to 23 °C. The color of the reaction became cloudy yellow over time. The reaction mixture was stirred at 23 °C until TLC indicated full consumption of **18d** which occurred in 1 h. The reaction mixture was diluted with CH_2_Cl_2_ and filtered. The organic phase was washed with saturated aqueous solution of NaHCO_3_. The aqueous phase was extracted three times with DCM. The combined organic layer was dried over anhydrous Na_2_SO_4_, filtered, and concentrated under reduced pressure. The crude product was purified by flash column chromatography (SiO_2_; EtOAc/hexanes = 1:9 → 1:4) to afford pure **12d** (20 mg, 80% yield) as a bright orange solid. *R*_*f*_ = 0.36 (EtOAc/hexanes = 1:9). ^1^H NMR (400 MHz; CDCl_3_) δ: 8.31 (1H, dd, *J* = 7.4, 1.2 Hz), 8.27–8.23 (2H, m), 8.08 (1H, dd, *J* = 7.2, 1.4 Hz), 7.95 (1H, dd, *J* = 8.2, 1.3 Hz), 7.88 (1H, dd, *J* = 8.2, 1.1 Hz), 7.52–7.48 (3H, m), 7.18 (1H, t, *J* = 7.8 Hz). ^13^C{^1^H} NMR (100 MHz; CDCl_3_) δ: 176.7, 143.3, 141.8, 140.8, 138.7, 135.5, 135.1, 133.2, 131.3, 130.4, 129.2, 127.8, 125.6, 120.0, 98.8, 93.03, 93.01. FTIR ν_max_ (ATR, film)/cm^–1^ 2175, 1634, 1586, 1363, 1339, 1287, 1224, 1167, 1090, 981. HRMS (ESI+) calcd for C_19_H_10_^35^ClINaO [M + Na]^+^, 438.9357; found, 438.9358.

#### 3-(8-Iodonaphthalen-1-yl)-1-(4-(trifluoromethyl)phenyl)prop-2-yn-1-one (**12e**)

Product **12e** was obtained from alcohol **18e** (22.0 mg, 0.049 mmol) using MnO_2_ (100 mg, 0.98 mmol) and acetone (2.0 mL) according to General Procedure B. The crude product was purified by flash column chromatography (SiO_2_; EtOAc/hexanes = 1:9 → 1:7 → 1:5) to afford pure **12e** (13.7 mg, 63% yield) as a bright orange solid. *R*_*f*_ = 0.53 (EtOAc/hexanes = 1:5). ^1^H NMR (400 MHz; CDCl_3_) δ: 8.42 (2H, d, *J* = 8.1 Hz), 8.32 (1H, dd, *J* = 7.4, 1.2 Hz), 8.10 (1H, dd, *J* = 7.3, 1.4 Hz), 7.97 (1H, dd, *J* = 8.2, 1.4 Hz), 7.90 (1H, dd, *J* = 8.1, 1.2 Hz), 7.80 (2H, d, *J* = 8.1 Hz), 7.52 (1H, t, *J* = 7.7 Hz), 7.19 (1H, t, *J* = 7.8 Hz). ^13^C{^1^H} NMR (100 MHz; CDCl_3_) δ: 176.8, 143.4, 140.3, 138.9, 135.5, 135.1, 133.6, 133.4, 132.8, 131.9, 131.8, 130.5, 130.2, 130.0, 127.9, 126.1, 125.9 (q, ^3^*J*_C–F_ = 3.6 Hz), 125.6, 123.7 (q, ^1^*J*_C–F_ = 273 Hz), 119.8, 98.8, 93.9, 93.0. (since the aromatic region is crowded, one quartet signal with ^13^C–^19^F coupling could not be identified with certainty). FTIR ν_max_ (ATR, film)/cm^–1^ 2173, 1639, 1323, 1310, 1288, 1224, 1170, 1128, 1047, 1016, 982. HRMS (ESI+) calcd for C_20_H_10_F_3_INaO [M + Na]^+^, 472.9621; found, 472.9623.

#### 3-(8-Iodonaphthalen-1-yl)-1-(thiophen-2-yl)prop-2-yn-1-one (**12f**)

Compound **12f** was obtained from alcohol **18f** (20 mg, 0.051 mmol) using MnO_2_ (105 mg, 1.025 mmol) and acetone (2 mL) according to General Procedure B. The crude product was purified by flash column chromatography (SiO_2_; EtOAc/hexanes = 1:9) to afford pure **12f** (10.5 mg, 53% yield) as a bright orange-yellow oil. *R*_*f*_ = 0.41 (EtOAc/hexanes = 1:7). ^1^H NMR (400 MHz; CDCl_3_) δ: 8.30 (1H, d, *J* = 7.3 Hz), 8.16 (1H, dd, *J* = 3.8, 1.2 Hz), 8.05 (1H, dd, *J* = 7.2, 0.9 Hz), 7.92 (1H, dd, *J* = 8.2, 1.3 Hz), 7.87 (1H, dd, *J* = 8.1, 1.1 Hz), 7.73 (1H, dd, *J* = 5.0, 1.2 Hz), 7.48 (1H, t, *J* = 7.7 Hz), 7.21–7.14 (2H, m). ^13^C{^1^H} NMR (100 MHz; CDCl_3_) δ: 169.9, 145.1, 143.2, 138.7, 135.5, 135.2, 135.0, 133.0, 132.7, 130.4, 128.5, 127.8, 125.6, 120.0, 98.6, 93.1, 91.1. FTIR ν_max_ (ATR, film)/cm^–1^ 2178, 1610, 1514, 1410, 1363, 1301, 1231, 1051, 949. HRMS (ESI+) calcd for C_17_H_10_IOS [M + H]^+^, 388.9492; found, 388.9495.

#### 1-(Furan-2-yl)-3-(8-iodonaphthalen-1-yl)prop-2-yn-1-one (**12g**)

Product **12g** was obtained from alcohol **18g** (19.5 mg, 0.052 mmol) using MnO_2_ (106 mg, 1.04 mmol) and acetone (2.0 mL) according to General Procedure B. The crude product was purified by flash column chromatography (SiO_2_; EtOAc/hexanes = 1:9 → 1:7 → 1:5) to afford pure **12g** (17.4 mg, 90% yield) as a dark red oil. *R*_*f*_ = 0.42 (EtOAc/hexanes = 1:4). ^1^H NMR (400 MHz; CDCl_3_) δ: 8.31 (1H, dd, *J* = 7.4, 1.2 Hz), 8.06 (1H, dd, *J* = 7.3, 1.4 Hz), 7.94 (1H, dd, *J* = 8.2, 1.3 Hz), 7.88 (1H, dd, *J* = 8.2, 1.1 Hz), 7.71 (1H, dd, *J* = 1.6, 0.7 Hz), 7.58 (1H, dd, *J* = 3.6, 0.6 Hz), 7.49 (1H, d, *J* = 8.0, 7.4 Hz), 7.17 (1H, t, *J* = 7.7 Hz), 6.62 (1H, dd, *J* = 3.6, 1.7 Hz). ^13^C{^1^H} NMR (100 MHz; CDCl_3_) δ: 164.8, 153.4, 148.1, 143.2, 138.9, 135.1, 133.1, 132.8, 130.4, 127.8, 125.6, 121.5, 120.0, 112.8, 98.4, 93.0, 91.2. FTIR ν_max_ (ATR, film)/cm^–1^ 2365, 2179, 1624, 1461, 1393, 1306, 985. HRMS (ESI+) calcd for C_17_H_9_INaO_2_ [M + Na]^+^, 394.9539; found, 394.9526.

### General Procedure C for the Syntheses of Fluoranthenes

In a 25 mL, round-bottomed flask, a 1-iodo-8-alkynylnaphthalene derivative (1.0 equiv) was dissolved in 1.0 mL of 1,4-dioxane at 23 °C under N_2_. To this solution 2-furylboronic acid (**19**, 2.0 equiv), K_3_PO_4_ (3.0 equiv), and Pd(PPh_3_)_4_ (5 mol %) were added. Afterward, 1.0 mL of 1,4-dioxane and 1.0 mL of H_2_O were added along the walls of the flask. The resulting reaction mixture was heated to 100 °C in an oil bath and stirred under reflux for 3–6 h. The reaction mixture was cooled to 23 °C and quenched with H_2_O (5 mL). The aqueous phase was extracted with EtOAc (3 × 10 mL). The combined organic phase was dried over anhydrous Na_2_SO_4_, filtered, and concentrated under reduced pressure. The residue was purified by flash column chromatography on SiO_2_.

#### (8-Hydroxyfluoranthen-7-yl)(phenyl)methanone (**15a**)

Fluoranthene product **15a** was synthesized using alkyne **12a** (18.5 mg, 0.048 mmol), 2-furylboronic acid (**19**, 10.8 mg, 0.096 mmol), K_3_PO_4_ (30.8 mg, 0.145 mmol), and Pd(PPh_3_)_4_ (2.8 mg, 0.0024 mmol) according to General Procedure C. The crude product was purified by flash column chromatography (SiO_2_; EtOAc/hexanes = 1:19 → 1:9 → 1:4) to afford pure **15a** (14.4 mg, 92% yield) as a yellow-green amorphous solid. *R*_*f*_ = 0.52 (EtOAc/hexanes = 1:2). ^1^H NMR (400 MHz; CDCl_3_) δ: 8.83 (1H, s), 7.98 (3 H, app t, *J* = 7.0 Hz), 7.88 (1H, d, *J* = 7.0 Hz), 7.76 (1H, d, *J* = 8.2 Hz), 7.69 (1H, d, *J* = 8.1 Hz), 7.63–7.54 (2H, m), 7.39 (2H, t, *J* = 7.8 Hz), 7.17 (1H, t, *J* = 7.7 Hz), 7.09 (1H, d, *J* = 8.6 Hz), 6.78 (1H, d, *J* = 7.2 Hz). ^13^C{^1^H} NMR (100 MHz; CDCl_3_) δ: 199.3, 158.0, 139.4, 138.3, 135.9, 135.7, 134.1, 132.54, 132.52, 130.7, 129.9, 129.0, 128.0, 127.7, 127.5, 126.4, 126.3, 126.1, 125.7, 119.5, 116.5. FTIR ν_max_ (ATR, film)/cm^–1^ 3359, 1653, 1581, 1449, 1440, 1395, 1316, 1286, 1226, 815, 773. HRMS (ESI−) calcd for C_23_H_13_O_2_ [M – H]^−^, 321.0921; found, 321.0917.

#### (8-Hydroxyfluoranthen-7-yl)(4-methoxyphenyl)methanone (**15b**)

Fluoranthene product **15b** was synthesized using alkyne **12b** (28.5 mg, 0.070 mmol), 2-furylboronic acid (**19**, 15.5 mg, 0.14 mmol), K_3_PO_4_ (44 mg, 0.21 mmol), and Pd(PPh_3_)_4_ (4.0 mg, 0.0035 mmol) according to General Procedure C. The crude product was purified by flash column chromatography (SiO_2_; EtOAc/hexanes = 1:9 → 1:1) to afford pure **15b** (17.8 mg, 73% yield) as a brown oil. *R*_*f*_ = 0.37 (EtOAc/hexanes = 1:2); 0.71 (EtOAc/hexanes = 1:1). ^1^H NMR (400 MHz; CDCl_3_) δ: 8.47 (1H, s), 7.97–7.93 (3H, m), 7.87 (1H, d, *J* = 6.9 Hz), 7.75 (1H, d, *J* = 8.2 Hz), 7.70 (1H, d, *J* = 8.1 Hz), 7.60 (1H, dd, *J* = 8.0, 7.0 Hz), 7.24 (1H, t, *J* = 7.7 Hz), 7.06 (1H, d, *J* = 8.2 Hz), 6.92 (1H, d, *J* = 7.2 Hz), 6.85 (2H, d, *J* = 8.9 Hz), 3.83 (3H, s). ^13^C{^1^H} NMR (100 MHz; CDCl_3_) δ: 197.3, 164.6, 157.3, 139.1, 136.0, 135.7, 133.1, 132.6, 132.4, 130.9, 128.0, 127.8, 127.3, 126.2, 125.9, 125.7, 120.1, 119.4, 116.3, 114.3, 114.1, 55.7. FTIR ν_max_ (ATR, film)/cm^–1^ 3317 (br), 1643, 1594, 1439, 1262, 1159. HRMS (ESI−) calcd for C_24_H_15_O_3_ [M – H]^−^, 351.1027; found, 351.1028.

#### Benzo[*d*][1,3]dioxol-5-yl(8-hydroxyfluoranthen-7-yl)methanone (**15c**)

Fluoranthene product **15c** was synthesized using alkyne **12c** (24.0 mg, 0.056 mmol), 2-furylboronic acid (**19**, 12.6 mg, 0.113 mmol), K_3_PO_4_ (36 mg, 0.17 mmol), and Pd(PPh_3_)_4_ (3.3 mg, 0.0028 mmol) according to General Procedure C. The crude product was purified by flash column chromatography (SiO_2_; EtOAc/hexanes = 1:4 → 1:3 → 1:2) to afford pure **15c** (18.6 mg, 90% yield) as a yellow-brown oil.

In another experiment, the reaction between alkyne **12c** (16.0 mg, 0.038 mmol) and furan-2-boronic acid pinacol ester (14.6 mg, 0.075 mmol) in the presence of K_3_PO_4_ (24.2 mg, 0.114 mmol) and Pd(PPh_3_)_4_ (2.2 mg, 0.0019 mmol) following General Procedure C afforded pure hydroxyfluoranthene **15c** (12.4 mg) in 89% yield. *R*_*f*_ = 0.36 (EtOAc/hexanes = 1:2). ^1^H NMR (400 MHz; CDCl_3_) δ: 8.17 (1H, br s), 7.92 (1H, d, *J* = 8.3 Hz), 7.87 (1H, d, *J* = 6.8 Hz), 7.76 (1H, d, *J* = 8.2 Hz), 7.73 (1H, d, *J* = 8.1 Hz), 7.60 (1H, dd, *J* = 8.2, 6.9 Hz), 7.53 (2H, m), 7.30 (1H, dd, *J* = 8.1, 7.2 Hz), 7.04 (2H, t, *J* = 7.3 Hz), 6.70 (1H, d, *J* = 8.2 Hz), 6.05 (2H, s). ^13^C{^1^H} NMR (100 MHz; CDCl_3_) δ: 196.8, 156.8, 152.9, 148.6, 139.0, 136.0, 135.7, 132.64, 132.59, 132.5, 129.9, 128.3, 128.0, 127.8, 127.4, 126.3, 125.6, 125.5, 120.4, 119.5, 116.2, 109.4, 108.4, 102.2. FTIR ν_max_ (ATR, film)/cm^–1^ 3327, 1648, 1599, 1583, 1503, 1485, 1441, 1396, 1288, 1263, 1247, 1096, 1038, 815, 773. HRMS (ESI+) calcd for C_24_H_15_O_4_ [M + H]^+^, 367.0965; found, 367.0963.

#### (4-Chlorophenyl)(8-hydroxyfluoranthen-7-yl)methanone (**15d**)

Fluoranthene product **15d** was synthesized using alkyne **12d** (20 mg, 0.048 mmol), 2-furylboronic acid (**19**, 10.7 mg, 0.096 mmol), K_3_PO_4_ (30.6 mg, 0.144 mmol), and Pd(PPh_3_)_4_ (2.8 mg, 0.0024 mmol) according to General Procedure C. The crude product was purified by flash column chromatography (SiO_2_; EtOAc/hexanes = 1:9 → 1:5) to afford pure **15d** (11.8 mg, 69% yield) as a brown oil. *R*_*f*_ = 0.53 (EtOAc/hexanes = 1:2). ^1^H NMR (400 MHz; CDCl_3_) δ: 8.53 (1H, s), 7.97 (1H, d, *J* = 8.3 Hz), 7.92–7.87 (3H, m), 7.77 (1H, d, *J* = 8.2 Hz), 7.73 (1H, d, *J* = 8.1 Hz), 7.61 (1H, dd, *J* = 8.2, 6.9 Hz), 7.38–7.35 (2H, m), 7.25 (1H, dd, *J* = 8.1, 7.2 Hz), 7.07 (1H, d, *J* = 8.3 Hz), 6.86 (1H, d, *J* = 7.2 Hz). ^13^C{^1^H} NMR (100 MHz; CDCl_3_) δ: 197.7, 157.6, 140.6, 139.2, 136.5, 135.8, 135.4, 132.64, 132.55, 132.0, 131.9, 129.9, 129.4, 128.1, 127.73, 127.67, 126.5, 126.3, 125.9, 119.7, 116.5. FTIR ν_max_ (ATR, film)/cm^–1^ 3361 (br), 1654, 1584, 1439, 1398, 1310, 1286, 1226, 1091. HRMS (ESI-) calcd for C_23_H_12_^35^ClO_2_ [M – H]^−^: 355.0531; found, 355.0532; calcd for C_23_H_12_^37^ClO_2_ [M – H]^−^, 357.0502, found 357.0502.

#### (8-Hydroxyfluoranthen-7-yl)(4-(trifluoromethyl)phenyl)methanone (**15e**)

Fluoranthene product **15e** was synthesized using alkyne **12e** (13.0 mg, 0.029 mmol), 2-furylboronic acid (**19**, 6.5 mg, 0.058 mmol), K_3_PO_4_ (18.5 mg, 0.087 mmol), and Pd(PPh_3_)_4_ (1.7 mg, 0.0015 mmol) according to General Procedure C. The crude product was purified by flash column chromatography (SiO_2_; EtOAc/hexanes = 1:5) to afford pure **15e** (5.9 mg, 52% yield) as a yellow oil. *R*_*f*_ = 0.49 (EtOAc/hexanes = 1:2). ^1^H NMR (400 MHz; CDCl_3_) δ: 8.62 (1H, br s), 8.07 (2H, d, *J* = 8.2 Hz), 8.02 (1H, br d, *J* = 7.9 Hz), 7.90 (1H, d, *J* = 6.8 Hz), 7.78 (1H, d, *J* = 8.0 Hz), 7.73 (1H, d, *J* = 8.1 Hz), 7.67–7.61 (3H, m), 7.20 (1H, app t, *J* = 7.7 Hz), 7.10 (1H, br d, *J* = 7.2 Hz), 6.78 (1H, d, *J* = 7.2 Hz). ^13^C{^1^H} NMR (100 MHz; CDCl_3_) δ: 197.9, 157.9, 141.0, 140.1, 140.0, 139.3, 135.7, 135.5, 135.3, 135.0, 132.8, 132.5, 130.9, 130.8, 129.9, 128.2, 127.8, 127.6, 126.7, 126.6, 126.0 (q, ^3^*J*_C–F_ = 3.7 Hz), 125.8, 119.8, 119.0, 116.6. (Since the aromatic region is crowded, two quartet signals with ^13^C–^19^F couplings could not be identified with certainty). ^19^F{^1^H} NMR (376 MHz; CDCl_3_) δ: −62.0. FTIR ν_max_ (ATR, film)/cm^–1^ 3380 (br), 2922, 2852, 1582, 1440, 1324, 1285, 1172, 1132, 1110. HRMS (ESI−) calcd for: C_24_H_12_F_3_O_2_ [M – H]^−^, 389.0795; found, 389.0794.

#### (8-Hydroxyfluoranthen-7-yl)(thiophen-2-yl)methanone (**15f**)

Fluoranthene product **15f** was synthesized using alkyne **12f** (17.0 mg, 0.044 mmol), 2-furylboronic acid (**19**, 9.9 mg, 0.088 mmol), K_3_PO_4_ (28 mg, 0.13 mmol), and Pd(PPh_3_)_4_ (2.5 mg, 0.0022 mmol) according to General Procedure C. The crude product was purified by flash column chromatography (SiO_2_; EtOAc/hexanes = 1:5 → 1:3) to afford pure **15f** (13.1 mg, 91% yield) as a dark yellow solid. *R*_*f*_ = 0.48 (EtOAc/hexanes = 1:2). ^1^H NMR (400 MHz; CDCl_3_) δ: 7.99 (1H, br s), 7.94 (1H, d, *J* = 8.1 Hz), 7.87 (1H, d, *J* = 6.9 Hz), 7.76 (3H, m), 7.61 (2H, m), 7.32 (1H, dd, *J* = 8.1, 7.2 Hz), 7.14 (1H, d, *J* = 7.1 Hz), 7.05 (1H, d, *J* = 8.1 Hz), 6.95 (1H, dd, *J* = 4.8, 3.9 Hz). ^13^C{^1^H} NMR (100 MHz; CDCl_3_) δ: 190.1, 156.4, 144.0, 138.9, 137.4, 136.0, 135.9, 135.7, 132.8, 132.6, 130.0, 128.7, 128.1, 127.8, 127.5, 126.4, 125.8, 125.5, 120.4, 119.6, 116.2. FTIR ν_max_ (ATR, film)/cm^–1^ 3332 (br), 1625, 1582, 1509, 1453, 1439, 1408, 1396, 1379, 1354, 1309, 1288, 1228, 1210, 1055, 1039, 904. HRMS (ESI−) calcd for C_21_H_11_O_2_S [M – H]^−^, 327.0485; found, 327.0486.

#### Furan-2-yl(8-hydroxyfluoranthen-7-yl)methanone (**15g**)

Fluoranthene product **15g** was synthesized using alkyne **12g** (16.0 mg, 0.043 mmol), 2-furylboronic acid (**19**, 9.6 mg, 0.086 mmol), K_3_PO_4_ (27.4 mg, 0.13 mmol), and Pd(PPh_3_)_4_ (2.5 mg, 0.0022 mmol) according to General Procedure C. The crude product was purified by flash column chromatography (SiO_2_; EtOAc/hexanes = 1:4 → 1:3 → 1:2) to afford pure **15g** (9.7 mg, 72% yield) as a bright yellow solid. *R*_*f*_ = 0.43 (EtOAc/hexanes = 1:2). ^1^H NMR (400 MHz; CDCl_3_) δ: 8.49 (1H, br s), 7.96 (1H, d, *J* = 8.3 Hz), 7.87 (1H, d, *J* = 6.9 Hz), 7.79 (1H, d, *J* = 8.2 Hz), 7.77 (1H, d, *J* = 8.2 Hz), 7.62 (1H, dd, *J* = 8.2, 6.9 Hz), 7.54 (1H, s), 7.39–7.30 (2H, m), 7.05 (1H, d, *J* = 8.3 Hz), 6.95 (1H, d, *J* = 7.1 Hz), 6.54 (1H, dd, *J* = 3.6, 1.6 Hz). ^13^C{^1^H} NMR (100 MHz; CDCl_3_) δ: 184.9, 157.6, 147.8, 139.4, 136.1, 136.0, 132.7, 132.5, 130.0, 128.1, 127.9, 127.5, 126.5, 126.3, 125.0, 121.9, 119.6, 116.2, 114.5, 113.3, 112.7. FTIR ν_max_ (ATR, film)/cm^–1^ 3305, 1636, 1584, 1459, 1439, 1395, 1314, 1281, 1054, 1023, 817, 772. HRMS (ESI+) Calcd for C_21_H_13_O_3_ [M + H]^+^, 313.0860; found, 313.0855.

#### 1-(8-Hydroxyfluoranthen-7-yl)ethan-1-one (**15h**)

Fluoranthene product **15h** was synthesized using alkyne **12h** (20.5 mg, 0.063 mmol), 2-furylboronic acid (**19**, 14.0 mg, 0.125 mmol), K_3_PO_4_ (40.1 mg, 0.189 mmol), and Pd(PPh_3_)_4_ (3.6 mg, 0.0032 mmol) according to General Procedure C. The crude product was purified by flash column chromatography (SiO_2_; EtOAc/hexanes = 1:5) to afford pure **15h** (6.4 mg, 38% yield) as an orange solid. *R*_*f*_ = 0.36 (EtOAc/hexanes = 1:3). ^1^H NMR (400 MHz; CDCl_3_) δ: 9.71 (1H, br s), 7.97 (1H, d, *J* = 7.2 Hz), 7.91 (2H, dd, *J* = 8.1, 7.2 Hz), 7.86 (1H, d, *J* = 6.9 Hz), 7.82 (1H, d, *J* = 8.2 Hz), 7.63 (2H, ddd, *J* = 8.2, 7.1, 2.4 Hz), 6.99 (1H, d, *J* = 8.3 Hz), 2.91 (3H, s). ^13^C{^1^H} NMR (100 MHz; CDCl_3_) δ: 206.1, 157.7, 138.8, 135.9, 135.7, 132.7, 132.5, 130.1, 128.3, 127.9, 126.8, 126.5, 125.5, 121.0, 119.7, 116.6, 31.1. FTIR ν_max_ (ATR, film)/cm^–1^ 3321, 1685, 1580, 1438, 1396, 1285, 1223, 814, 772. HRMS (ESI−) calcd for C_18_H_12_O_2_ [M]^−^, 260.0843; found, 260.0859.

#### 8-Hydroxyfluoranthene-7-carboxamide (**15i**)

Fluoranthene product **15i** was synthesized using alkyne **12i** (30.0 mg, 0.093 mmol), 2-furylboronic acid (**19**, 20.9 mg, 0.187 mmol), K_3_PO_4_ (59.2 mg, 0.28 mmol), and Pd(PPh_3_)_4_ (5.4 mg, 0.0047 mmol) according to General Procedure C. The crude product was purified by flash column chromatography (SiO_2_; EtOAc/hexanes = 1:2 → 1:1) to afford pure **15i** (13.0 mg, 53% yield) as a light yellow solid. Mp: 201.5–203.0 °C. *R*_*f*_ = 0.61 (EtOAc only). ^1^H NMR (400 MHz; CDCl_3_) δ: 10.53 (1H, br s), 8.45 (1H, d, *J* = 7.2 Hz), 7.91 (1H, d, *J* = 8.3 Hz), 7.90 (1H, d, *J* = 8.2 Hz), 7.85 (1H, d, *J* = 6.9 Hz), 7.81 (1H, d, *J* = 6.9 Hz), 7.63 (2H, m), 7.02 (1H, d, *J* = 8.4 Hz), 6.48 (2H, br s). ^13^C{^1^H} NMR (100 MHz; CDCl_3_) δ: 171.8, 159.6, 137.0, 136.0, 135.6, 132.6, 132.5, 130.3, 128.5, 128.4, 127.8, 126.4, 126.2, 124.8, 119.6, 116.9, 113.2. FTIR ν_max_ (ATR, film)/cm^–1^ 3454, 3352, 3194, 1650, 1598, 1440, 1396, 1280, 1228, 816, 773. HRMS (ESI+) calcd for C_17_H_12_NO_2_ [M + H]^+^, 262.0863; found, 262.0863.

#### 2-(8-(Phenylethynyl)naphthalen-1-yl)furan (**13j**)

Compound **13j** was synthesized using **12j** (33.4 mg, 0.094 mmol), 2-furylboronic acid (**19**, 21.1 mg, 0.184 mmol), K_3_PO_4_ (60.1 mg, 0.282 mmol), and Pd(PPh_3_)_4_ (5.5 mg, 0.0047 mmol) according to General Procedure C. The crude product was purified by flash column chromatography (SiO_2_; EtOAc/hexanes = 1:19 → 1:9 → 1:7) to afford pure **13j** (25.7 mg, 92% yield) as an orange oil. *R*_f_ = 0.54 (EtOAc/hexanes = 1:19). ^1^H NMR (400 MHz; CDCl_3_) δ: 8.05–7.72 (4H, m), 7.56 (1H, dd, *J* = 6.9, 1.0 Hz), 7.51–7.45 (3H, m), 7.34 (2H, dd, *J* = 6.9, 2.0 Hz), 7.30–7.26 (2H, m), 6.56 (1H, d, *J* = 3.3 Hz), 6.45 (1H, dd, *J* = 3.0, 1.9 Hz). ^13^C{^1^H} NMR (100 MHz; CDCl_3_) δ: 154.7, 142.3, 135.0, 134.6, 132.0, 131.9, 131.2, 130.4, 129.4, 129.2, 128.0, 127.8, 125.6, 125.4, 124.1, 120.7, 111.9, 109.0, 95.0, 88.8. FTIR ν_max_ (ATR, film)/cm^–1^: 3055, 2924, 1597, 1568, 1508, 1489, 1442, 1426, 1219, 1207, 1076, 1029, 953, 914, 886, 806, 755. HRMS (APCI+) calcd, C_22_H_15_O [M + H]^+^ 295.1117; found, 295.1106.

#### 7-Phenylfluoranthen-8-ol (**15j**)

Compound **13j** (7.8 mg, 0.026 mmol) was dissolved in mesitylene (0.9 mL) in a 10 mL round-bottomed flask. The resulting yellow solution was heated at 130 °C in an oil bath for 43 h. Then the reaction mixture was allowed to cool down to 23 °C and quenched with H_2_O. The aqueous phase was extracted four times with CH_2_Cl_2_. The combined organic layer was dried over anhydrous Na_2_SO_4_, filtered, and concentrated under reduced pressure. The crude product was purified by flash column chromatography (SiO_2_; EtOAc/hexanes = 1:19 → 1:9) to afford pure **15j** (4.3 mg, 55% yield) as an orange-yellow solid. *R*_f_ = 0.41 (EtOAc/hexanes = 1:4). ^1^H NMR (400 MHz; CDCl_3_) δ: 7.87 (1H, d, *J* = 6.9 Hz), 7.83 (1H, d, *J* = 8.1 Hz), 7.75 (1H, d, *J* = 8.1 Hz), 7.73 (1H, d, *J* = 8.0 Hz), 7.67–7.55 (6H, m), 7.30 (1H, t, *J* = 7.6 Hz), 7.03 (1H, d, *J* = 8.2 Hz), 6.74 (1H, d, *J* = 7.1 Hz), 4.98 (1H, s). ^13^C{^1^H} NMR (100 MHz; CDCl_3_) δ: 153.1, 138.8, 136.73, 136.71, 134.4, 133.0, 132.6, 130.4, 130.1, 130.0, 129.2, 128.1, 127.8, 126.9, 125.8, 124.9, 122.7, 122.1, 119.1, 114.2. FTIR ν_max_ (ATR, film)/cm^–1^: 3508, 3432, 3058, 2923, 1603, 1443, 1267, 1207, 1198, 1173, 816, 774, 712. HRMS (APCI−) calcd: C_22_H_13_O^–^ [M – H]^−^, 293.0972; found, 293.0977.

#### 7-(4-Nitrophenyl)fluoranthen-8-ol (**15k**)

Fluoranthene product **15k** was synthesized using alkyne **12k** (16.3 mg, 0.041 mmol), 2-furylboronic acid (**19**, 9.2 mg, 0.0.082 mmol), K_3_PO_4_ (26.0 mg, 0.123 mmol), and Pd(PPh_3_)_4_ (2.4 mg, 0.0020 mmol) according to General Procedure C. The crude product was purified by flash column chromatography (SiO_2_; EtOAc/hexanes = 1:19 → 1:9) to afford pure **15k** (3.5 mg, 25% yield) as an orange solid. *R*_*f*_ = 0.26 (EtOAc/hexanes = 1:3). ^1^H NMR (400 MHz; CDCl_3_) δ: 8.49 (2H, d, *J* = 8.7 Hz), 7.90 (1H, d, *J* = 6.9 Hz), 7.86 (1H, d, *J* = 8.2 Hz), 7.81–7.74 (4H, m), 7.63 (1H, dd, *J* = 8.2, 6.9 Hz), 7.33 (1H, dd, *J* = 8.1, 7.2 Hz), 6.99 (1H, d, *J* = 8.1 Hz), 6.75 (1H, d, *J* = 7.1 Hz), 4.84 (1H, s). ^13^C{^1^H} NMR (100 MHz; CDCl_3_) δ: 152.4, 148.2, 142.6, 138.6, 136.2, 136.0, 133.2, 132.9, 131.7, 130.1, 128.2, 127.8, 127.5, 126.2, 124.8, 123.2, 122.71, 122.68, 119.5, 114.7. FTIR ν_max_ (ATR, film)/cm^–1^ 3463, 2924, 1600, 1516, 1478, 1391, 1334, 1080, 853. HRMS (ESI−) Calcd for C_22_H_12_NO_3_ [M – H]^−^, 338.0823; found, 338.0821.

### Crystallization of Fluoranthene **15i** for Single-Crystal X-ray Analysis

Compound **15i** (6 mg) was dissolved in CH_2_Cl_2_ (1.0 mL) in a 2 mL vial. This vial was placed in a 20 mL scintillation vial containing pentane (3 mL), and the outer vial was sealed with a screw cap. Crystals were obtained within 1 week at room temperature.

## Supporting Information Available

FAIR data, including the primary NMR FID files, for compounds **12a**–**k**, **13j**, **15a**–**k**, **17a**–**g**, **18a**–**g**, and **21** (ZIP)
Experimental procedures and characterization data for **16**, **17a**–**g**, and **21**; X-ray analysis data of **15i**; and NMR spectra for all synthesized compounds (PDF)

## Accession Codes

CCDC 2128363 contains the supplementary crystallographic data for this paper. These data can be obtained free of charge via www.ccdc.cam.ac.uk/data_request/cif, or by emailing data_request@ccdc.cam.ac.uk, or by contacting The Cambridge Crystallographic Data Centre, 12 Union Road, Cambridge CB2 1EZ, UK; fax: +44 1223 336033.
