# Supplementary material for: Rapid Access to Hydroxyfluoranthenes via a Domino Suzuki–Miyaura/Intramolecular Diels–Alder/Ring-Opening Reactions Sequence
Source: J Org Chem. 2022 Apr 7;87(9):6336–46. doi: 10.1021/acs.joc.1c03080 (PMC9087347; doi:10.1021/acs.joc.1c03080)
Supplement: Supplementary file 2 — jo1c03080_si_002.pdf [file jo1c03080_si_002.pdf]

Rapid Access to Hydroxyfluoranthenes via a Domino Suzuki-Miyaura/Intramolecular Diels-Alder/Ring-Opening Reactions Sequence

Supporting Information

Dilgam Ahmadli,[†] Yesim Sahin,[†] Eylul Calikyilmaz,[†] Onur Şahin,[¶] and Yunus E. Türkmen*[,†,‡]

[†]*Department of Chemistry, Faculty of Science, Bilkent University, Ankara, 06800, Turkey*

[¶] *Department of Occupational Health & Safety, Faculty of Health Sciences, Sinop University, Sinop, 57000, Turkey*

[‡] *UNAM — National Nanotechnology Research Center, Institute of Materials Science and Nanotechnology, Bilkent University, Ankara, 06800, Turkey*

Table of Contents

S-2 Preparation of 1,8-Diiodonaphthalene (**16**)
S-3 Synthesis of Propargyl Alcohols **17**
S-10 Studies on the Oxidation of Propargylic Alcohols
S-11 X-Ray Diffraction Analysis of fluoranthene **15i**
S-13 References
S-14 ^{1}H- and ^{13}C-NMR Spectra

Preparation of 1,8-Diiodonaphthalene (16):

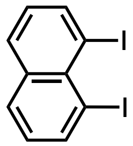

16

A 250 mL, 3-neck, round-bottomed flask was charged with 1,8-diaminonaphthalene (1.00 g, 6.32 mmol) and cooled down to ca. -15 °C in an ice/NaCl bath. Then it was dissolved in 11.6 mL 6.9 M H_2SO_4(aq). To this solution, $NaNO_2$ (1.308 g, 18.96 mmol, dissolved in 5 mL H_2O) was added dropwise resulting in the formation of a brown gas. Then, KI (6.029 g, 37.92 mmol, dissolved in 5 mL H_2O) was added dropwise at -15 °C. The resulting reaction mixture was heated quickly to 85 °C in an oil bath, and stirred at this temperature for 45 min. It was then cooled to 23 °C and neutralized with NaOH pellets. The resulting solid was filtered off with suction and then extracted with DCM in a Soxhlet apparatus for 10 h. The resulting extract was sequentially washed with 10% HCl solution, saturated aqueous $Na_2S_2O_3$ solution and 1M NaOH solution. Then, the organic phase was dried over anhydrous Na_2SO_4, filtered and concentrated under reduced pressure. Purification by column chromatography (SiO_2; hexanes only) gave pure 1,8-diiodonaphthalene **16** (1.79 g, 75% yield) as a yellow solid. Spectral data are in agreement with the values reported in the literature.[1]

R_f = 0.41 (Only hexanes)

TLC Visualization: UV active; stains with $KMnO_4$ solution.

^{1}H NMR (400 MHz; $CDCl_3$) δ: 8.39 (2H, d, *J* = 7.3 Hz), 7.79 (2H, d, *J* = 8.0 Hz), 7.03 (2H, t, *J* = 7.7 Hz).

^{13}C{^{1}H} NMR (100 MHz; $CDCl_3$) δ: 144.1, 135.8, 132.1, 131.1, 127.0, 96.2.

IR ν_{maks} (ATR, solid)/cm^{-1}: 3051, 2923, 2853, 1532, 1488, 1417.

HRMS (APCI+) Calcd for $C_{10}H_6I_2$ $[M]^+$: 379.8554; found: 379.8562.

Synthesis of Propargyl Alcohols 17:

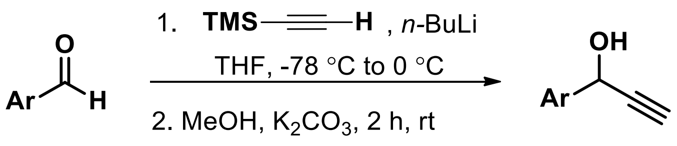

Compounds **17a-g** were synthesized following a reported literature procedure.[2]

General Procedure D for the synthesis of Alcohols 17:

An oven-dried, 2-neck round-bottomed flask was cooled under vacuum and refilled with N_2 (×3). Trimethylsilylacetylene (1.1 equiv) was dissolved in 10 mL of anhydrous THF under N_2. The resulting solution was then cooled to -78 °C and stirred for 10 min. Afterwards, *n*-BuLi (1.6 M in hexane, 1.06 equiv) was added dropwise at -78 °C, and the reaction flask was warmed to 0 °C after 10 min. After stirring this mixture for 20 min at 0 ºC, the reaction mixture was cooled back to -78 °C. After 10 min of stirring at this temperature, a solution of aldehyde (1 equiv) in 3 mL of anhydrous THF was added dropwise at -78 °C. The reaction mixture was warmed to 0 °C after 10 min and then was gradually allowed to warm to 23 °C. After 2 h of stirring, MeOH (10 ml) and K_2CO_3 (196 mg, 1.42 mmol) were added at 23 °C. The resulting reaction mixture was stirred for 2 h at 23 °C and then quenched with saturated aqueous NH_4Cl. The aqueous phase was extracted with CH_2Cl_2 (3×15 mL). The combined organic phase was dried over anhydrous Na_2SO_4, filtered and concentrated under reduced pressure. The residue was purified by flash column chromatography.

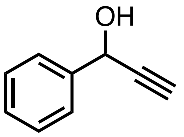

17a

Alkyne **17a** was prepared using benzaldehyde (500 mg, 479 μL, 4.71 mmol), trimethylsilylacetylene (513 mg, 724 μL, 5.22 mmol), *n*-BuLi (1.6 M in hexane, 3.12 mL, 4.99 mmol) and THF (13 mL) according to General Procedure D. The crude product was purified by flash column chromatography (SiO_2; EtOAc:hexanes = 1:9 → 1:8 → 1:5) to afford pure **17a** (556 mg, 89% yield) as colorless oil. Spectral data are in agreement with the values reported in the literature.[3]

$\boldsymbol{R_f}$ = 0.31 (EtOAc:hexanes = 1:5)

TLC Visualization: UV active; stains with $KMnO_4$ solution.

^{1}H NMR (400 MHz; $CDCl_3$) δ: 7.57-7.55 (2H, m) 7.42-7.33 (3H, m), 5.47 (1H, dd, *J* = 6.2, 2.2 Hz), 2.68 (1H, d, *J* = 2.3 Hz), 2.44 (1H, br d, *J* = 4.7 Hz).

^{13}C{^{1}H} NMR (100 MHz; $CDCl_3$) δ: 140.2, 128.8, 128.7, 126.7, 83.6, 75.0, 64.5.

FTIR ν_{max} (ATR, film)/cm^{-1} 3290, 1493, 1453, 2349, 1493, 1453, 1262, 1191, 1019, 946, 723.

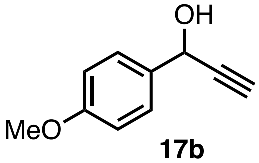

17b

Alkyne **17b** was prepared using *p*-anisaldehyde (504 mg, 450 μL, 3.70 mmol), trimethylsilylacetylene (403 mg, 569 μL, 4.11 mmol), *n*-BuLi (1.6 M in hexane, 2.45 mL, 3.92 mmol) and THF (11 mL) according to General Procedure D. The crude product was purified by flash column chromatography (SiO_2; EtOAc:hexanes = 1:9 → 1:5) to afford pure **17b** (312 mg, 47% yield) as yellow oil. Spectral data are in agreement with the values reported in the literature.[4]

R_f = 0.43 (EtOAc:hexanes = 1:3)

TLC Visualization: UV active; stains with $KMnO_4$ solution.

^{1}H NMR (400 MHz; $CDCl_3$) δ: 7.46 (2H, app d, *J* = 8.4 Hz), 6.90 (2H, app d, *J* = 8.8 Hz), 5.40 (1H, d, *J* = 2.2 Hz), 3.80 (3H, s) 2.66 (1H, d, *J* = 2.2 Hz), 2.56 (1H, br s).

^{13}C{^{1}H} NMR (100 MHz; $CDCl_3$) δ: 159.9, 132.5, 128.2, 114.1, 83.9, 74.7, 64.1, 55.5.

FTIR ν_{max} (ATR, film)/cm^{-1} 3394 (br), 3285, 1610, 1510, 1304, 1243, 1172, 1025, 947, 831, 810.

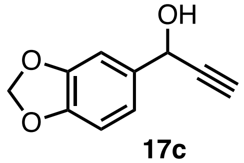

17c

Alkyne **17c** was prepared from piperonal (400 mg, 2.7 mmol), trimethylsilylacetylene (314 mg, 443 µL, 3.2 mmol), *n*-BuLi (1.6 M in hexane, 1.8 mL, 2.9 mmol) and THF (11 mL) according to General Procedure D. The crude product was purified by flash column chromatography (SiO_2; EtOAc:hexanes = 1:4) to afford pure **17c** (407 mg, 86% yield) as colorless oil. Spectral data are in agreement with the values reported in the literature.[5]

R_f = 0.52 (EtOAc:hexanes = 1:4)

TLC Visualization: UV active; stains rapidly with $KMnO_4$ solution.

^{1}H NMR (400 MHz; $CDCl_3$) δ: 7.01 (1H, d, *J* = 1.8 Hz), 6.96 (1H, dd, *J* = 8.1, 1.9 Hz), 6.76 (1H, d, *J* = 8.0 Hz), 5.92 (2H, s), 5.31 (1H, s), 3.07 (1H, s), 2.66 (1H, d, *J* = 2.2 Hz).

^{13}C{^{1}H} NMR (100 MHz; $CDCl_3$) δ: 147.8, 147.7, 134.2, 120.4, 108.2, 107.4, 101.2, 83.7, 74.8, 64.0.

FTIR ν_{max} (ATR, film)/cm^{-1} 3285, 1502, 1486, 1441, 1236, 1096, 1034.

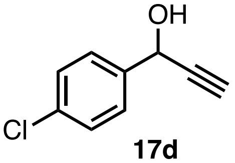

17d

Alkyne **17d** was prepared from *p*-chlorobenzaldehyde (250 mg, 1.78 mmol), trimethylsilylacetylene (199 mg, 280 µL, 2.02 mmol), *n*-BuLi (1.6 M in hexane, 1.20 mL, 1.92 mmol) and THF (7 mL) according to General Procedure D. The crude product was purified by flash column chromatography (SiO_2; EtOAc:hexanes = 1:19 → 1:9 → 1:5) to afford pure **17d** (138 mg, 47% yield) as yellow oil. Spectral data are in agreement with the values reported in the literature.[4]

R_f = 0.52 (EtOAc:hexanes = 1:4)

TLC Visualization: UV active; stains with $KMnO_4$ solution.

^{1}H NMR (400 MHz; $CDCl_3$) δ: 7.44 (2H, d, *J* = 8.5 Hz), 7.33 (2H, d, *J* = 8.4 Hz), 5.39 (1H, br s), 3.11 (1H, app d, *J* = 4.4 Hz), 2.67 (1H, dd, *J* = 2.2, 0.6 Hz).

$^{13}C\{^1H\}$ NMR (100 MHz; $CDCl_3$) δ: 138.5, 134.4, 128.9, 128.1, 83.2, 75.3, 63.7.

FTIR ν_{max} (ATR, film)/cm^{-1} 3294, 2886, 2120, 1597, 1490, 1405, 1261, 1191, 1090, 1013, 944.

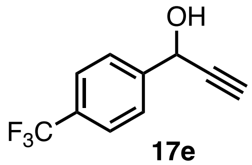

17e

Alkyne **17e** was prepared from 4-(trifluoromethyl)benzaldehyde (500 mg, 384 µL, 2.9 mmol), trimethylsilylacetylene (313 mg, 442 µL, 3.2 mmol), *n*-BuLi (1.6 M in hexane, 1.9 mL, 3.04 mmol) and THF (10 mL) according to General Procedure D. The crude product was purified by flash column chromatography (SiO_2; EtOAc:hexanes = 1:9) to afford pure **17e** (410 mg, 71% yield) as orange oil. Spectral data are in agreement with the values reported in the literature.[6]

R_f = 0.36 (EtOAc:hexanes = 1:7)

TLC Visualization: UV active; stains with $KMnO_4$ solution.

^{1}H NMR (400 MHz; $CDCl_3$) δ: 7.67 (2H, d, *J* = 8.8 Hz), 7.64 (2H, d, *J* = 8.8 Hz), 5.52 (1H, d, *J* = 2.3 Hz), 2.70 (1H, d, *J* = 2.2 Hz), 2.58 (1H, s).

$^{13}C\{^{1}H\}$ NMR (100 MHz; $CDCl_3$) δ: 143.9, 130.7 (q, $^{2}J_{C\text{-}F}$ = 32.4 Hz), 127.0, 125.6 (q, $^{3}J_{C\text{-}F}$ = 3.8 Hz), 124.1 (q, $^{1}J_{C\text{-}F}$ = 272 Hz), 82.9, 75.5, 63.6.

FTIR ν_{max} (ATR, film)/cm^{-1} 3307, 1621, 1416, 1322, 1163, 1121, 1109, 1066, 1016, 946.

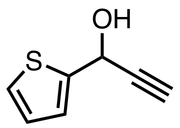

17f

Alkyne **17f** was prepared from thiophene-2-carboxaldehyde (500 mg, 416 µL, 4.46 mmol), trimethylsilylacetylene (486 mg, 686 µL, 4.95 mmol), *n*-BuLi (1.6 M in hexane, 2.96 mL, 4.73 mmol) and THF (13 mL) according to General Procedure D. The crude product was purified by flash column chromatography (SiO_2; EtOAc:hexanes = 1:19 → 1:5 → 1:2) to afford pure **17f** (527 mg, 86% yield) as orange oil. Spectral data are in agreement with the values reported in the literature.[7]

R_f = 0.32 (EtOAc:hexanes = 1:5)

TLC Visualization: UV active; stains with $KMnO_4$ solution.

^{1}H NMR (400 MHz; $CDCl_3$) δ: 7.31-7.30 (1H, m), 7.18 (1H, app d, *J* = 3.5 Hz), 6.98 (1H, dd, *J* = 5.1, 3.5 Hz), 5.63 (1H, dd, *J* = 6.5, 1.8 Hz), 3.05 (1H, br s), 2.68 (1H, d, *J* = 2.2 Hz).

$^{13}C\{^{1}H\}$ NMR (100 MHz; $CDCl_3$) δ: 143.9, 126.8, 126.3, 125.9, 82.9, 74.4, 60.0.

FTIR ν_{max} (ATR, film)/cm^{-1} 3286, 1261, 1229, 1009, 917.

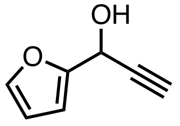

17g

Alkyne **17g** was prepared from furfural (300 mg, 259 µL, 3.1 mmol), trimethylsilylacetylene (367 mg, 518 µL, 3.7 mmol), *n*-BuLi (1.6 M in hexane, 1.8 mL, 2.9 mmol) and THF (8 mL) according to General Procedure D. The crude product was purified by flash column chromatography (SiO_2;

EtOAc:hexanes = 1:9→1:7→1:5) to afford pure **17g** (69.2 mg, 18% yield) as yellow oil. Spectral data are in agreement with the values reported in the literature.[8]

R$_f$ = 0.45 (EtOAc:hexanes = 1:5)

TLC Visualization: Weakly UV active; stains rapidly with $KMnO_4$ solution.

^{1}H NMR (400 MHz; $CDCl_3$) δ: 7.41-7.40 (1H, m), 6.46 (1H, dd, *J* = 3.3, 0.7 Hz), 6.35-6.34 (1H, m), 5.45 (1H, d, *J* = 4.0 Hz), 2.81 (1H, d, *J* = 6.2 Hz), 2.62-2.61 (1H, m).

^{13}C{^{1}H} NMR (100 MHz; $CDCl_3$) δ: 152.5, 143.2, 110.5, 108.1, 81.2, 74.2, 58.0.

FTIR ν_{max} (ATR, film)/cm^{-1} 3288, 1501, 1226, 1143, 1006, 963, 906.

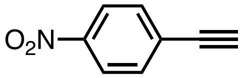

21

An oven-dried round-bottomed flask was cooled under vacuum and refilled with N_2 (×3). 1-Bromo-4-nitrobenzene (500 mg, 2.5 mmol) was dissolved in 10 mL of Et_3N at 23 °C under N_2. CuI (23.6 mg, 0.124 mmol) and $Pd(PPh_3)_2Cl_2$ (43.4, 0.025 mmol) were added sequentially. To the resulting yellow solution, trimethylsilylacetylene (490 mg, 690 μL, 5.0 mmol) was added dropwise over two min. The color of the reaction mixture became brown. Then 5 mL of Et_3N was added along the walls of the reaction flask. The resulting reaction mixture was stirred under N_2 at 23 °C. TLC indicated full consumption of 1-bromo-4-nitrobenzene after 7 h. Et_3N was removed under reduced pressure. The remaining residue was dissolved in CH_2Cl_2 and washed with H_2O. Organic phase was dried over anhydrous Na_2SO_4, filtered and concentrated under reduced pressure. The black solid was dissolved in MeOH (30 mL) followed by addition of K_2CO_3 (413 mg, 3.0 mmol). The resulting reaction mixture was stirred at 23 °C for 1.5 h and quenched with saturated aqueous solution of NH_4Cl (10 mL). The aqueous phase was extracted with EtOAc (3×40 ml). The combined organic layer was dried over anhydrous Na_2SO_4, filtered and concentrated under reduced

pressure. The crude product was purified by flash column chromatography (SiO_2; EtOAc:hexanes = 1:19) to afford pure **21** (280 mg, 77% yield) as a yellow solid. Spectral data are in agreement with the values reported in the literature.[9]

R R_f = 0.47 (EtOAc:hexanes = 1:19)

TLC Visualization: UV active; stains with $KMnO_4$ solution.

^{1}H NMR (400 MHz; $CDCl_3$) δ: 8.20 (2H, d, *J* = 8.9 Hz), 7.64 (2H, d, *J* = 8.9 Hz), 3.36 (1H, s)

^{13}C{^{1}H} NMR (100 MHz; $CDCl_3$) δ: 147.7, 133.1, 129.1, 123.7, 82.5, 81.7.

FTIR ν_{max} (ATR, film)/cm^{-1} 3250, 2105, 1592, 1509, 1342, 1105, 852.

Studies on the Scalability of the Domino Reaction Sequence:

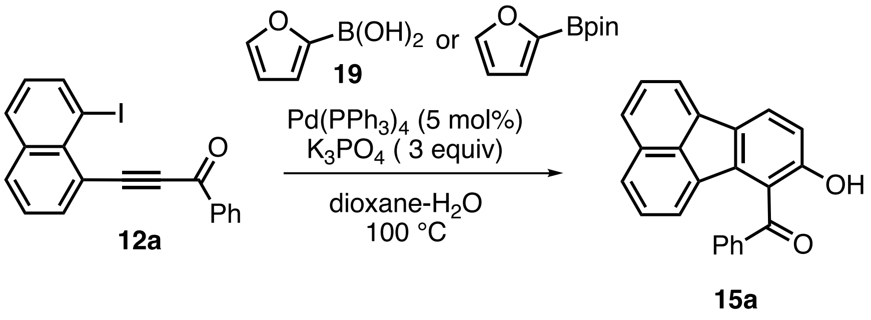

When the domino sequence between **12a** and 2-furylboronic acid (**19**) was tested on 1 mmol scale, a complex mixture of products was obtained without an appreciable amount of the hydroxyfluoranthene **15a**. In order to check the effect of using a boronic ester instead of a boronic acid, furan-2-boronic acid pinacol ester was used as the coupling partner. However, when furan-2-boronic acid pinacol ester was used with substrate **12a** on 1 mmol scale, again a complex mixture was observed to form along with only a small amount of hydroxyfluoranthene product **15a**.

Studies on the Oxidation of Propargylic Alcohols:

Table S1. Oxidation of Alcohols **18a** and **18c**

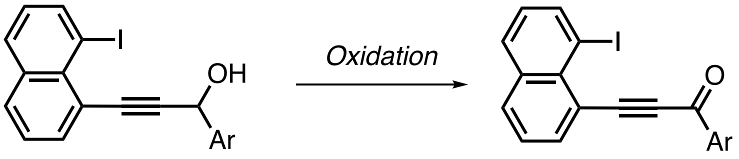

18a or **18c** **12a** or **12c**

**-Ar**	**Oxidation Conditions**	**Yield (%)**
-Ph	PCC, CH_2Cl_2, 23 °C	43
-Ph	DMSO, *i*Pr_2NEt, Pyr·SO_3 CH_2Cl_2, 23 °C	57
-Ph	MnO_2, acetone, 23 °C	91
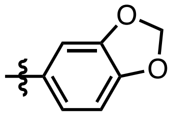	PCC, CH_2Cl_2, 23 °C	51
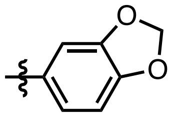	DMP, CH_2Cl_2, 0 to 23 °C	77
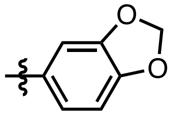	MnO_2, acetone, 23 °C	79

X-Ray Diffraction Analysis of Fluoranthene 15i:

Suitable crystal of **15i** was selected for data collection which was performed on a Bruker diffractometer equipped with a graphite-monochromatic Mo-K_α radiation at 296 K. We used these procedures for our analysis: solved by direct methods; SHELXS-2013;[10] refined by full-matrix least-squares methods; SHELXL-2013;[11] data collection: Bruker APEX2;[12] molecular graphics: MERCURY;[13] solution: WinGX.[14] Details of data collection and crystal structure determinations are given in Tables S2 and S3.

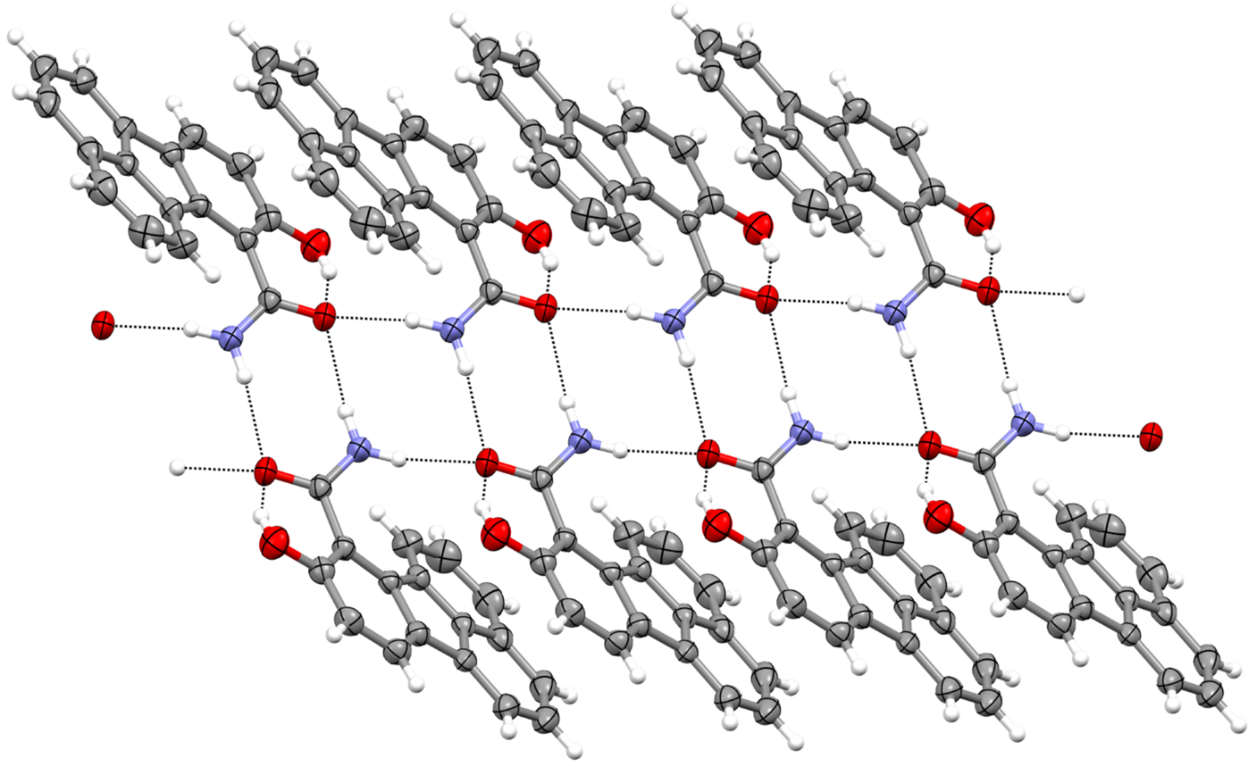

Figure S1. Crystal structure of **15i**, showing the formation of a chain along [010] generated by N-H···O hydrogen bonds.

Table S2. Crystal data and structure refinement parameters for **15i**.

Empirical formula	$C_{17}H_{11}NO_2$
Formula weight	261.27
Crystal system	Monoclinic
Space group	$P2_1/c$
*a (*Å)	15.859 (12)
*b (*Å)	4.783 (3)
*c (*Å)	19.075 (19)
*β (*º)	102.54 (2)°
*V (*Å^3)	1412 (2)
Z	4
D_c (g cm^{-3})	1.229
μ (mm^{-1})	0.08
θ range (º)	1.3-25.5
Measured refls.	43550
Independent refls.	2625
R_{int}	0.070
S	1.10
R1/wR2	0.052/0.141
$\Delta\rho_{max}/\Delta\rho_{min}$ (eÅ^{-3})	0.16/-0.18

Table S3. Hydrogen bond parameters for **15i** (Å, °)

D-H···A	D-H	H···A	D···A	D-H···A
N1—H1A···O2^{i}	0.88 (2)	2.21 (2)	2.965 (3)	144
N1—H1B···O2^{ii}	0.89 (2)	2.10 (2)	2.976 (3)	168
O1—H1C···O2	0.84 (2)	1.84 (2)	2.618 (3)	153

Symmetry codes: (i) x, y+1, z; (ii) −x, −y, −z+1.

References

1. Weimer, M.; Durner, G.; Bats, J. W.; Gobel, M. W. *J. Org. Chem*. **2010**, *75*, 2718-2721.

2. Kawanishi, S.; Oki, S.; Kundu, D.; Akai, S. *Org. Lett.* **2019**, *21*, 2978-2982.

3. Johnston, A. J. S.; McLaughlin, M. G.; Reid, J. P.; Cook, M. J. *Org. Biomol. Chem.* **2013**, *11*, 7662-7666.

4. Xu, C.-F.; Xu, M.; Yang, L.-Q.; Li, C.-Y. *J. Org. Chem.* **2012**, *77*, 3010-3016.

5. Mori, M.; Tonogaki, K.; Nishiguchi, N. *J. Org. Chem.* **2002**, *67*, 224-226.

6. Petrone, D. A.; Isomura, M.; Franzoni, I.; Rössler, S. L.; Carreira, E. M. *J. Am. Chem. Soc.* **2018**, *140*, 4697-4704.

7. Ghazvini, H. J.; Armaghan, M.; Janiak, C.; Balalaie, S.; Müller, T. J. J. *Eur. J. Org. Chem.* **2019**, 7058-7062.

8. Horino, Y.; Murakami, M.; Ishibashi, M.; Lee, J. H.; Watanabe, A.; Matsumoto, R.; Abe, H. *Org. Lett.* **2019**, *21*, 9564-9568.

9. Feng, Y.-S.; Xie, C.-Q.; Qiao, W.-L.; Xu, H.-J. *Org. Lett.* **2013**, *15*, 936-939.

10. Sheldrick, G.M. *Acta Cryst.* **2008**, A64, 112.

11. Sheldrick, G. M. *Acta Cryst.* **2015**, C71, 3.

12. APEX2, Bruker AXS Inc. Madison Wisconsin USA (2013).

13. Macrae, C. F.; Sovago, I.; Cottrell, S. J.; Galek, P. T. A.; McCabe, P.; Pidcock, E.; Platings, M.; Shields, G. P.; Stevens, J. S.; Towler, M.; Wood, P. A. *J. Appl. Cryst.* **2020**, *53*, 226-235.

14. Farrugia, L. J. *J. Appl. Cryst.* **2012**, *45*, 849–854.

^{1}H- and ^{13}C-NMR spectra:

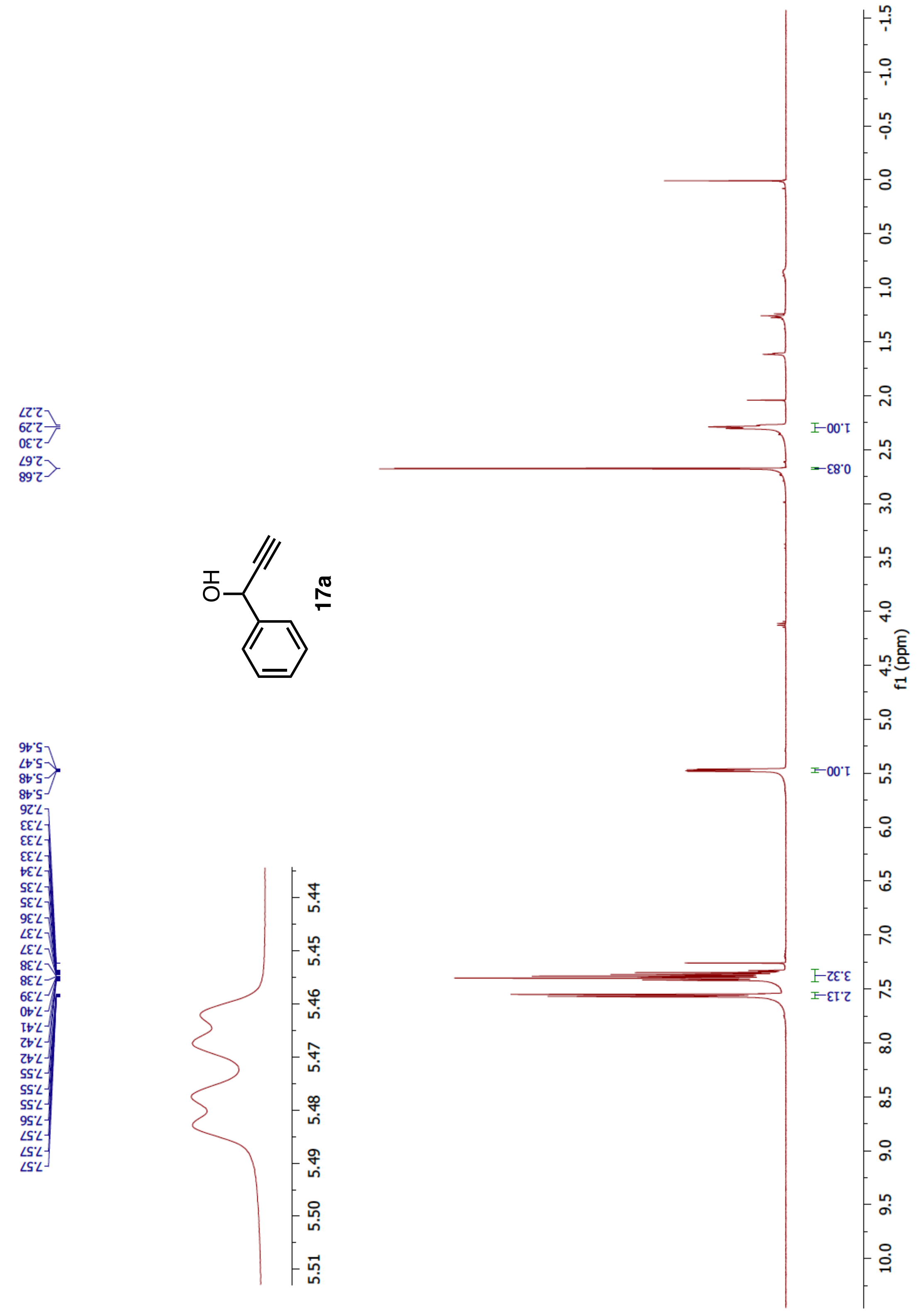

Figure S2. ^{1}H-NMR spectrum of **17a** in $CDCl_3$ (400 MHz).

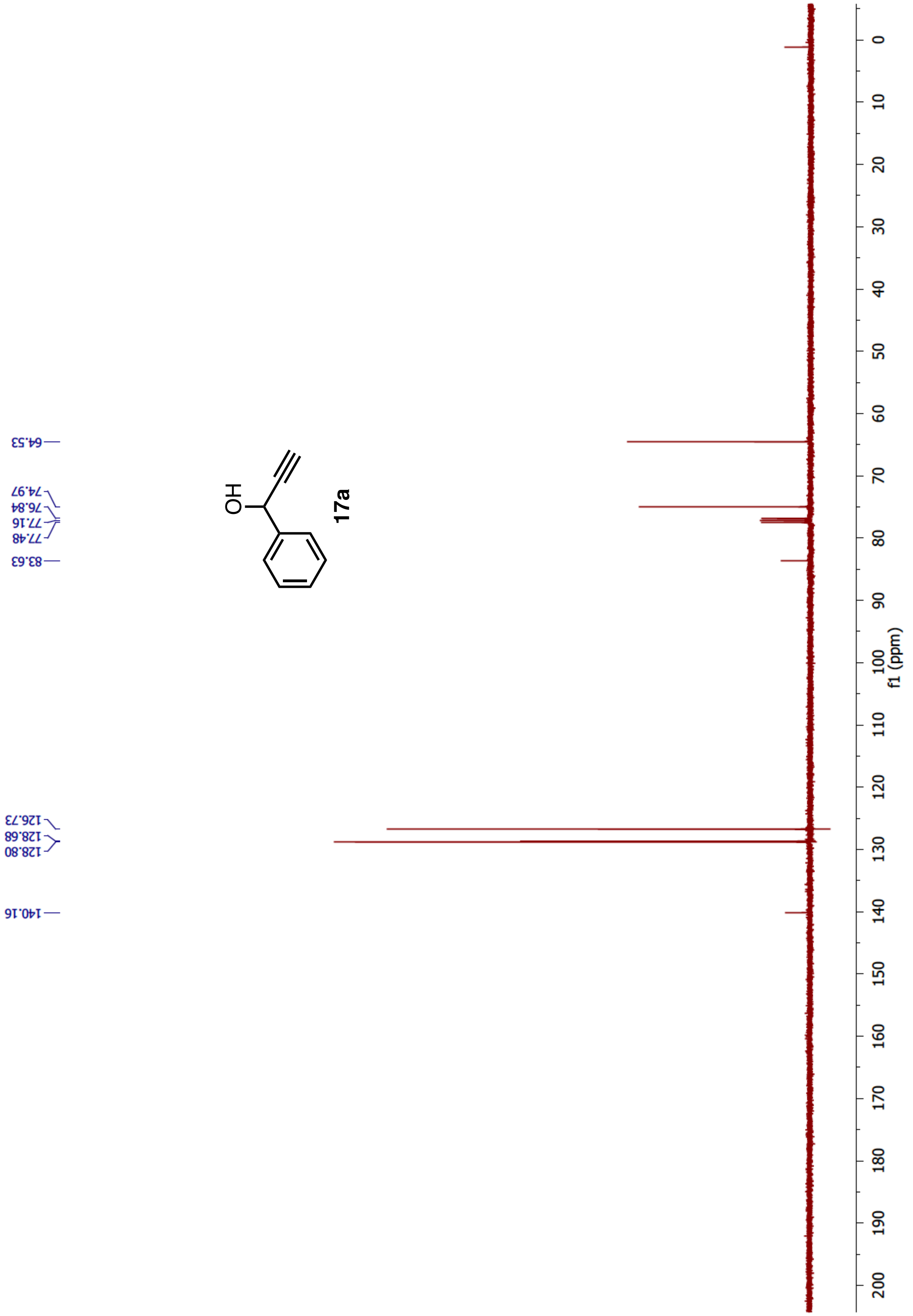

Figure S3. $^{13}C\{^{1}H\}$-NMR spectrum of **17a** in $CDCl_3$ (100 MHz).

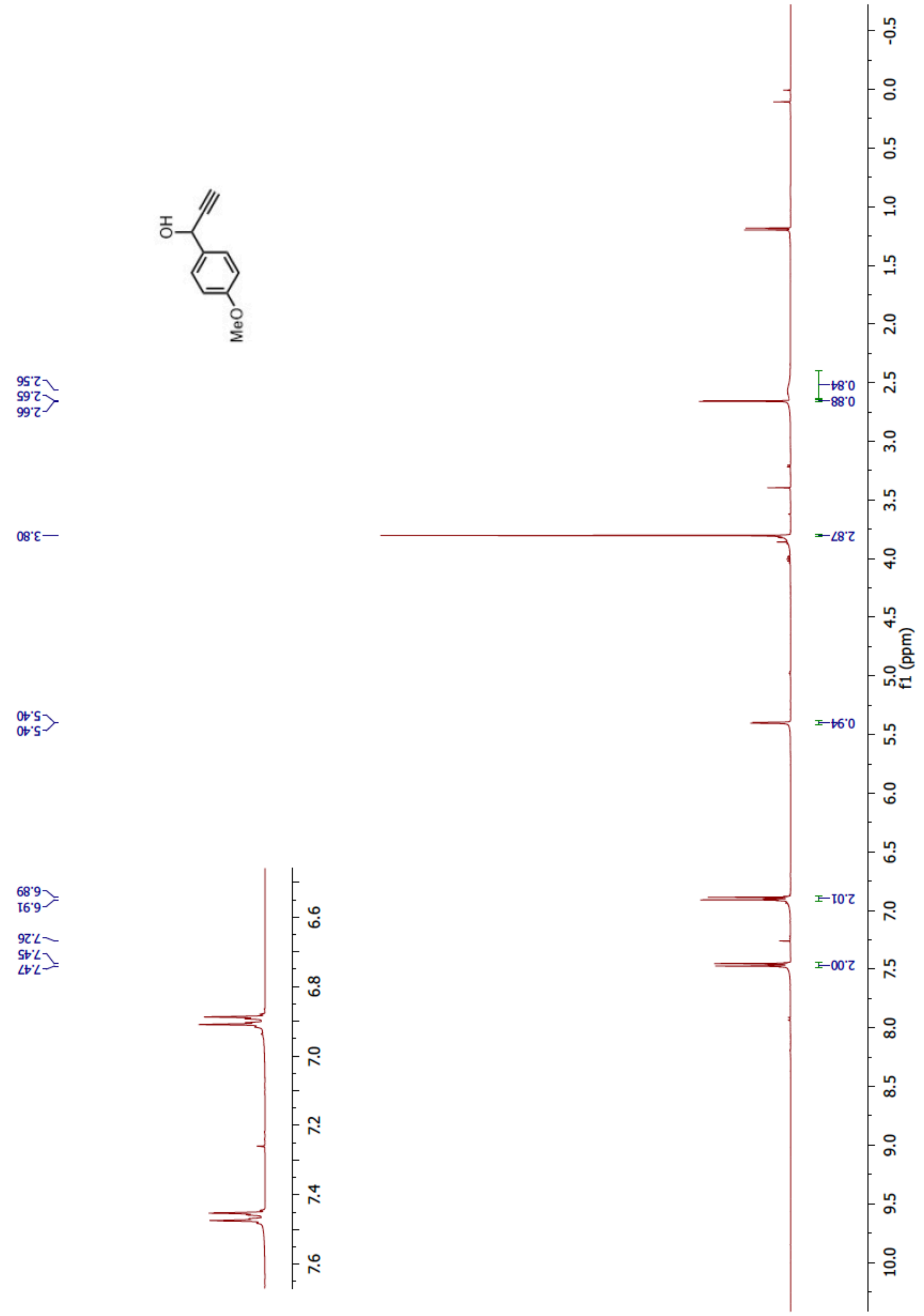

Figure S4. ^{1}H-NMR spectrum of **17b** in $CDCl_3$ (400 MHz).

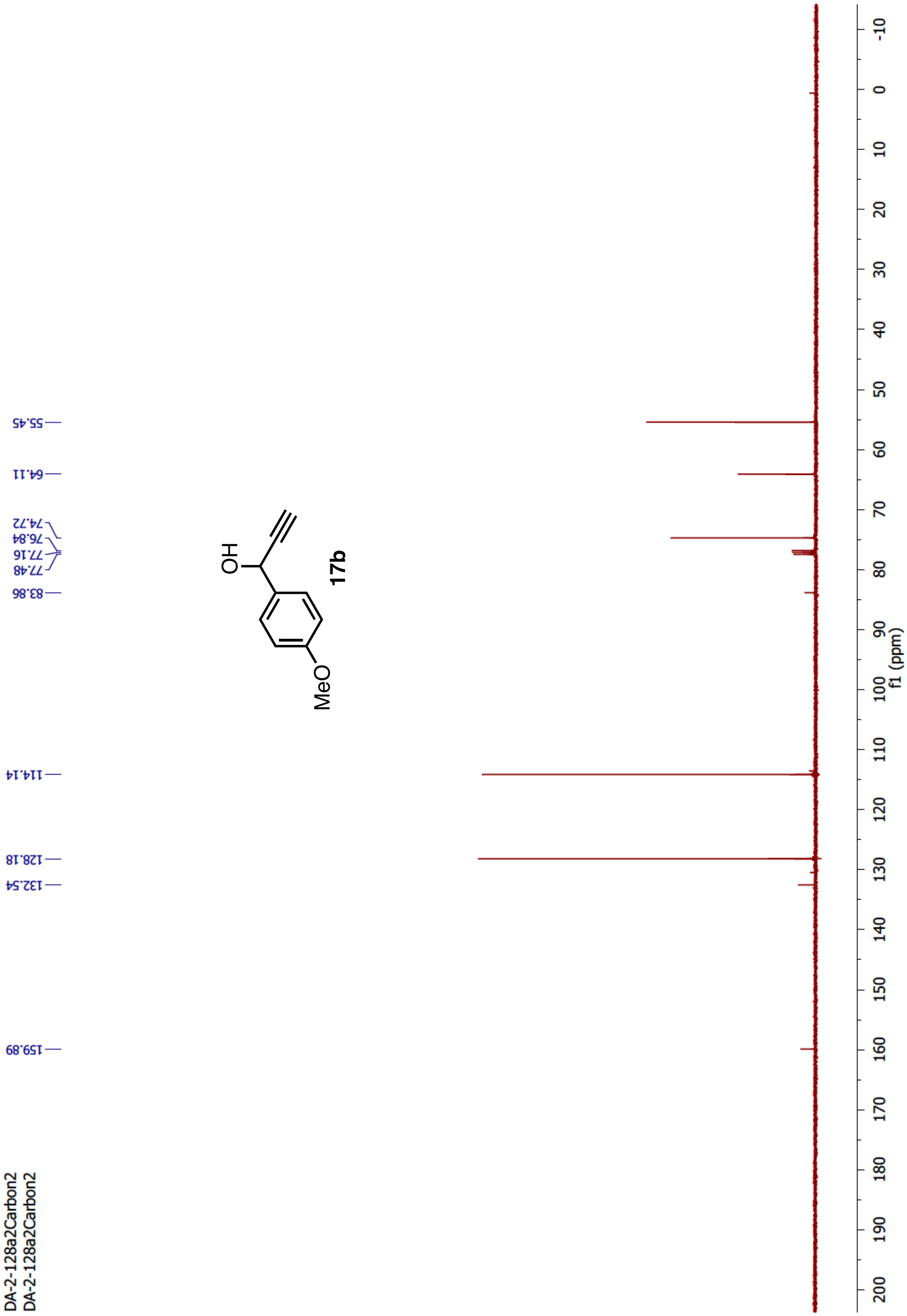

Figure S5. $^{13}C\{^{1}H\}$-NMR spectrum of **17b** in $CDCl_3$ (100 MHz).

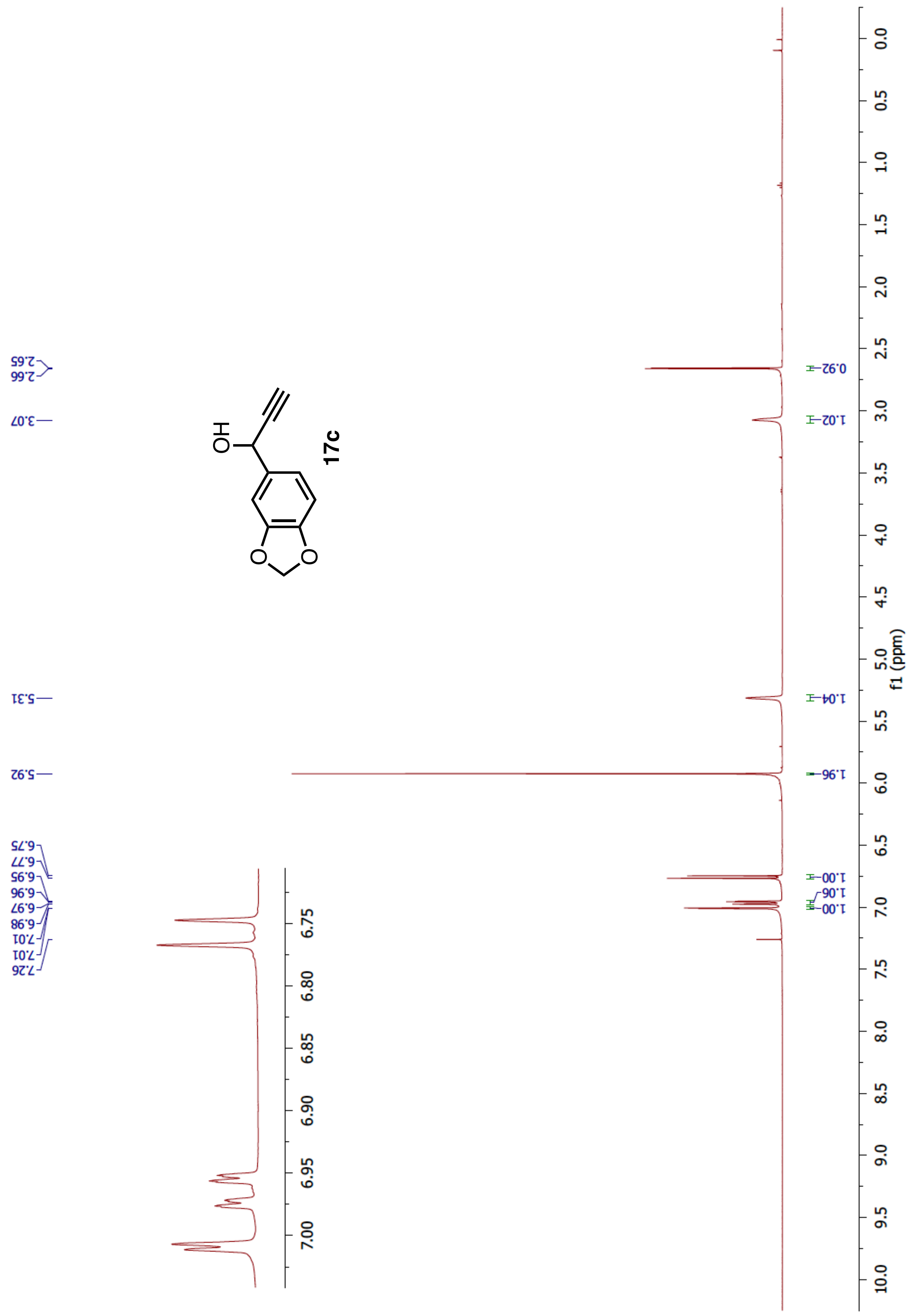

Figure S6. ¹H-NMR spectrum of **17c** in $CDCl_3$ (400 MHz).

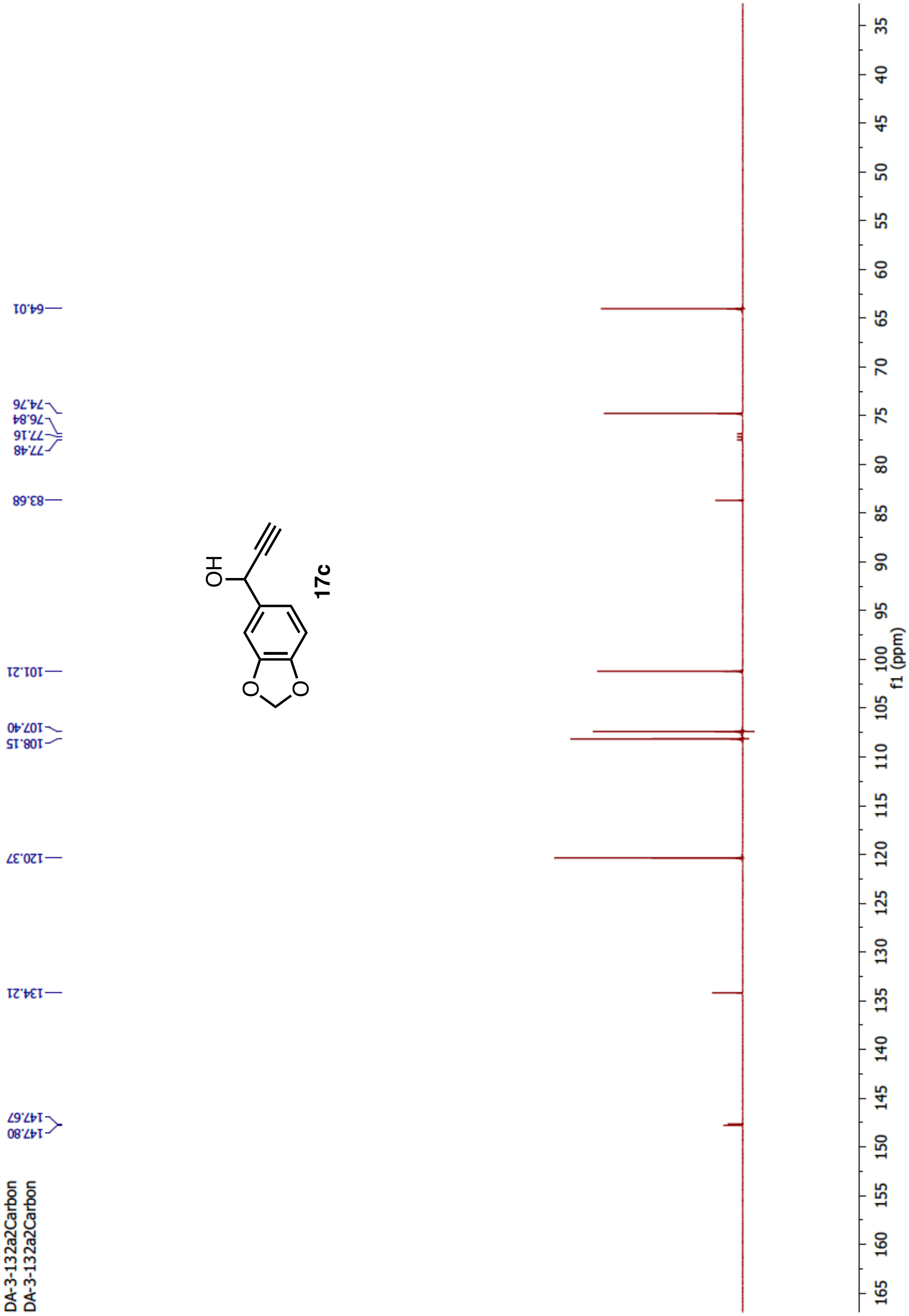

Figure S7. $^{13}C\{^{1}H\}$-NMR spectrum of **17c** in $CDCl_3$ (100 MHz).

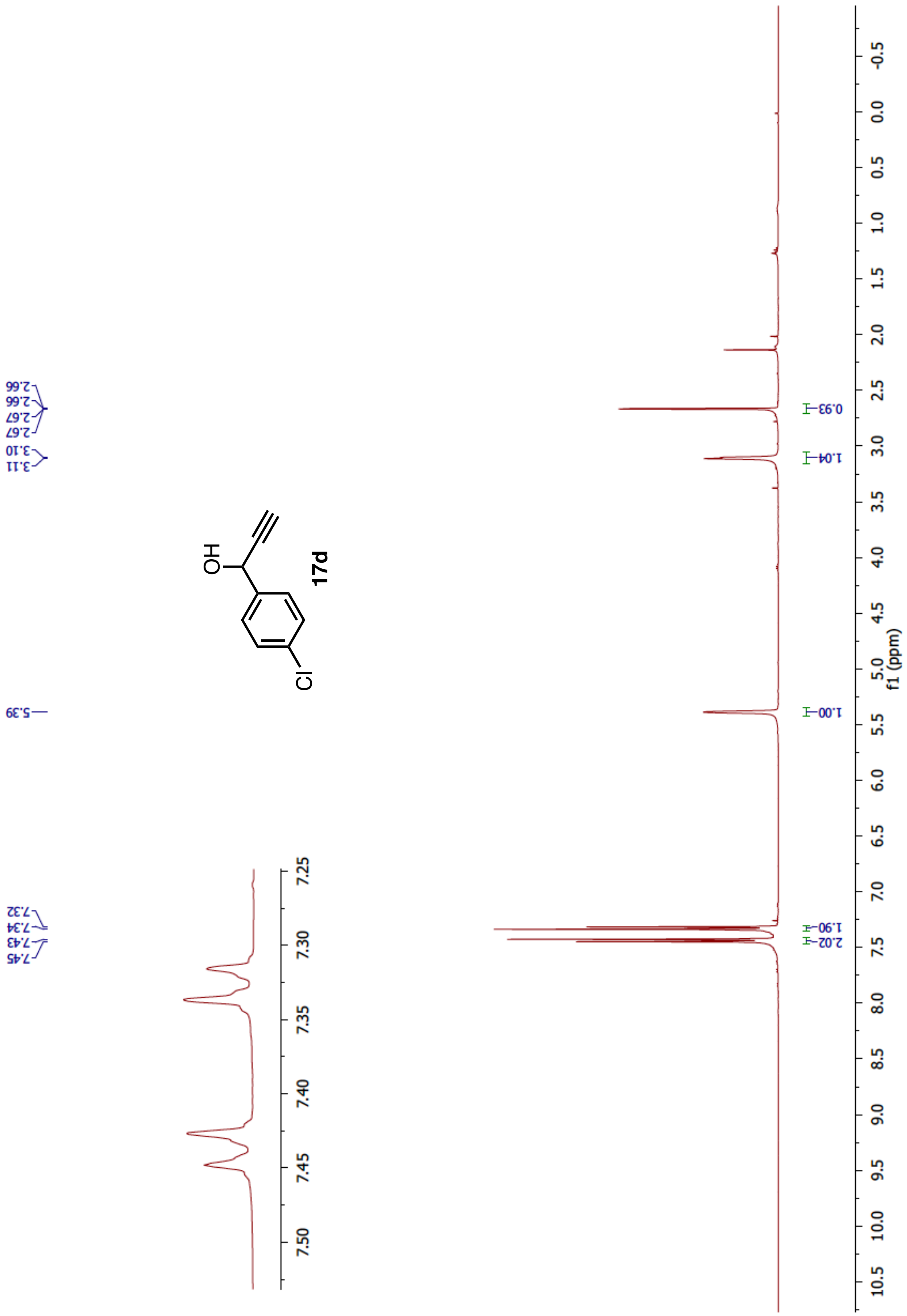

Figure S8. ^{1}H-NMR spectrum of **17d** in $CDCl_3$ (400 MHz).

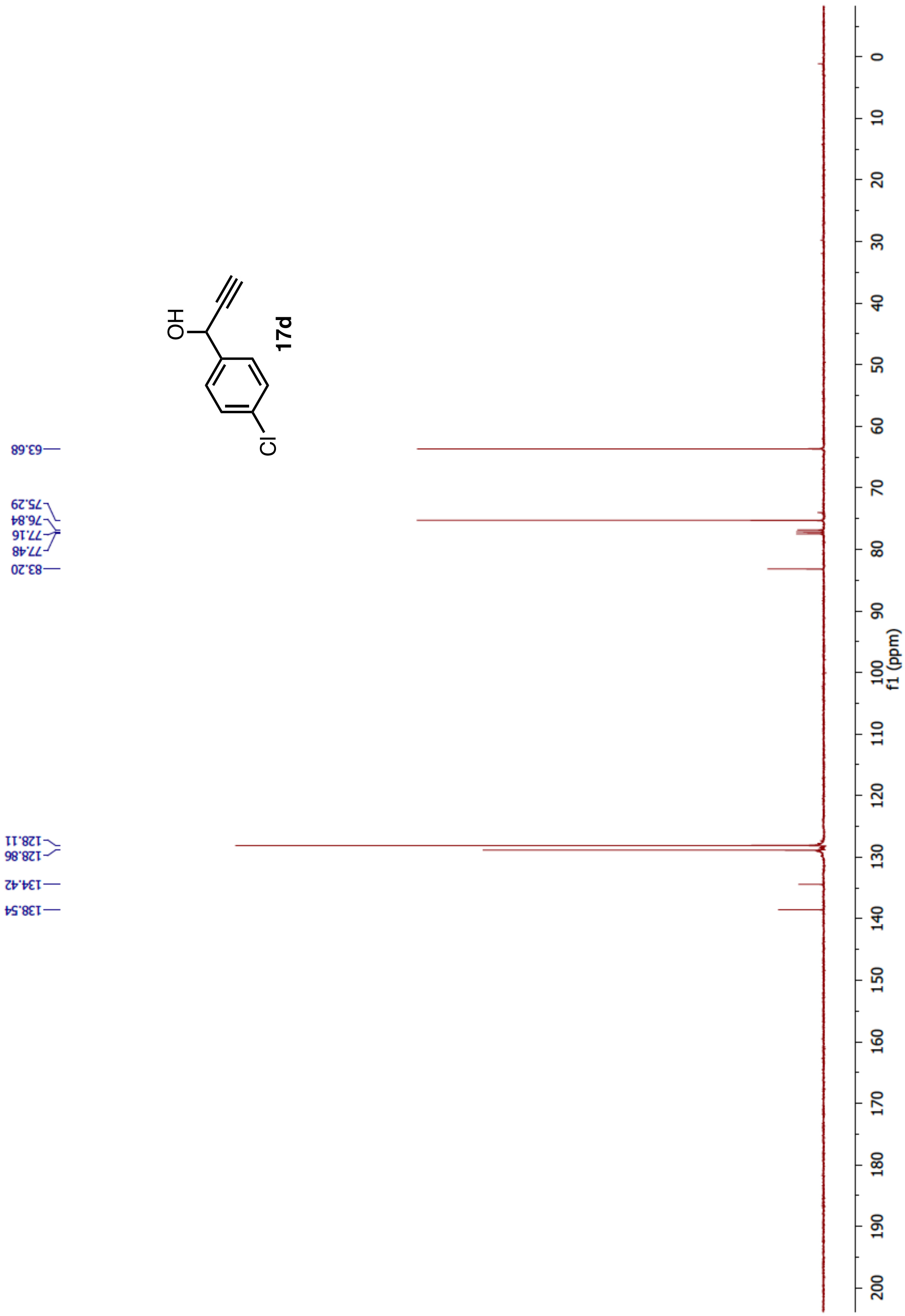

Figure S9. $^{13}C\{^1H\}$-NMR spectrum of **17d** in $CDCl_3$ (100 MHz).

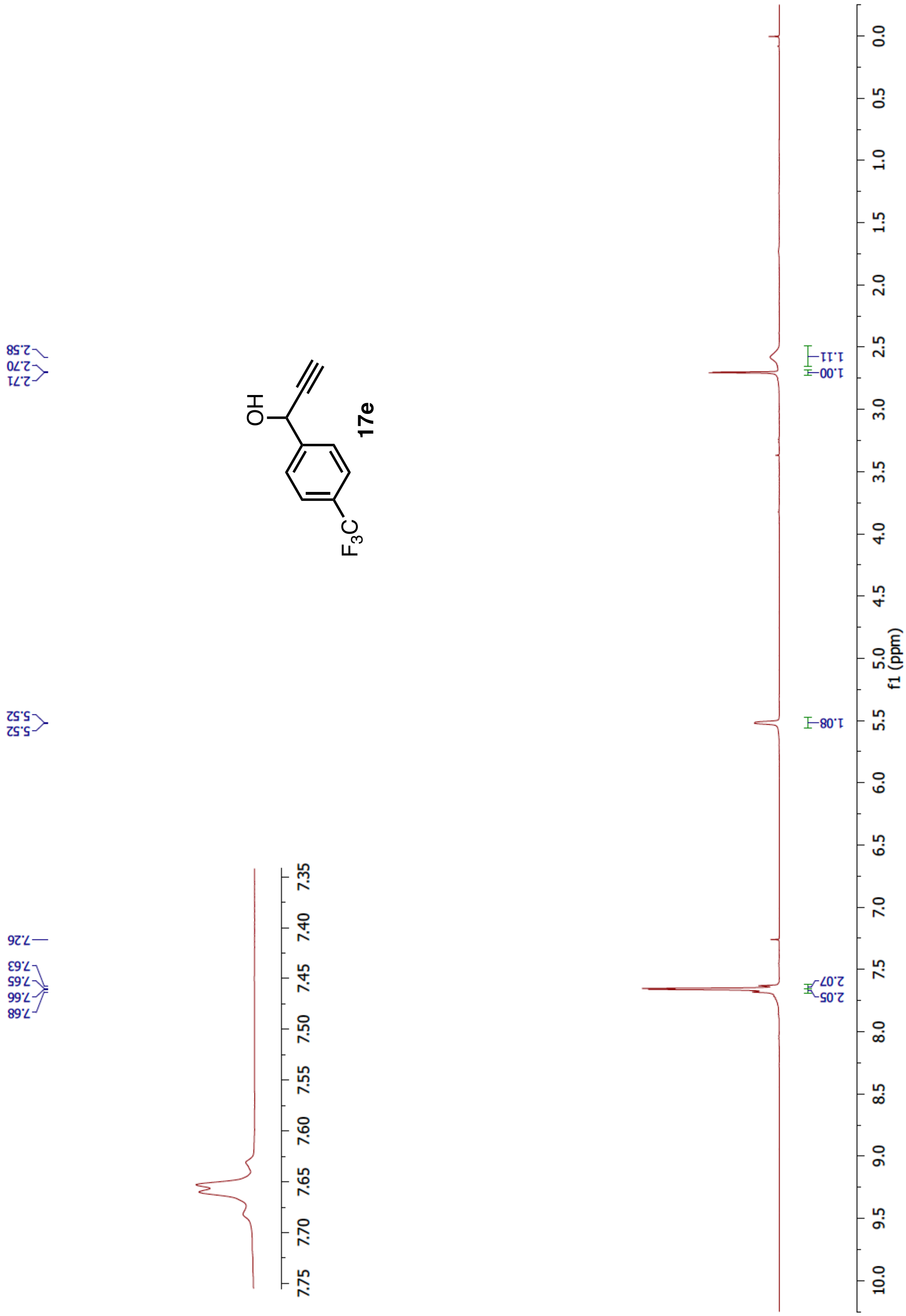

Figure S10. ^{1}H-NMR spectrum of **17e** in $CDCl_3$ (400 MHz).

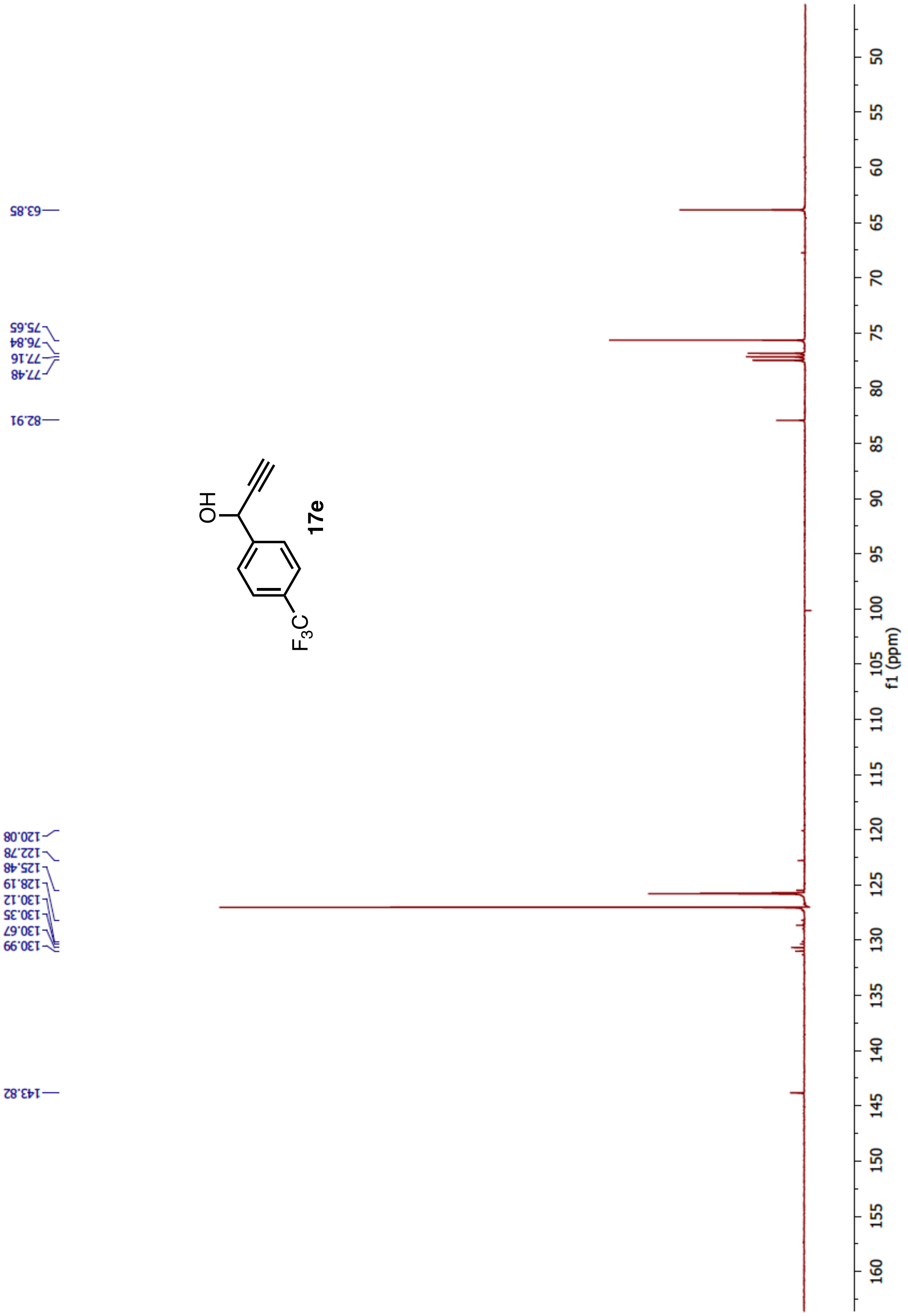

Figure S11. $^{13}C\{^{1}H\}$-NMR spectrum of **17e** in $CDCl_3$ (100 MHz).

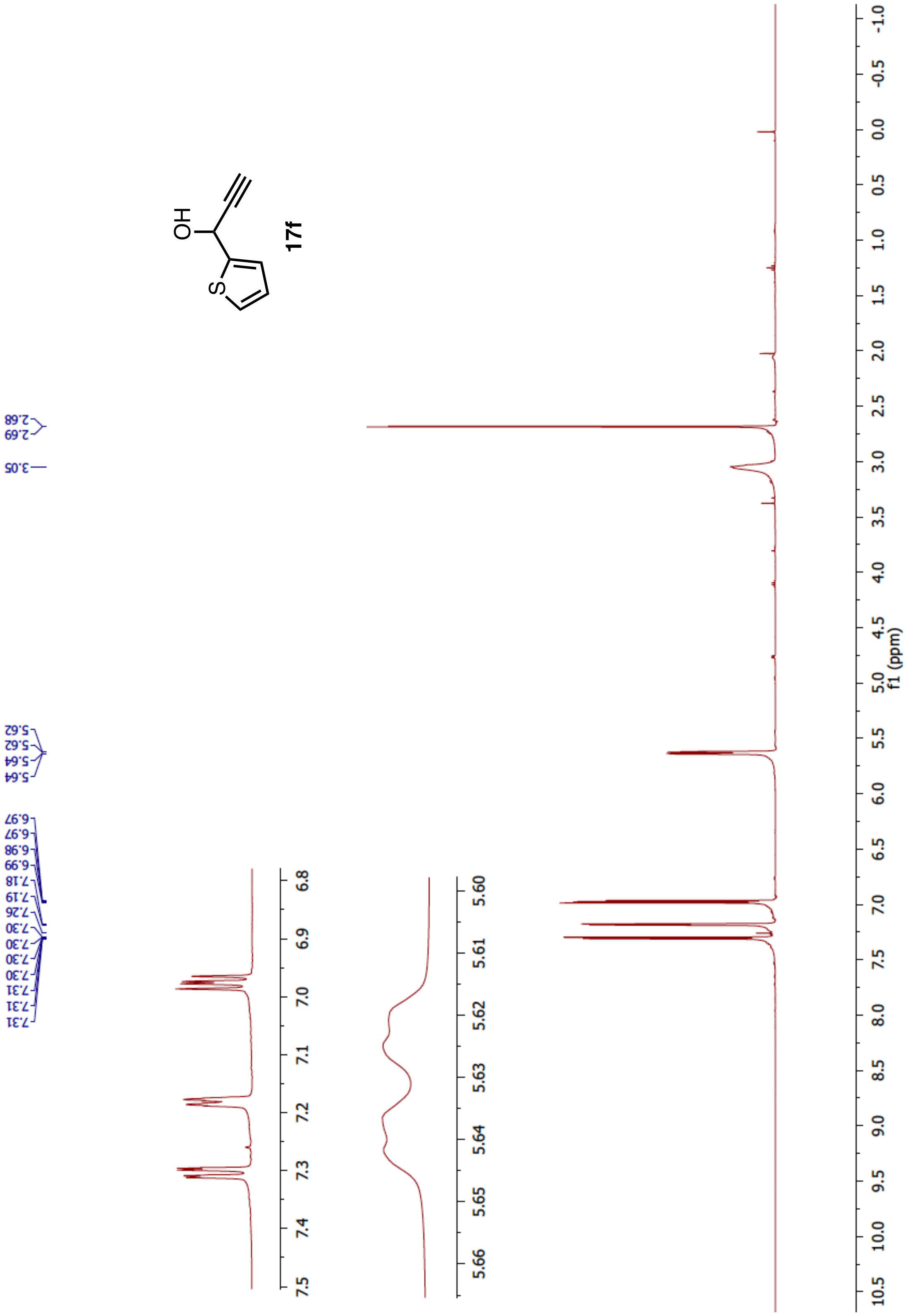

Figure S12. ^{1}H-NMR spectrum of **17f** in $CDCl_3$ (400 MHz).

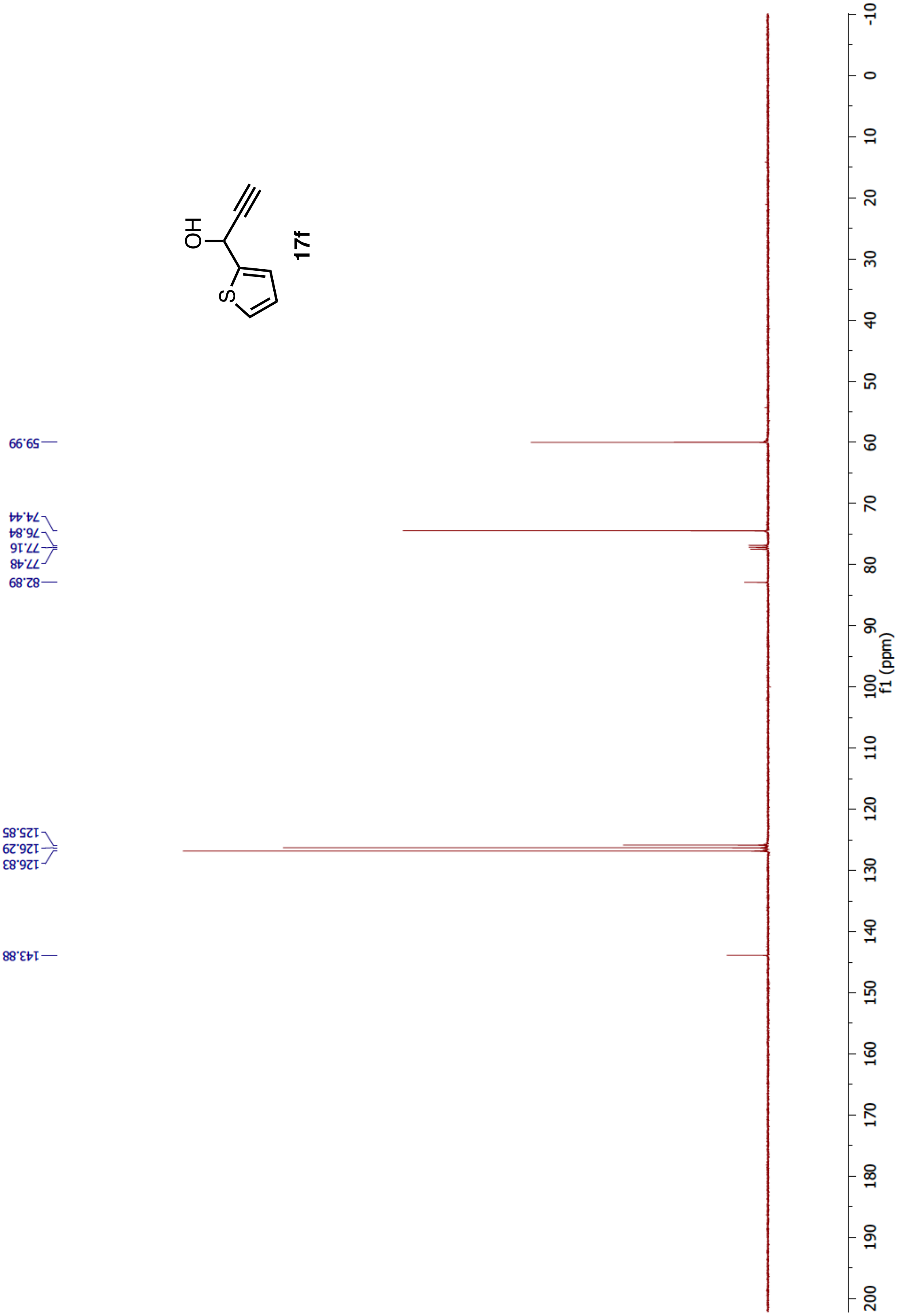

Figure S13. $^{13}C\{^{1}H\}$-NMR spectrum of **17f** in $CDCl_3$ (100 MHz).

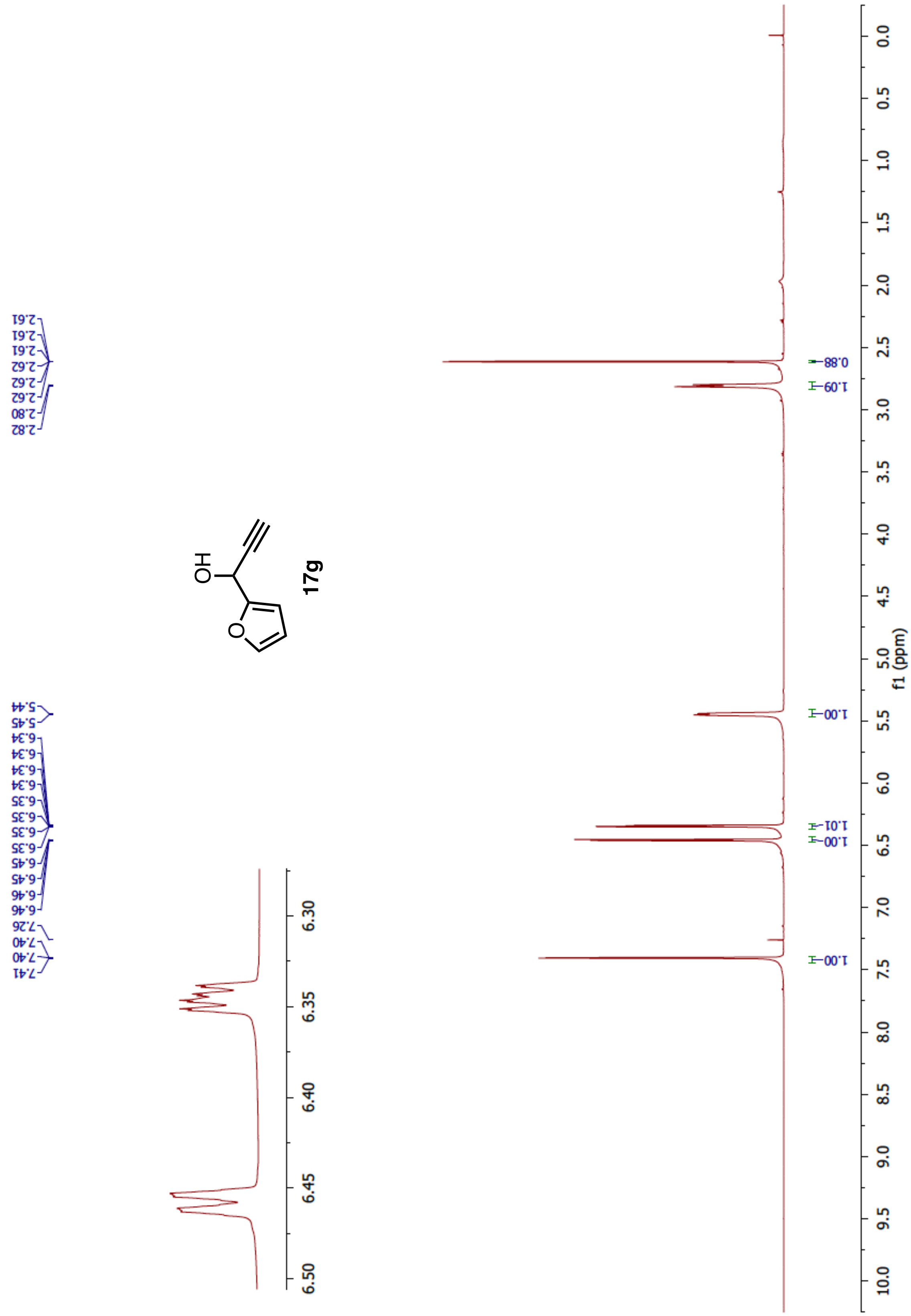

Figure S14. ^{1}H-NMR spectrum of **17g** in $CDCl_3$ (400 MHz).

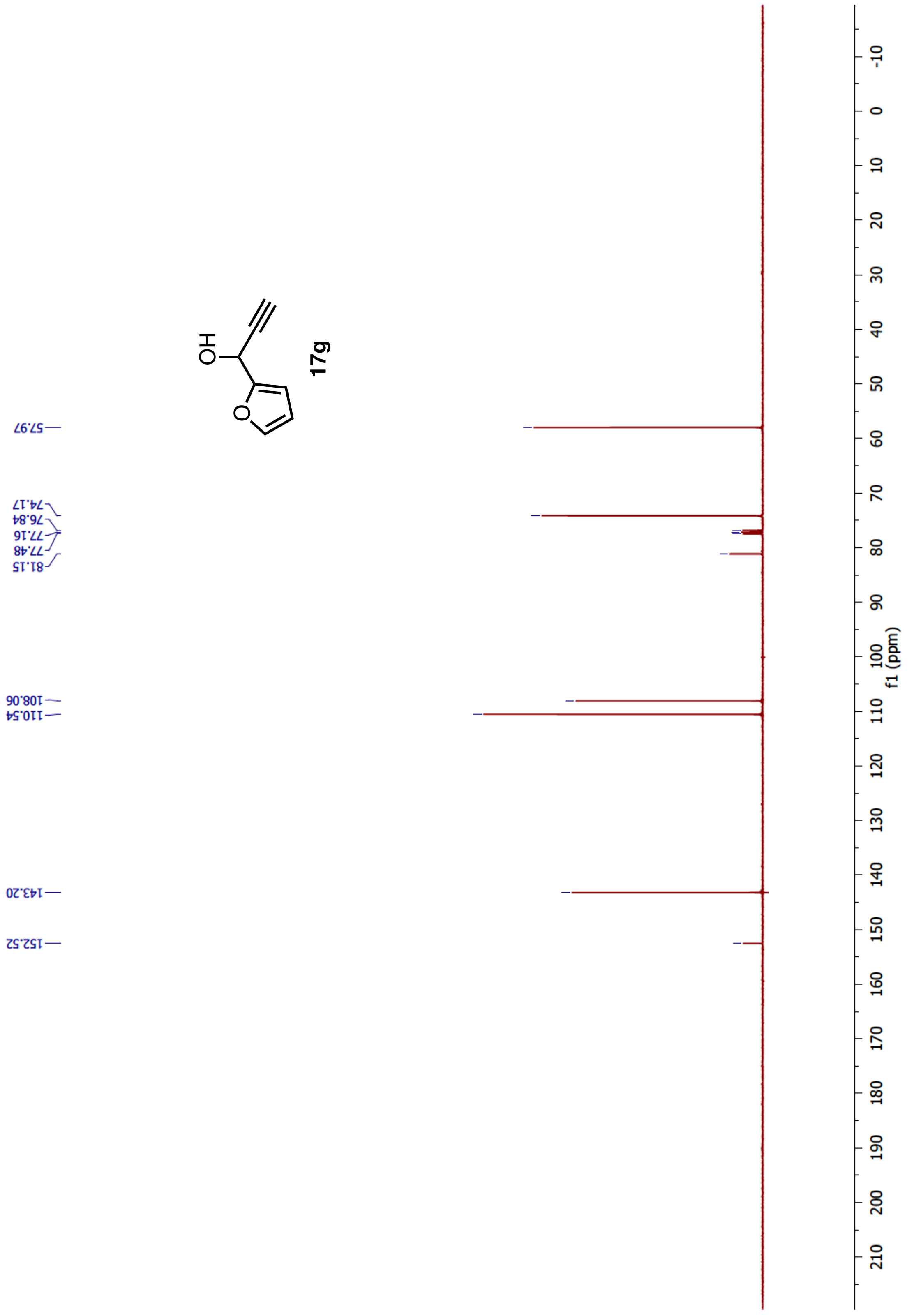

Figure S15. $^{13}C\{^{1}H\}$-NMR spectrum of **17g** in $CDCl_3$ (100 MHz).

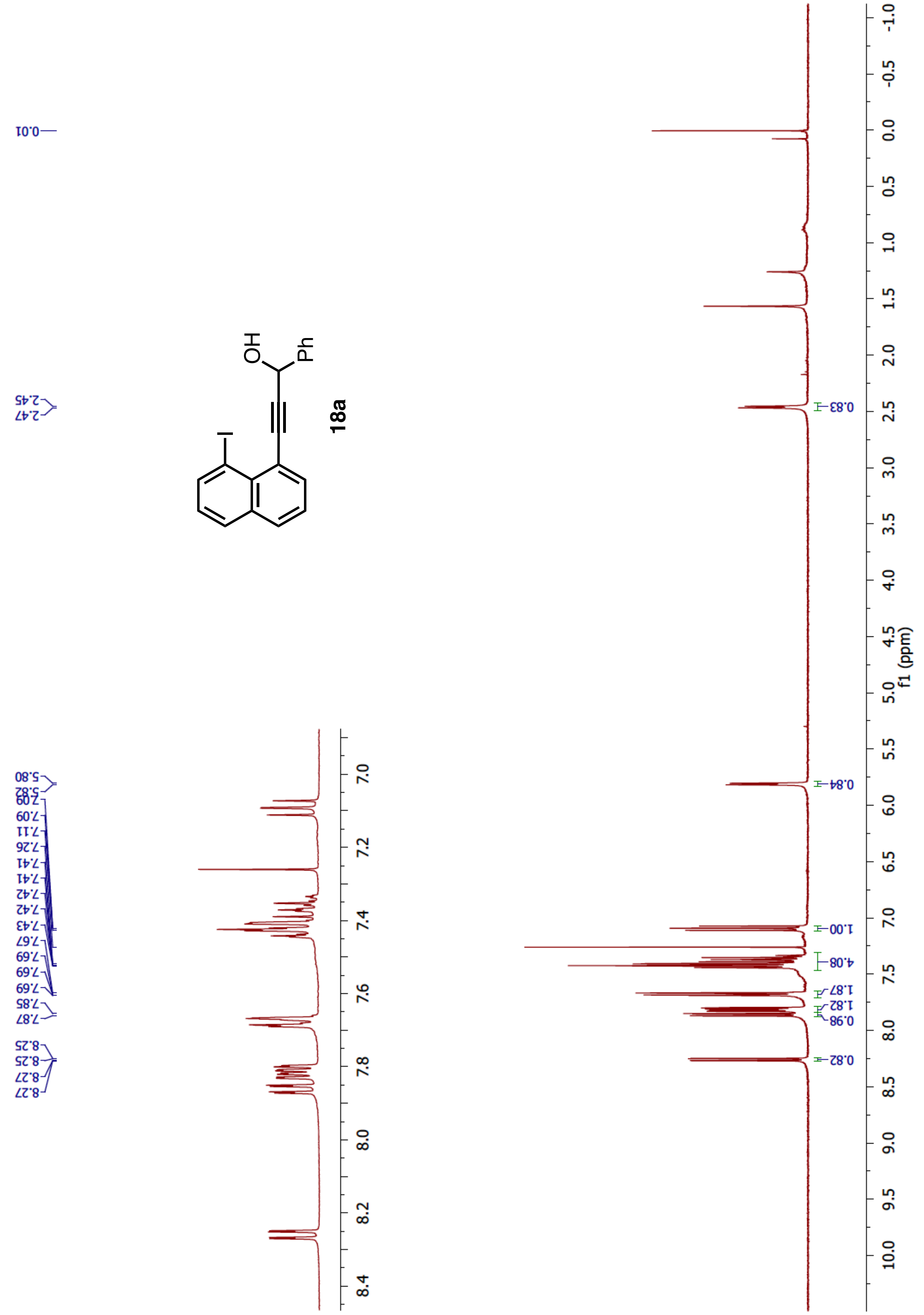

Figure S16. ^{1}H-NMR spectrum of **18a** in $CDCl_3$ (400 MHz).

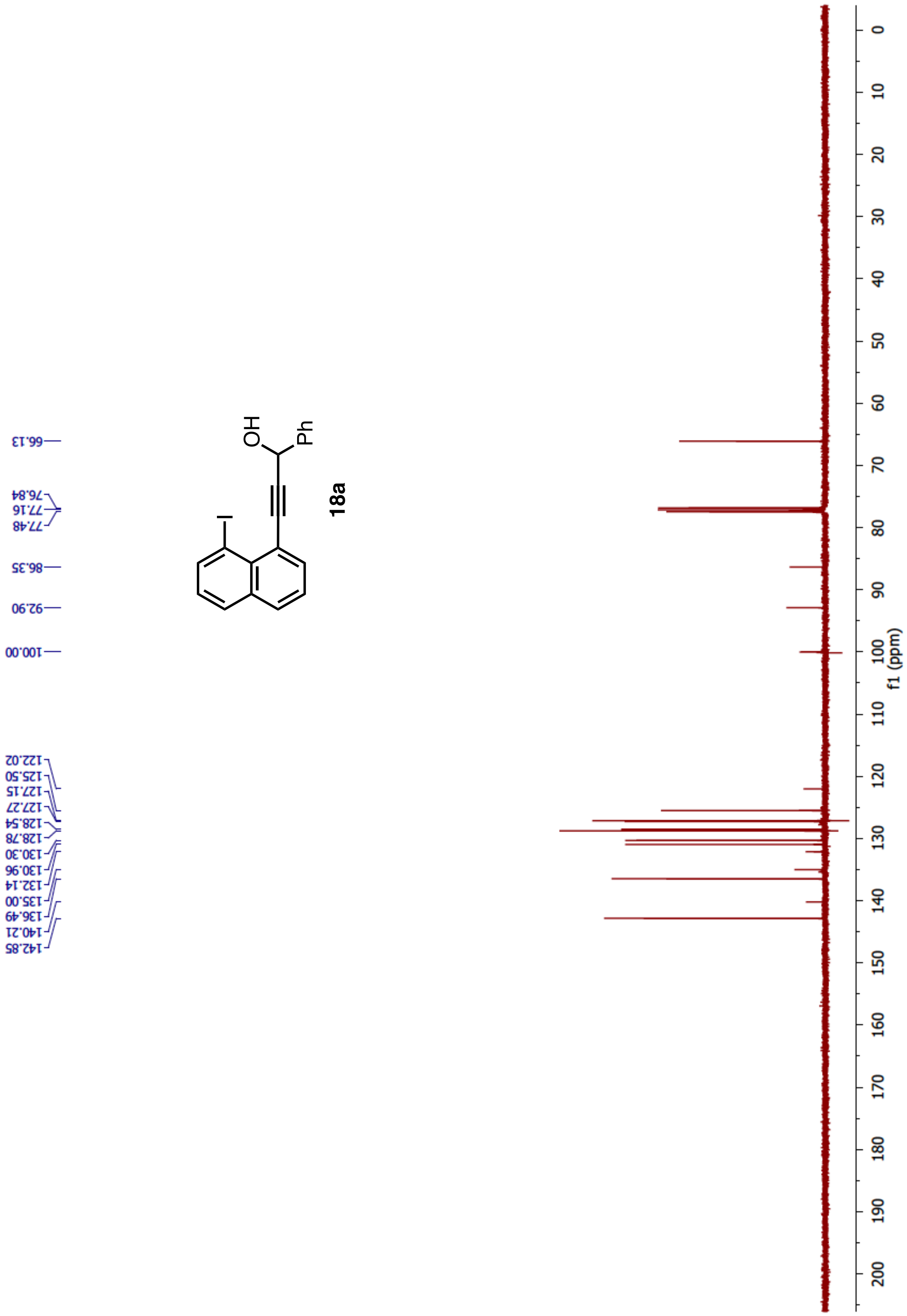

Figure S17. $^{13}C\{^{1}H\}$-NMR spectrum of **18a** in $CDCl_3$ (100 MHz).

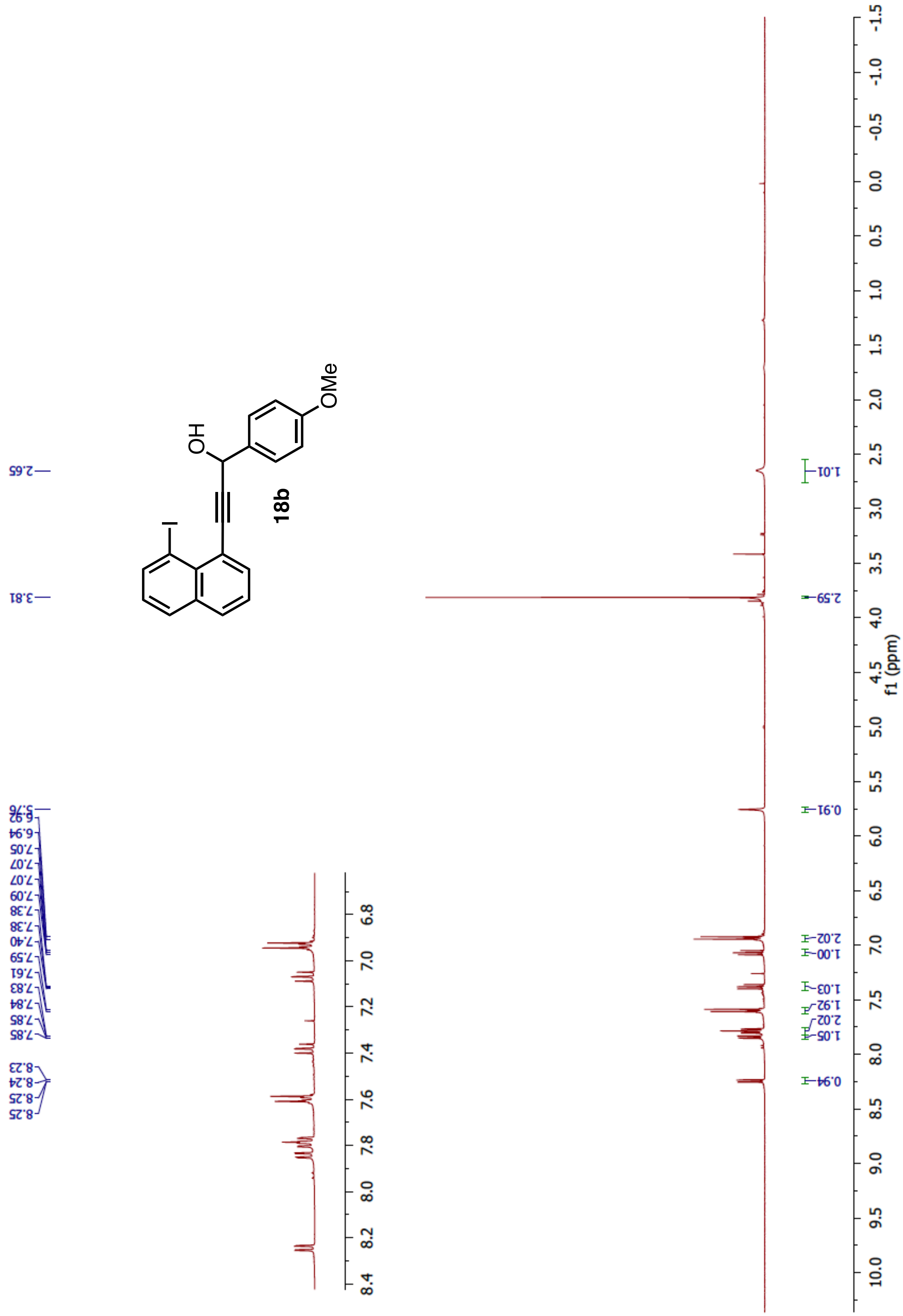

Figure S18. ^{1}H-NMR spectrum of **18b** in $CDCl_3$ (400 MHz).

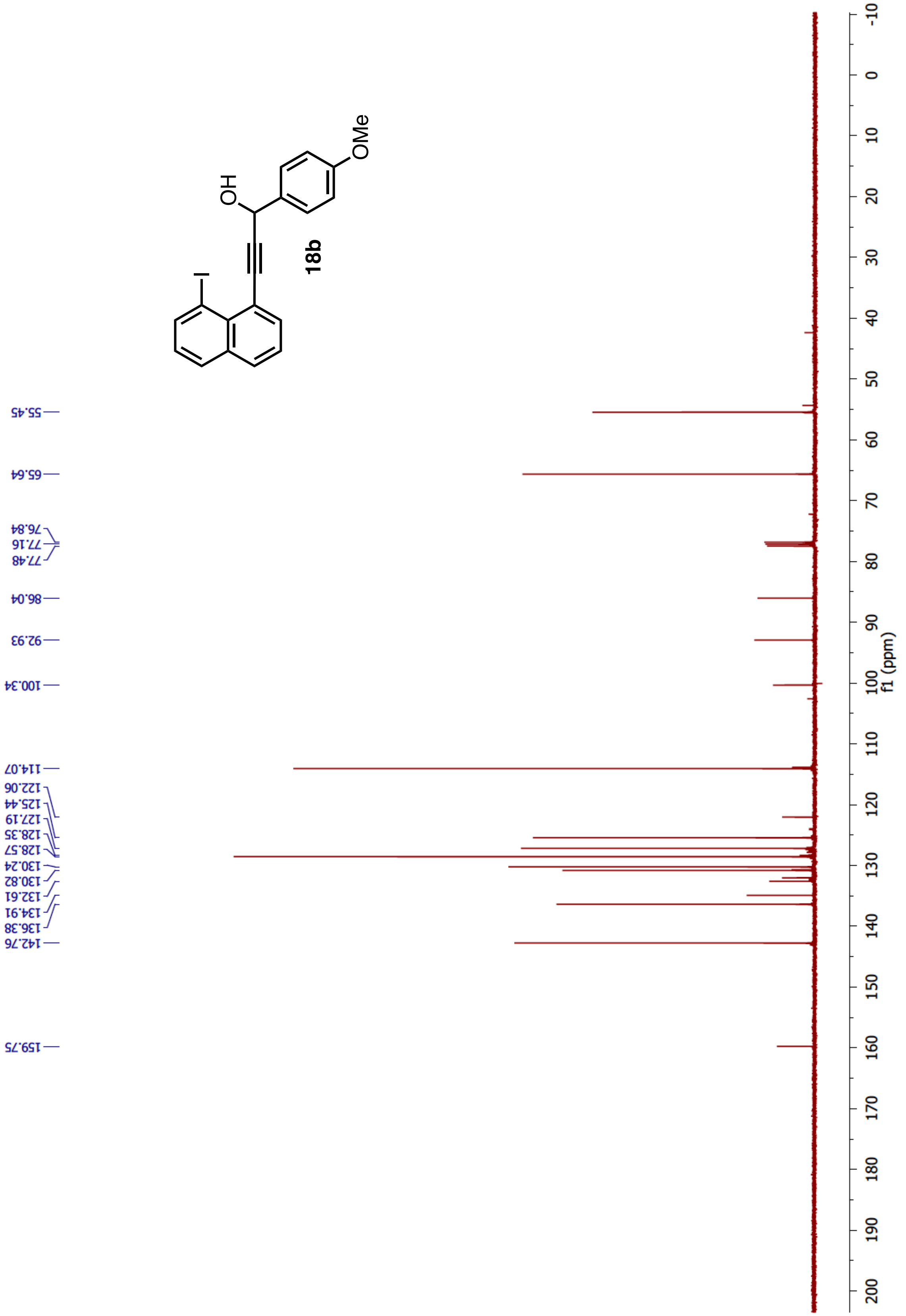

Figure S19. $^{13}C\{^{1}H\}$-NMR spectrum of **18b** in $CDCl_3$ (100 MHz).

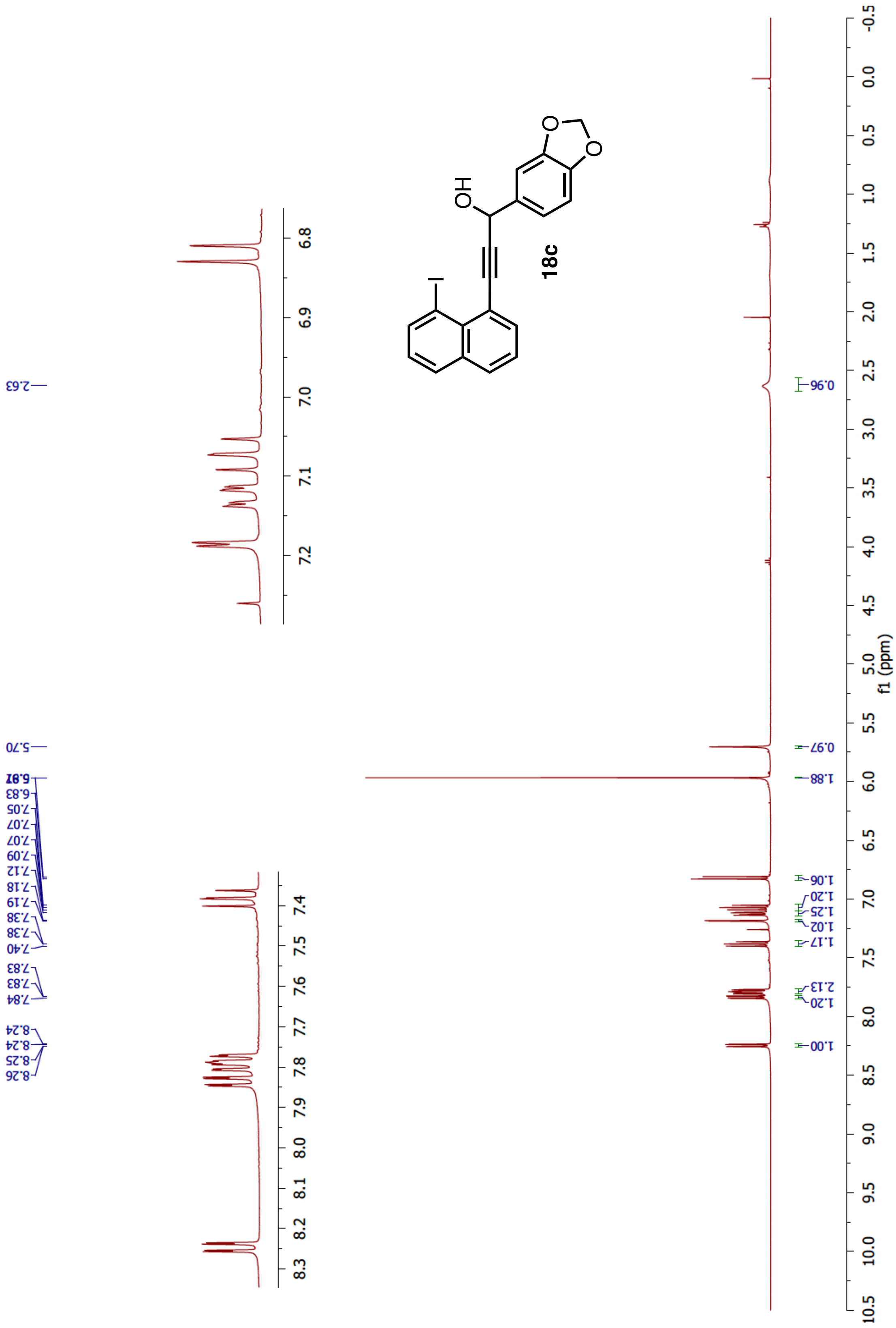

Figure S20. ^{1}H-NMR spectrum of **18c** in $CDCl_3$ (400 MHz).

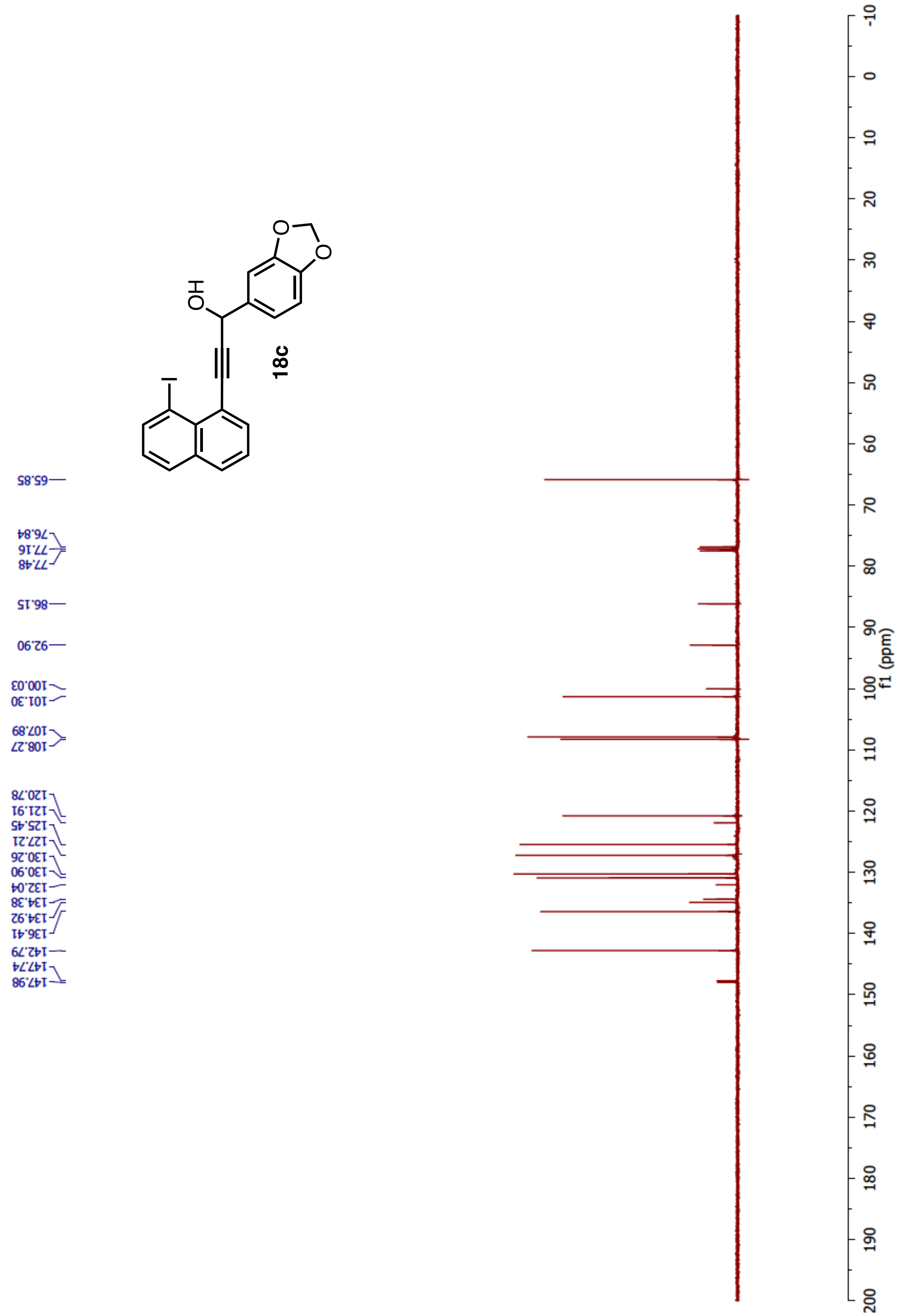

Figure S21. $^{13}C\{^{1}H\}$-NMR spectrum of **18c** in $CDCl_3$ (100 MHz).

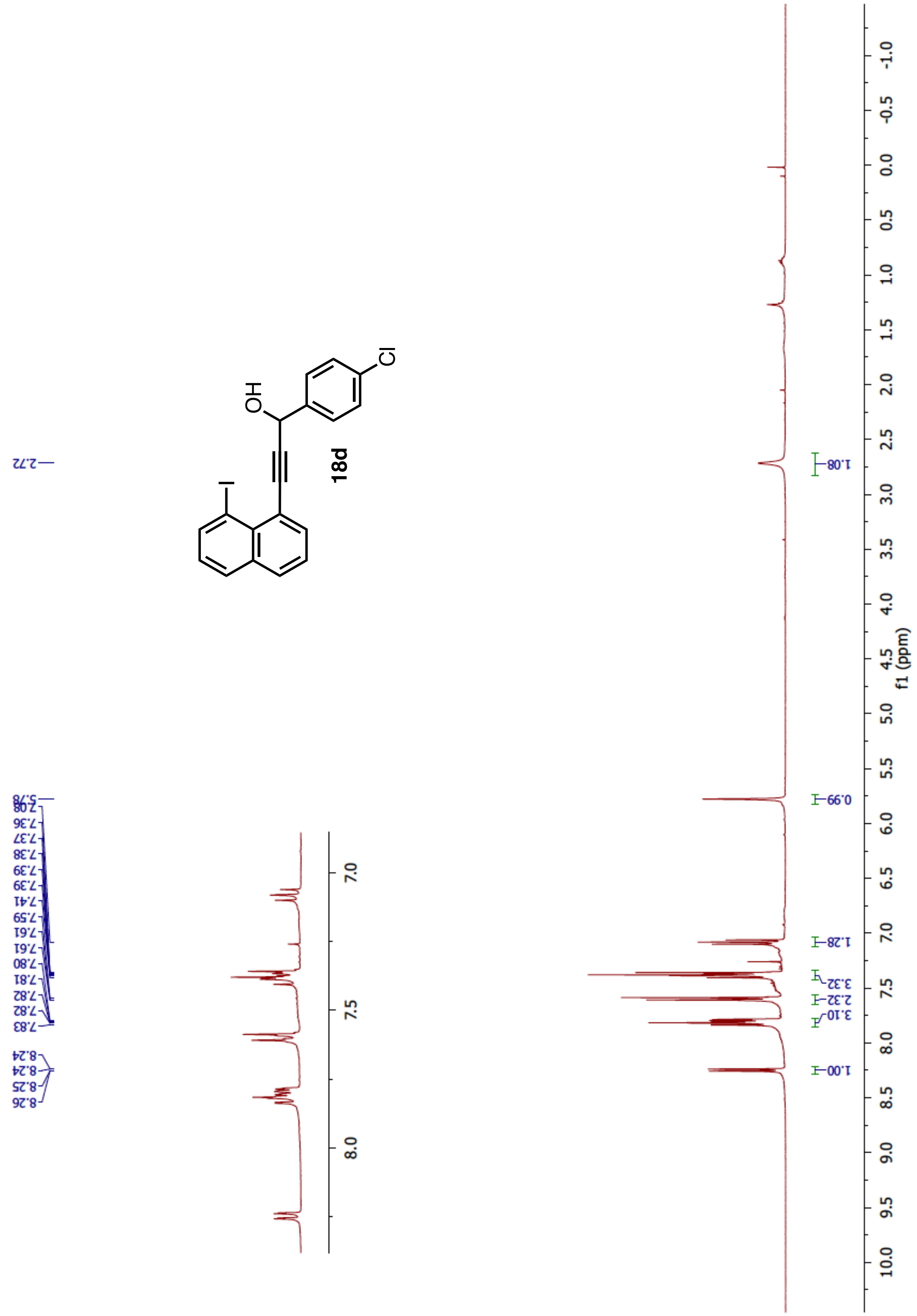

Figure S22. ^{1}H-NMR spectrum of **18d** in $CDCl_3$ (400 MHz).

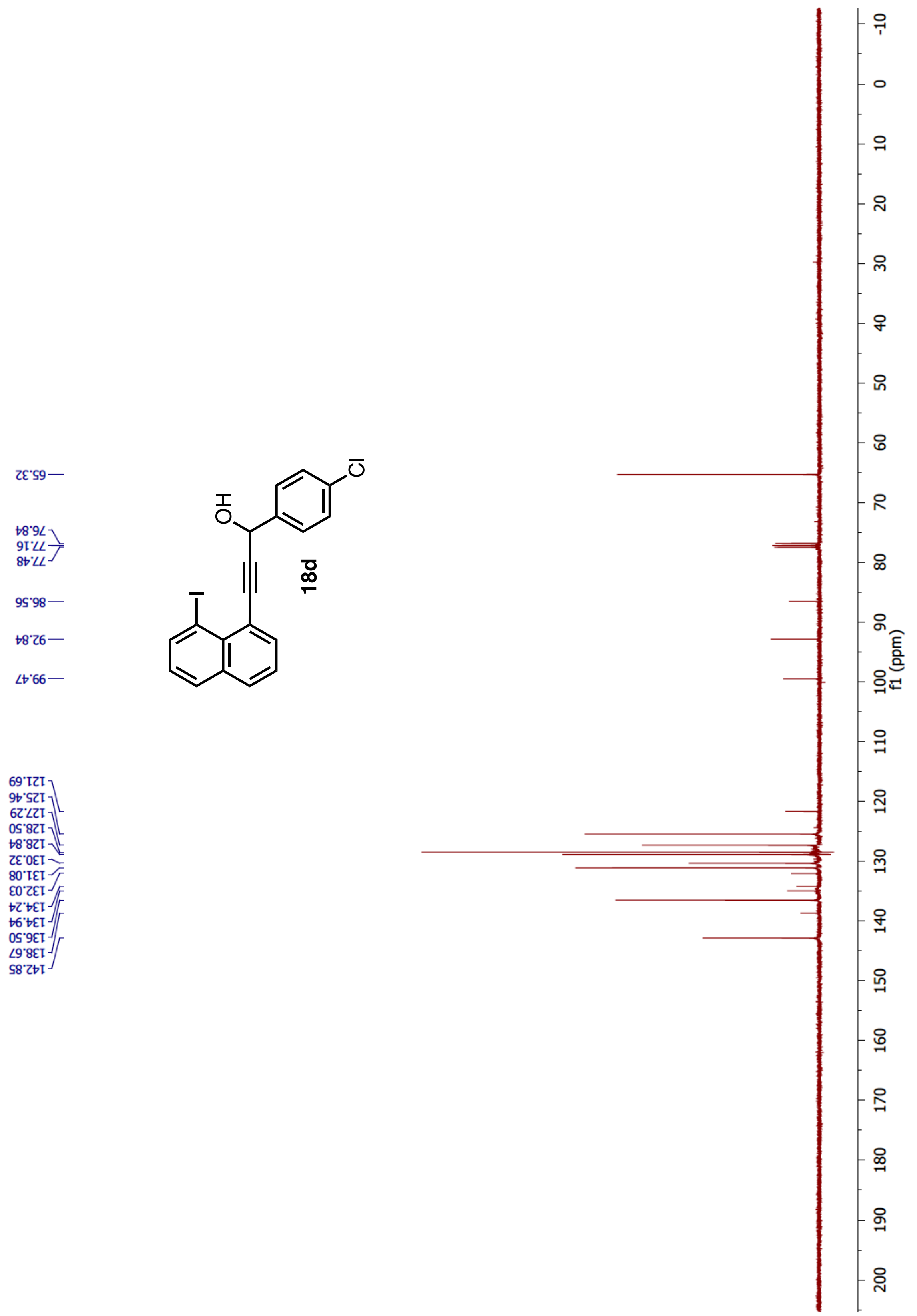

Figure S23. $^{13}C\{^{1}H\}$-NMR spectrum of **18d** in $CDCl_3$ (100 MHz).

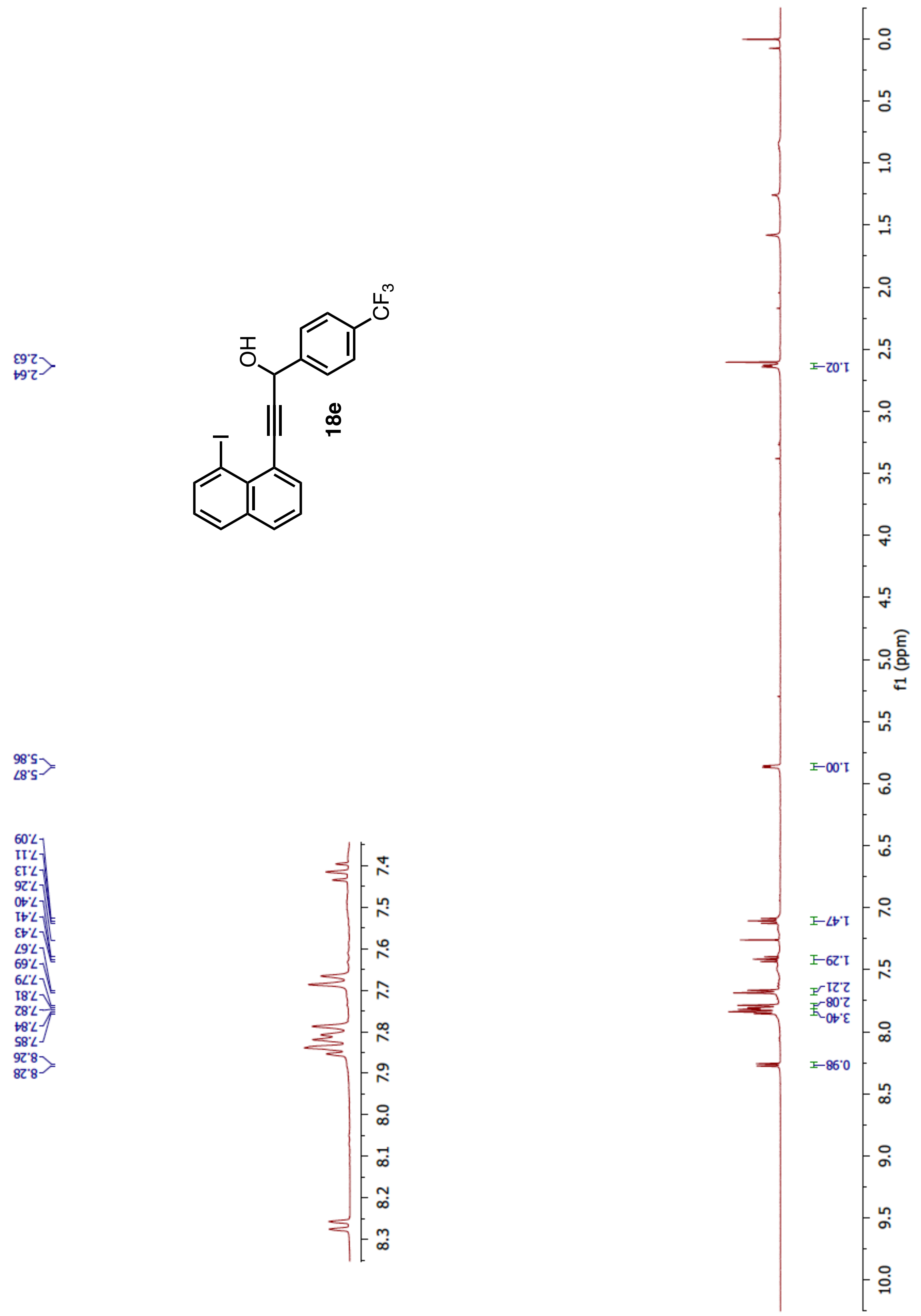

Figure S24. ^{1}H-NMR spectrum of **18e** in $CDCl_3$ (400 MHz).

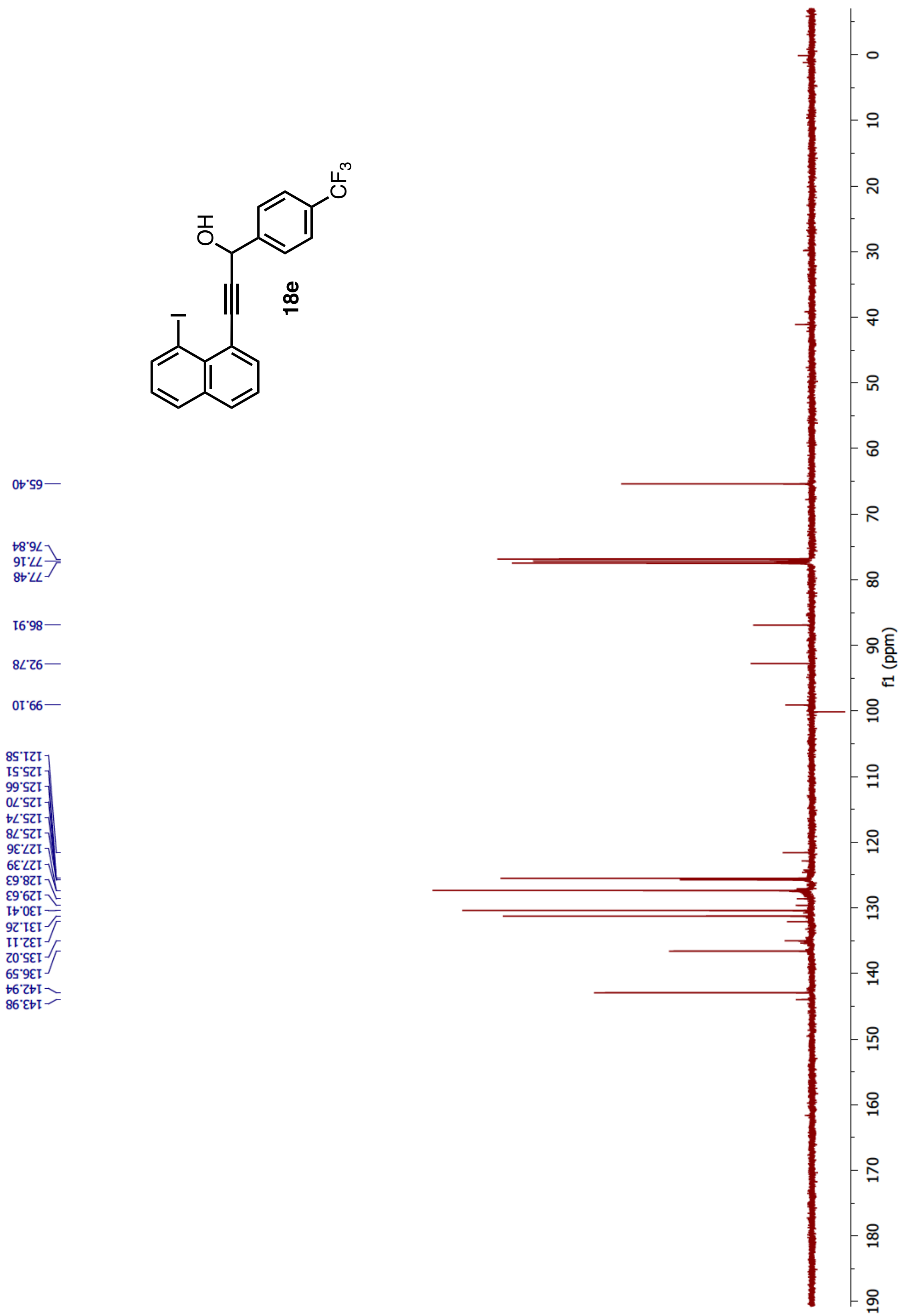

Figure S25. $^{13}C\{^{1}H\}$-NMR spectrum of **18e** in $CDCl_3$ (100 MHz).

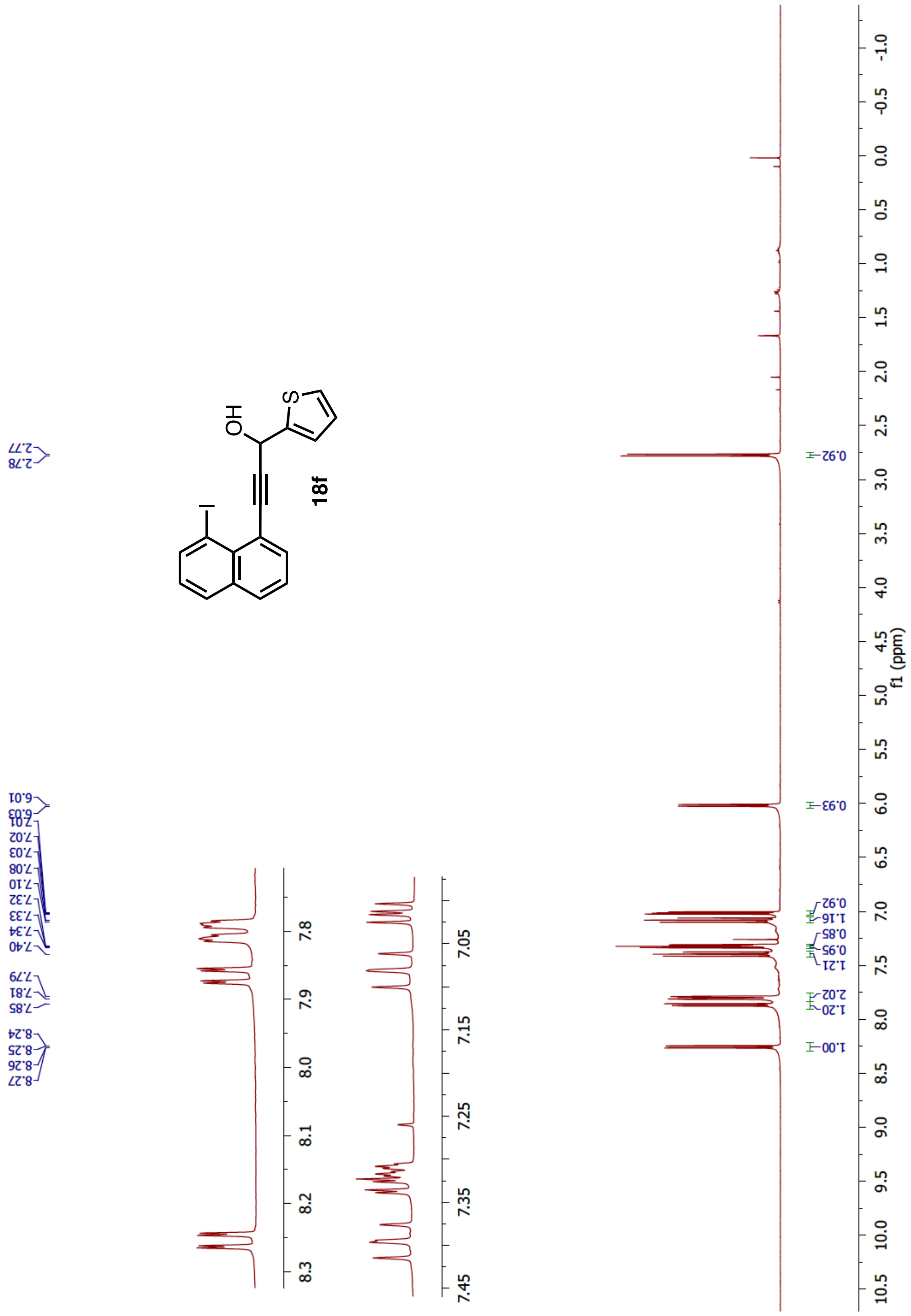

Figure S26. ^{1}H-NMR spectrum of **18f** in $CDCl_3$ (400 MHz).

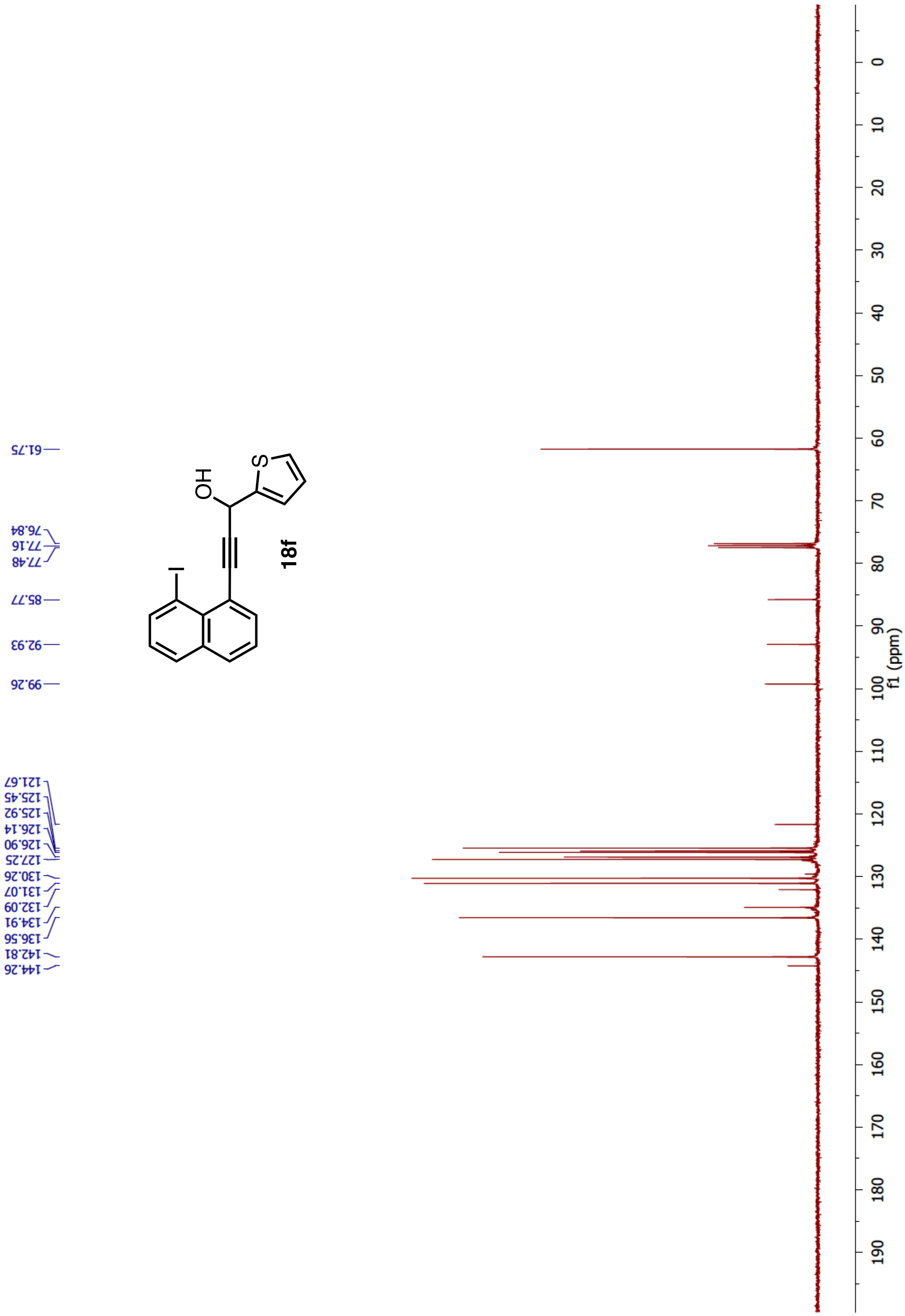

Figure S27. $^{13}C\{^{1}H\}$-NMR spectrum of **18f** in $CDCl_3$ (100 MHz).

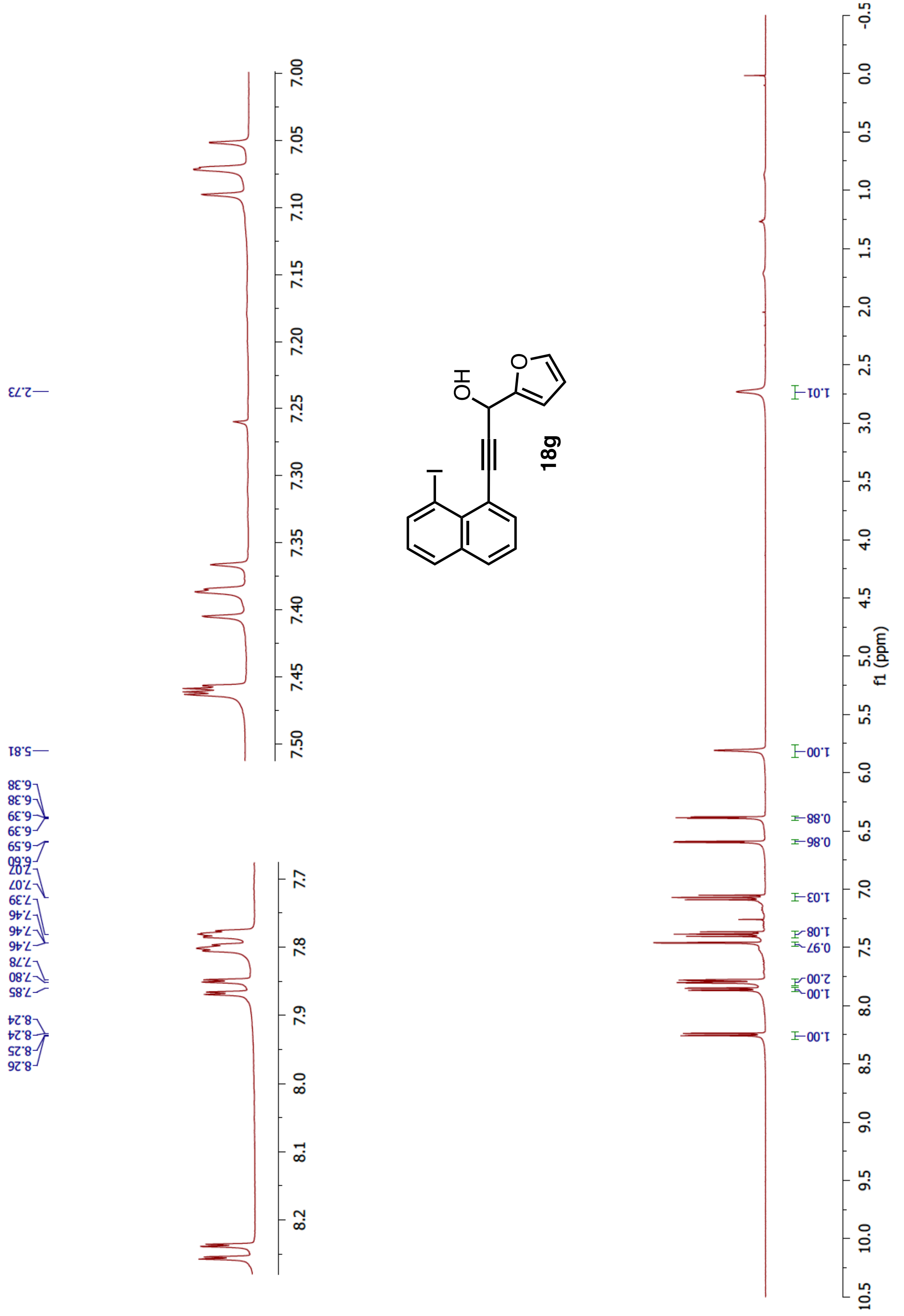

Figure S28. ^{1}H-NMR spectrum of **18g** in $CDCl_3$ (400 MHz).

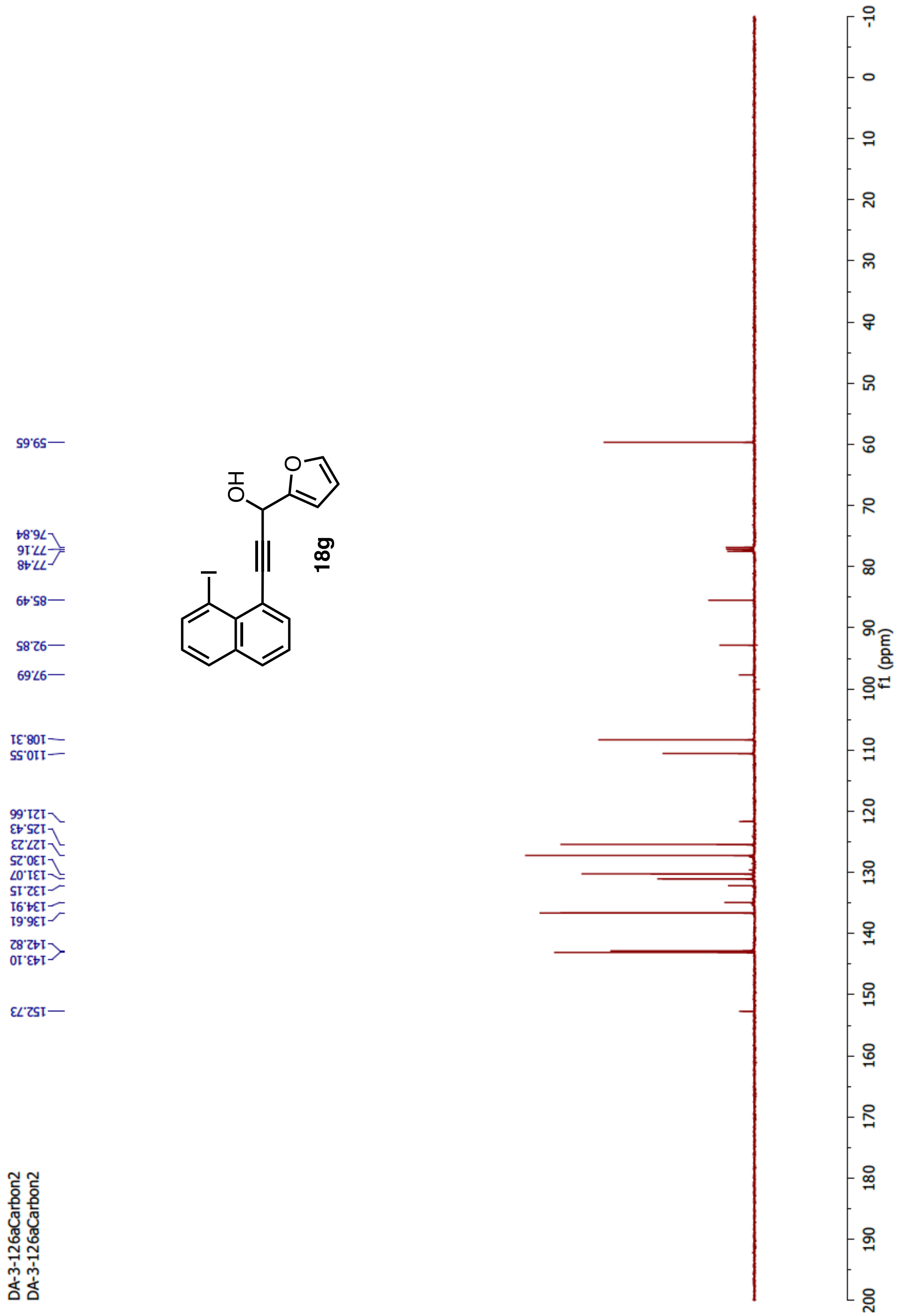

Figure S29. $^{13}C\{^{1}H\}$-NMR spectrum of **18g** in $CDCl_3$ (100 MHz).

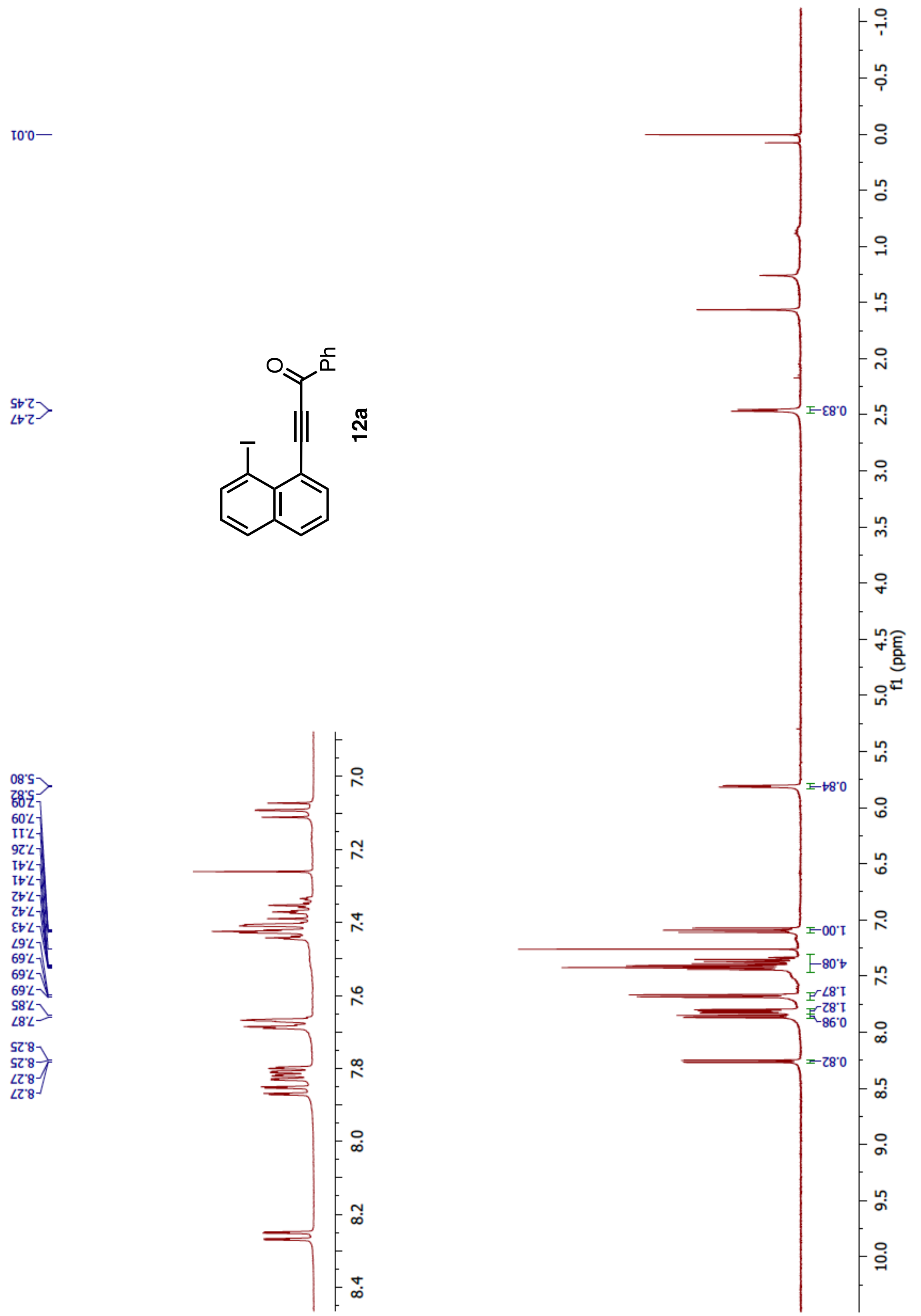

Figure S30. ^{1}H-NMR spectrum of **12a** in $CDCl_3$ (400 MHz).

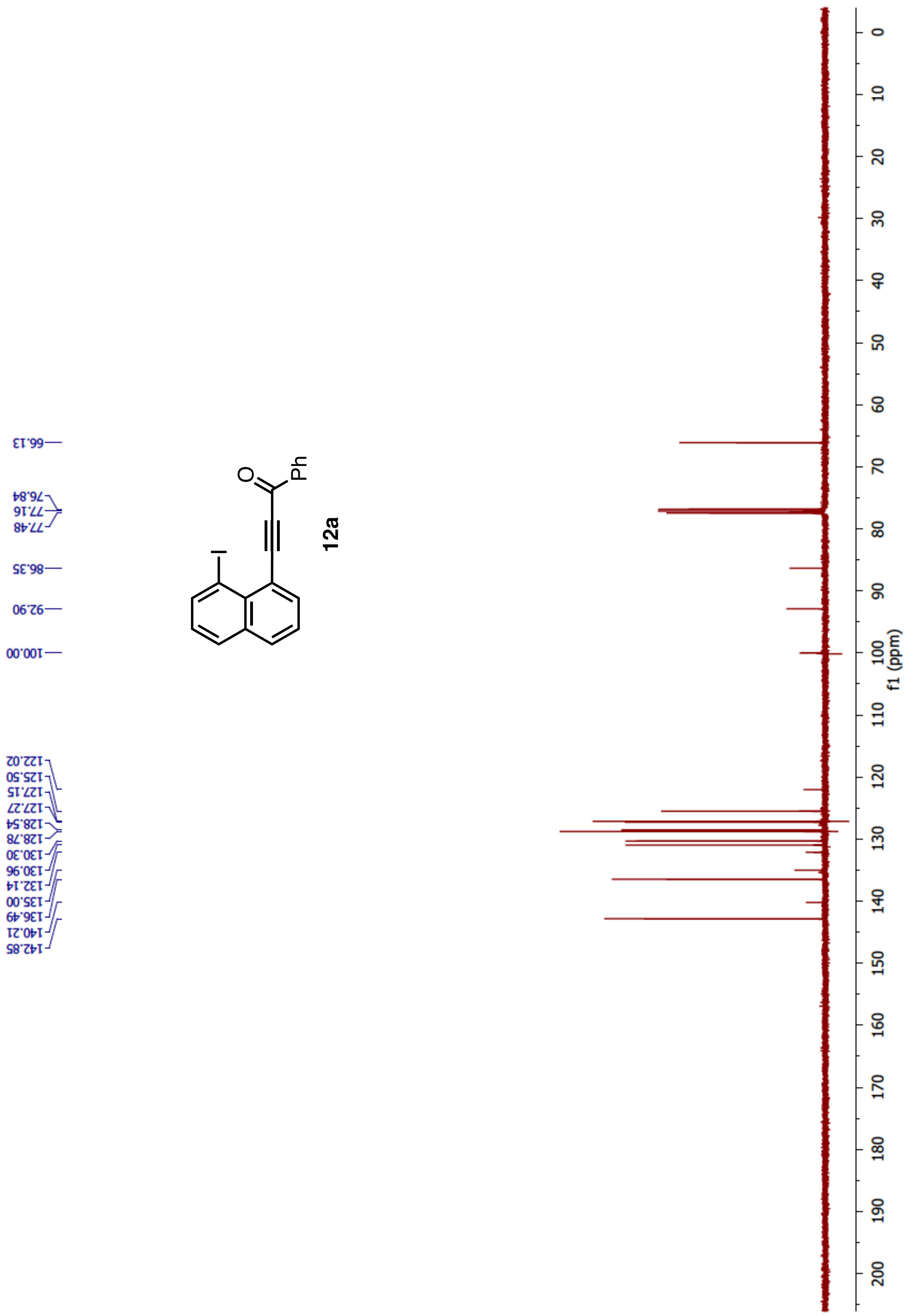

Figure S31. $^{13}C\{^1H\}$-NMR spectrum of **12a** in $CDCl_3$ (100 MHz).

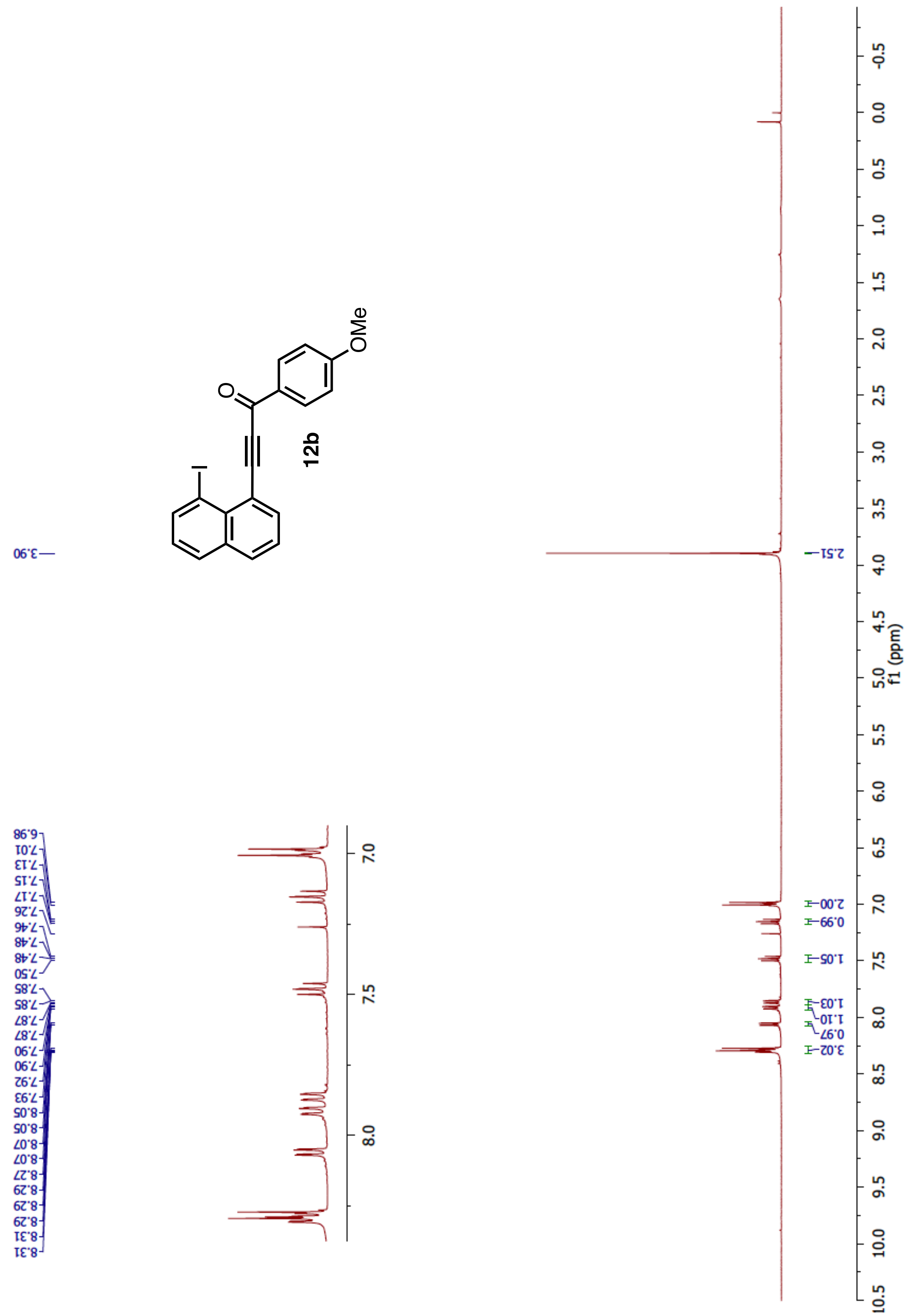

Figure S32. ^{1}H-NMR spectrum of **12b** in $CDCl_3$ (400 MHz).

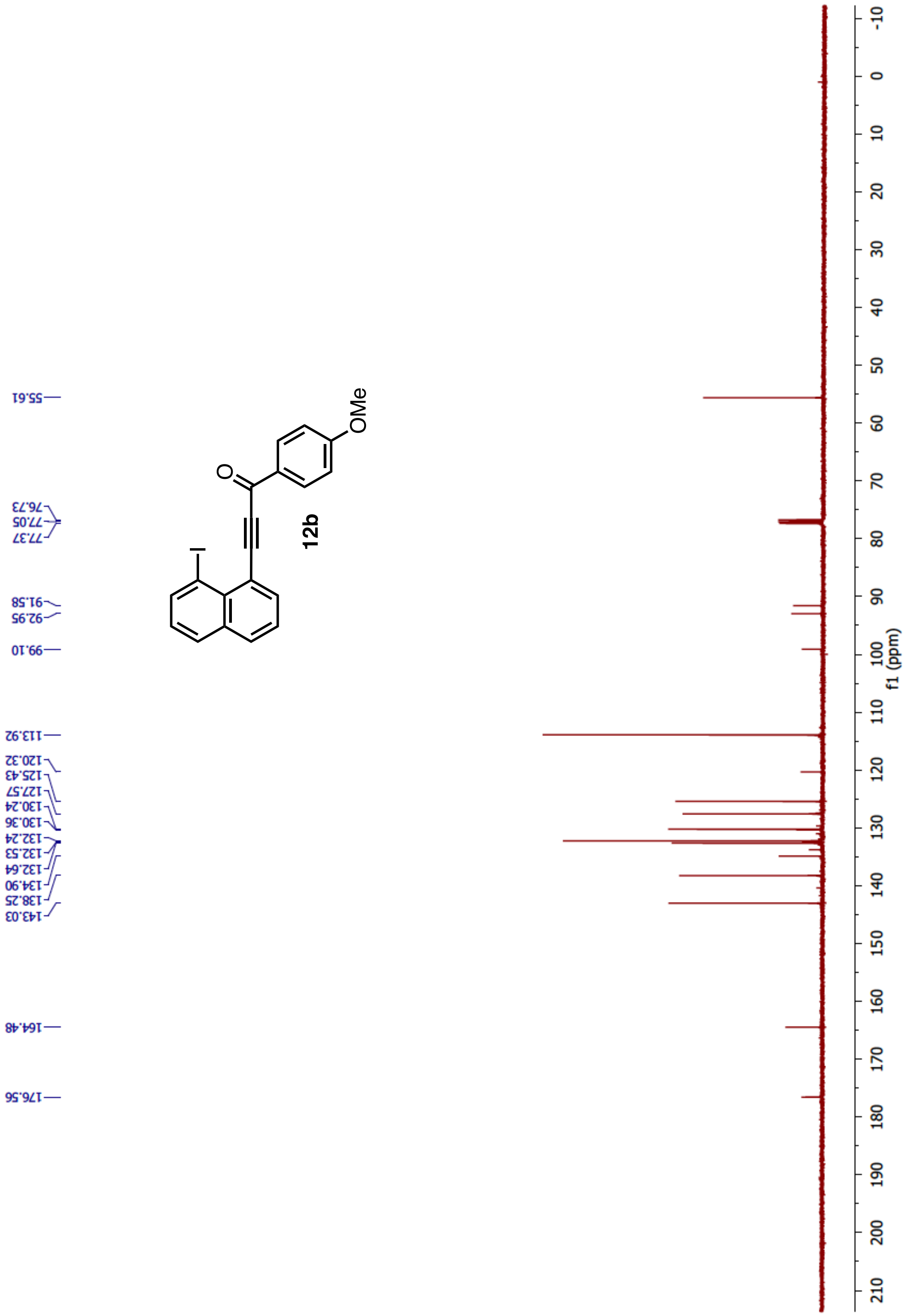

Figure S33. $^{13}C\{^1H\}$-NMR spectrum of **12b** in $CDCl_3$ (100 MHz).

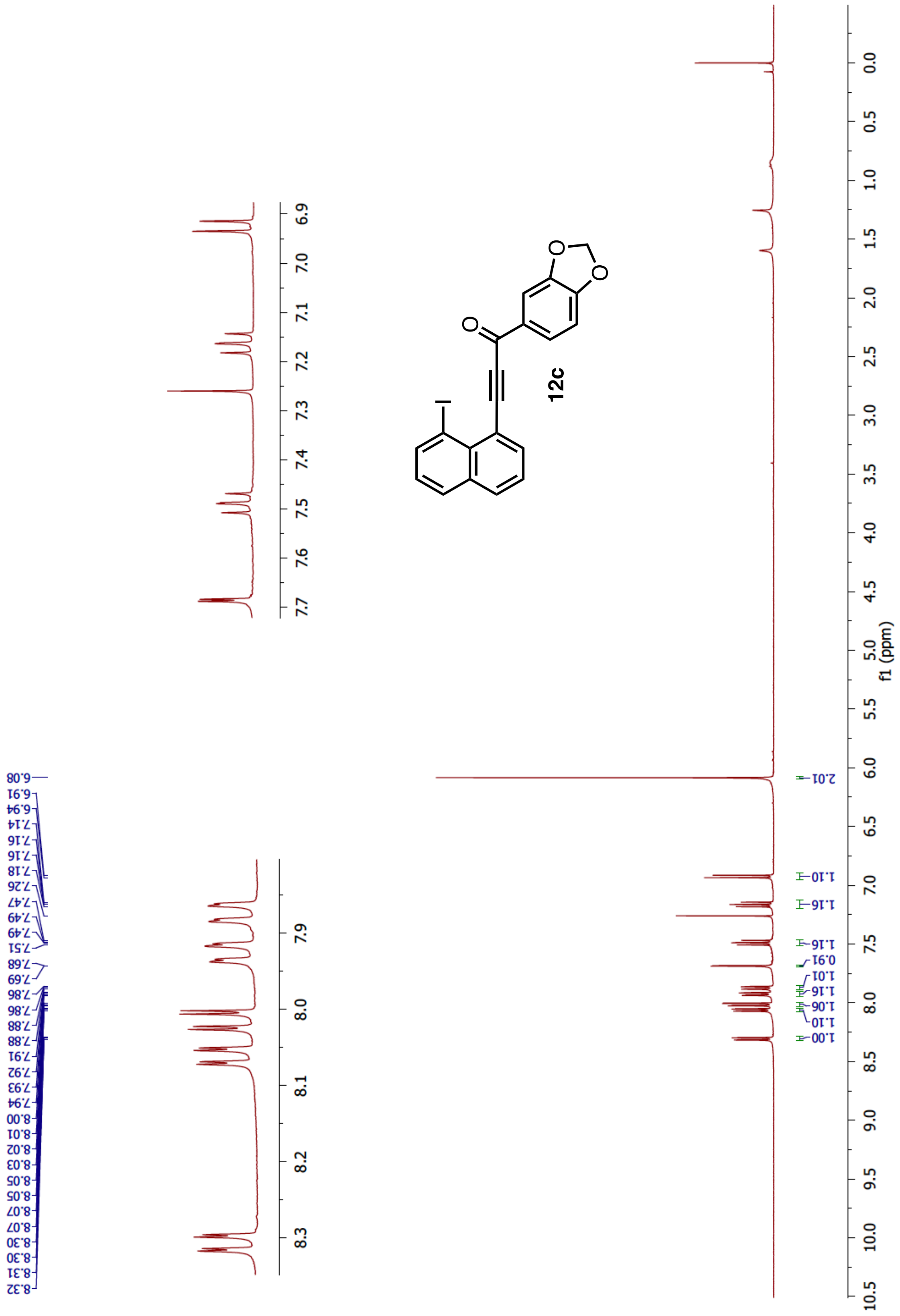

Figure S34. ^{1}H-NMR spectrum of **12c** in $CDCl_3$ (400 MHz).

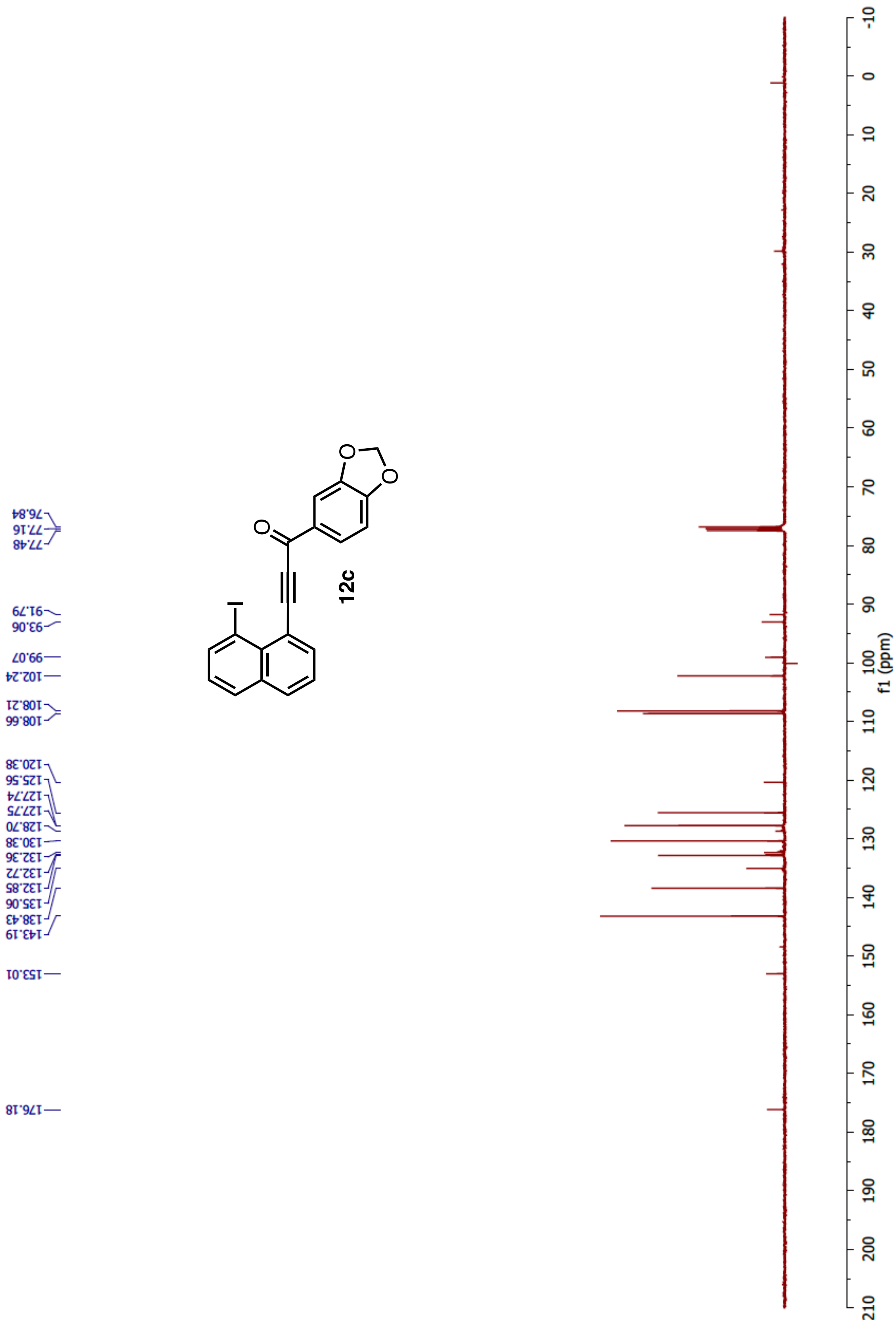

Figure S35. $^{13}C\{^{1}H\}$-NMR spectrum of **12c** in $CDCl_3$ (100 MHz).

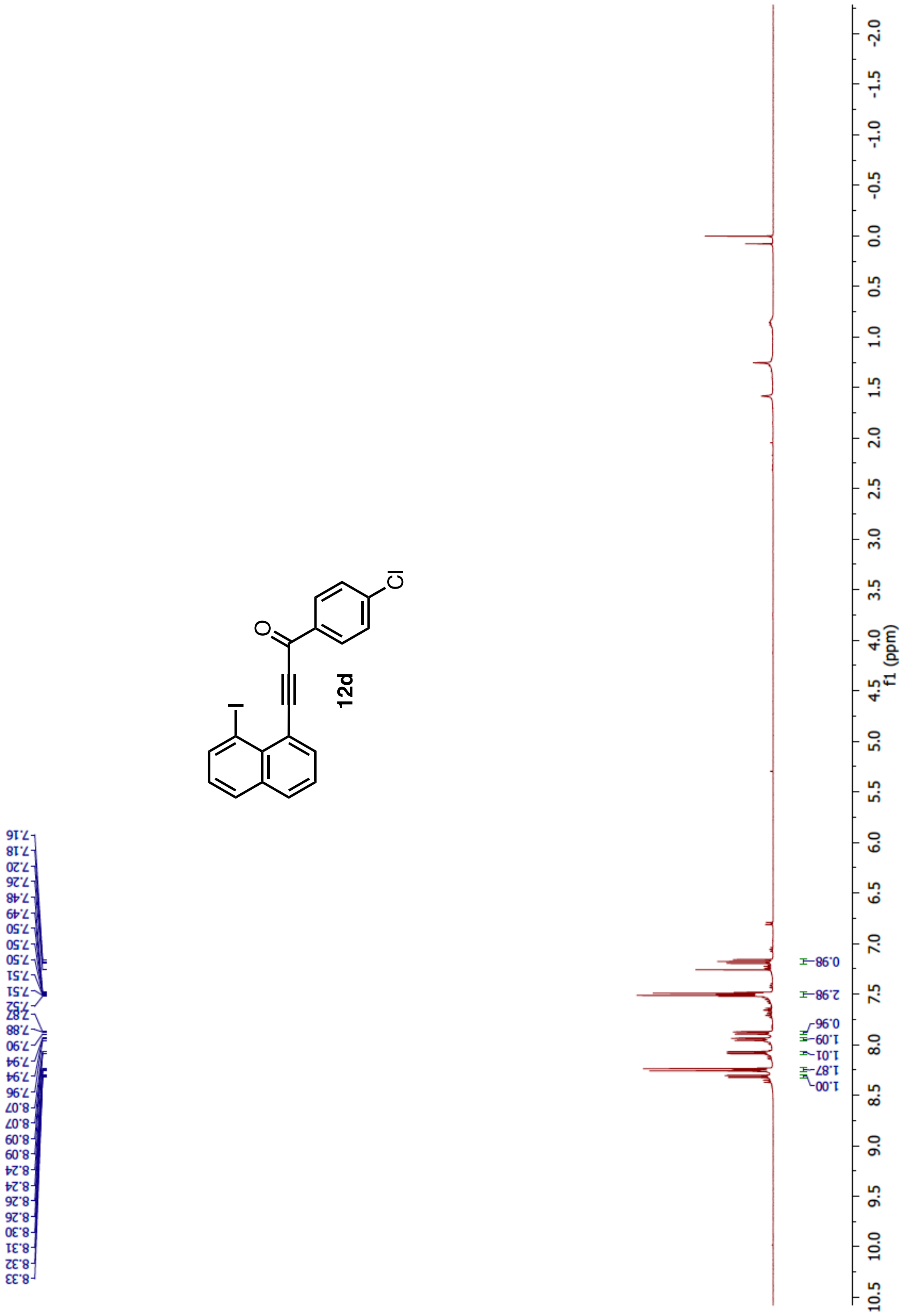

Figure S36. ^{1}H-NMR spectrum of **12d** in $CDCl_3$ (400 MHz).

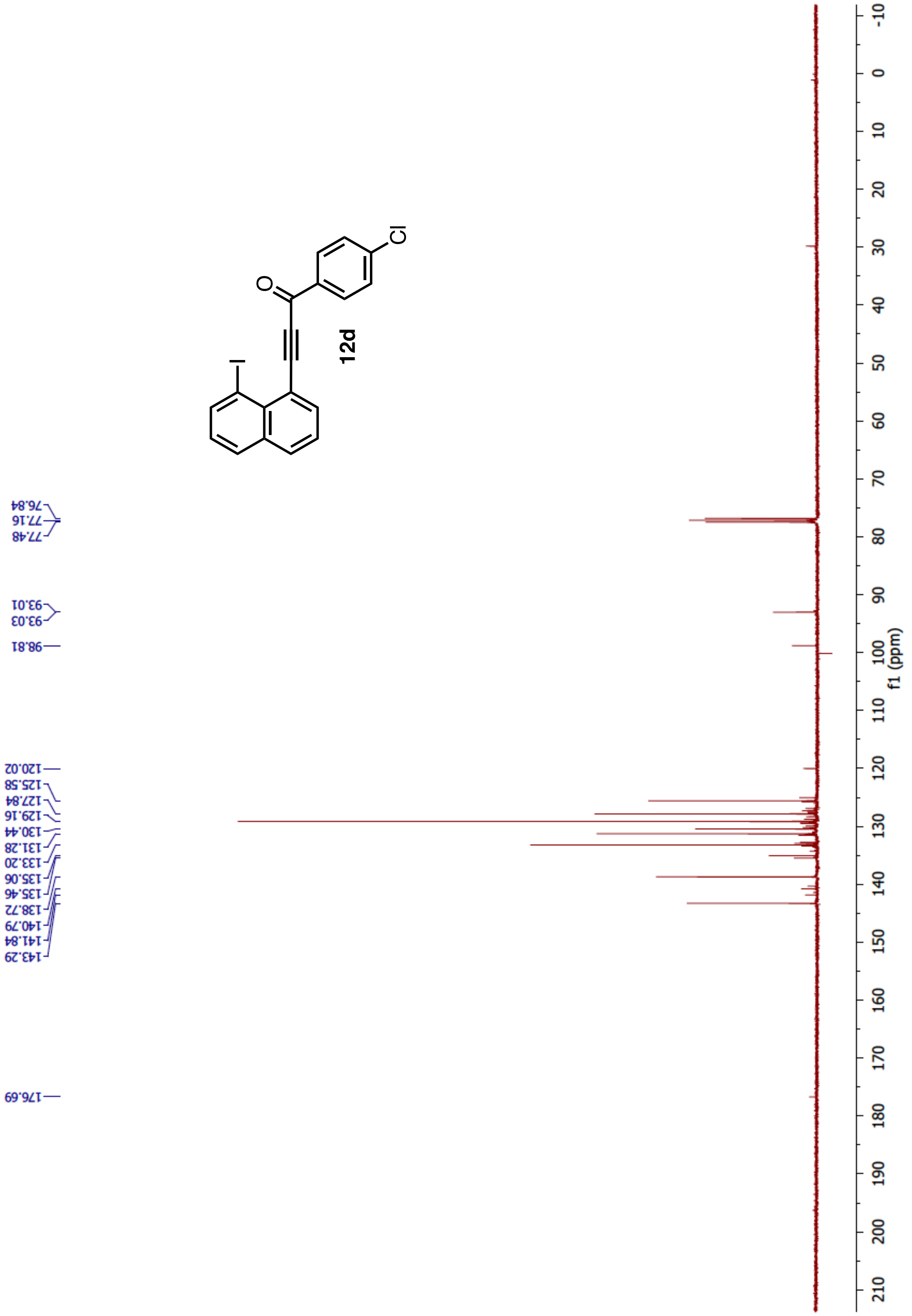

Figure S37. $^{13}C\{^{1}H\}$-NMR spectrum of **12d** in $CDCl_3$ (100 MHz).

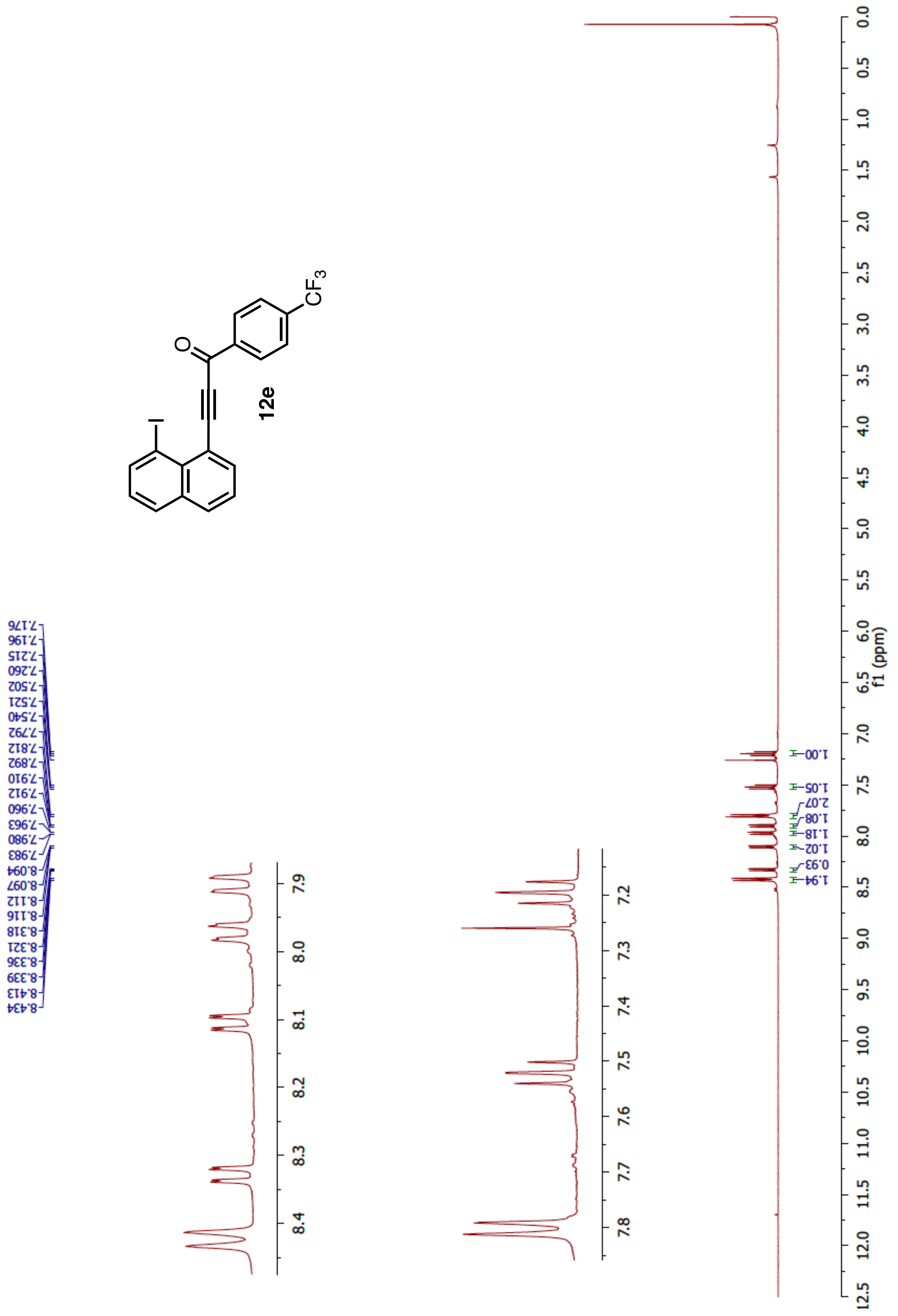

Figure S38. ^{1}H-NMR spectrum of **12e** in $CDCl_3$ (400 MHz).

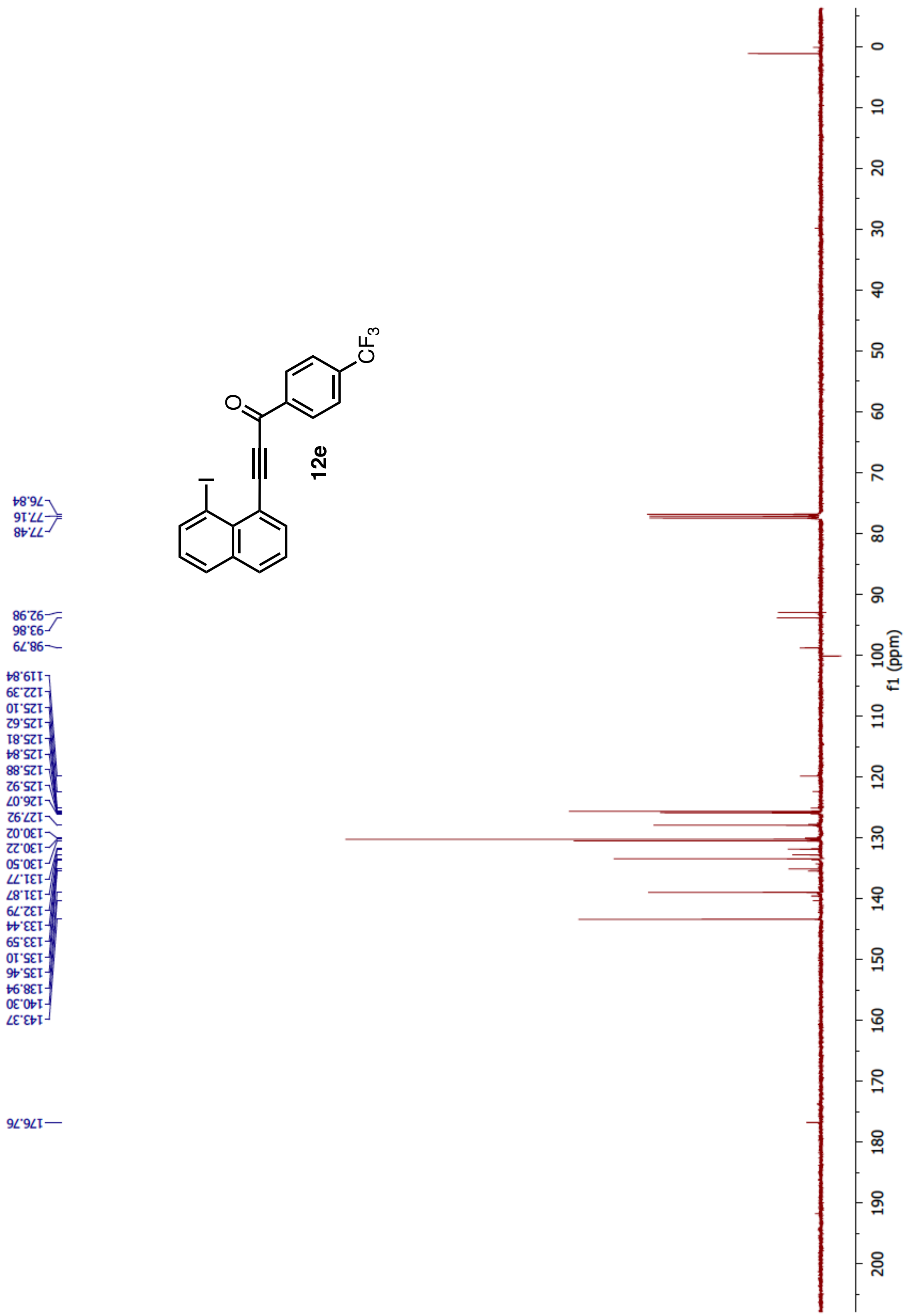

Figure S39. $^{13}C\{^{1}H\}$-NMR spectrum of **12e** in $CDCl_3$ (100 MHz).

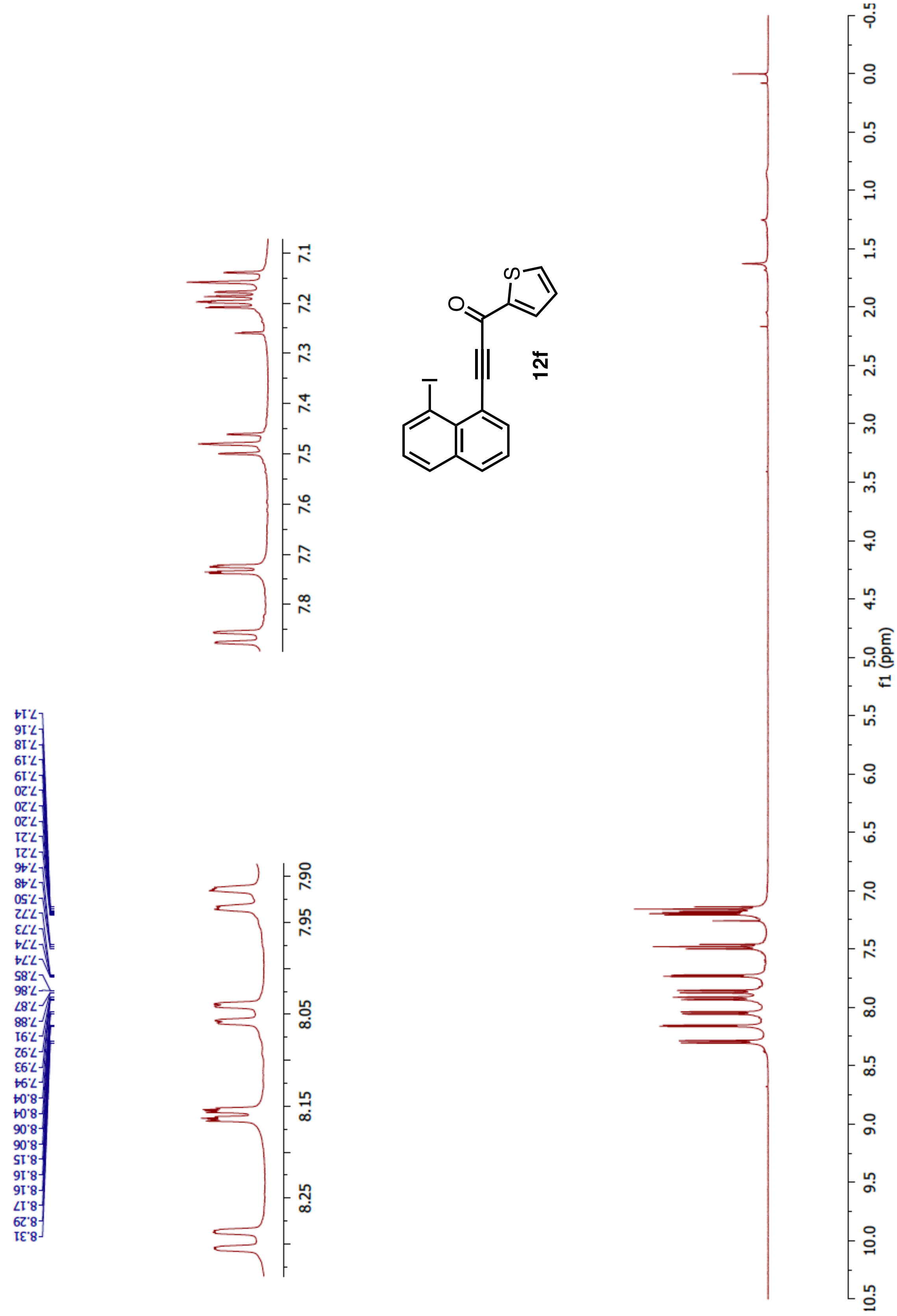

Figure S40. ^{1}H-NMR spectrum of **12f** in $CDCl_3$ (400 MHz).

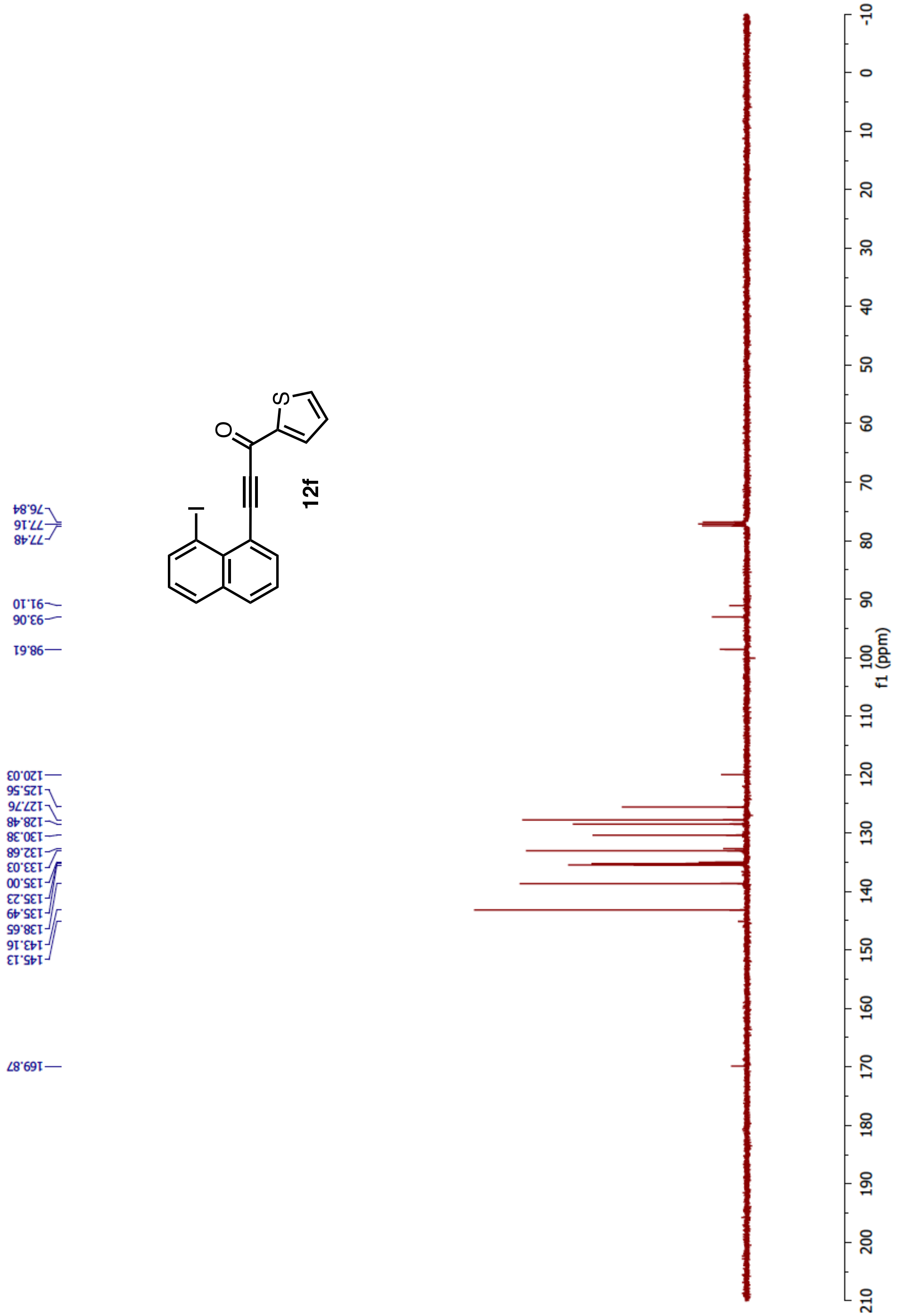

Figure S41. $^{13}C\{^{1}H\}$-NMR spectrum of **12f** in $CDCl_3$ (100 MHz).

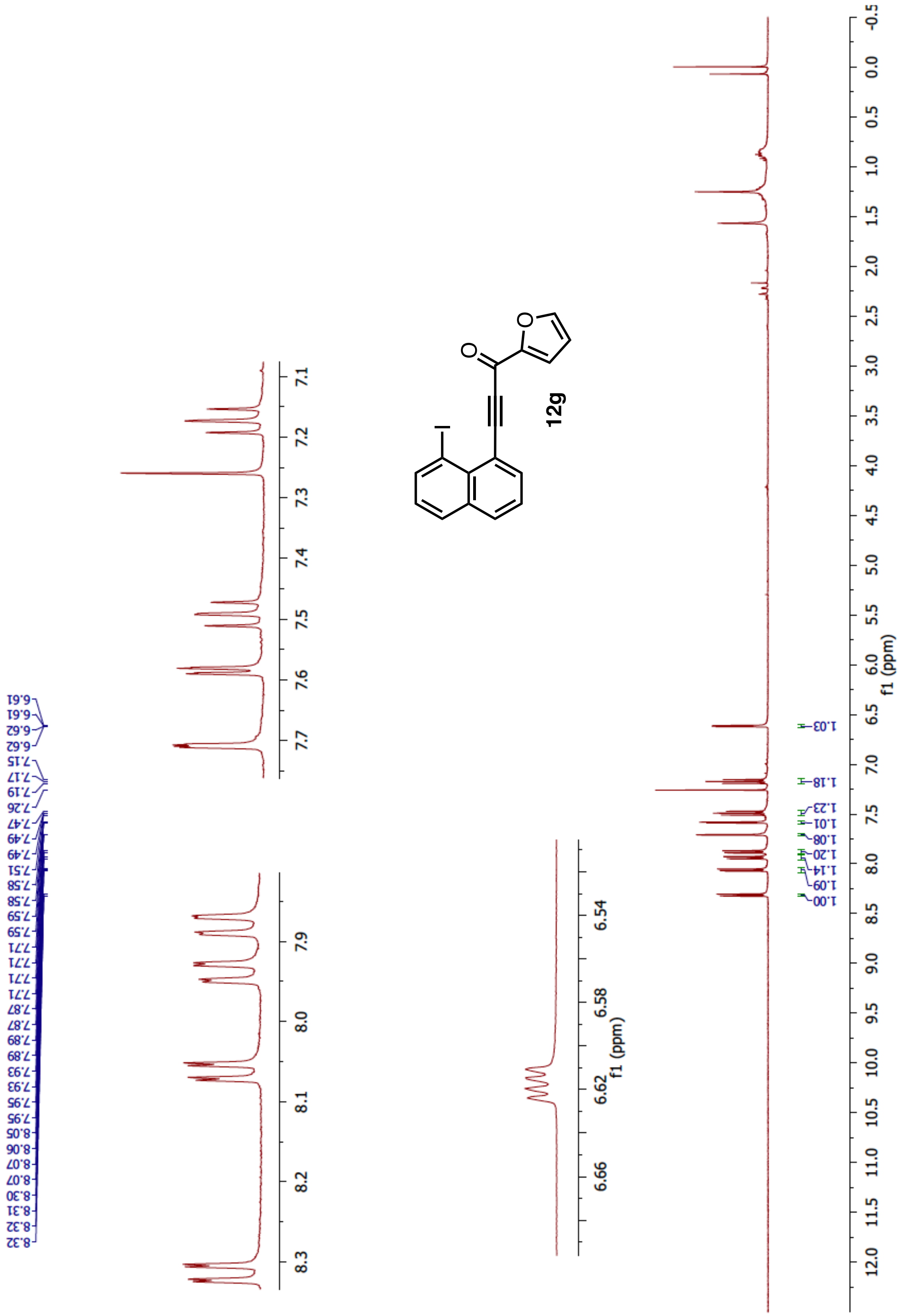

Figure S42. ^{1}H-NMR spectrum of **12g** in $CDCl_3$ (400 MHz).

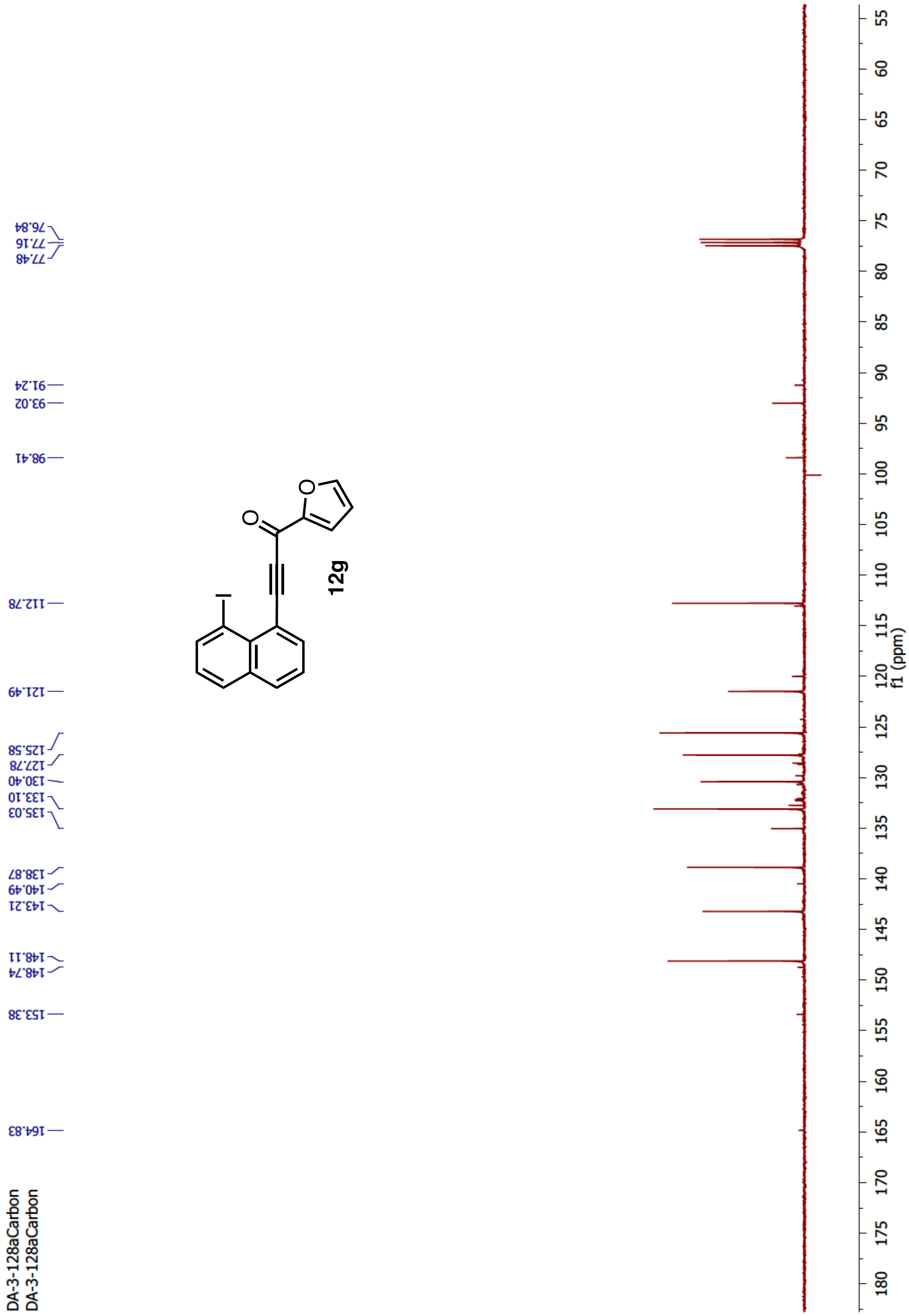

Figure S43. $^{13}C\{^{1}H\}$-NMR spectrum of **12g** in $CDCl_3$ (100 MHz).

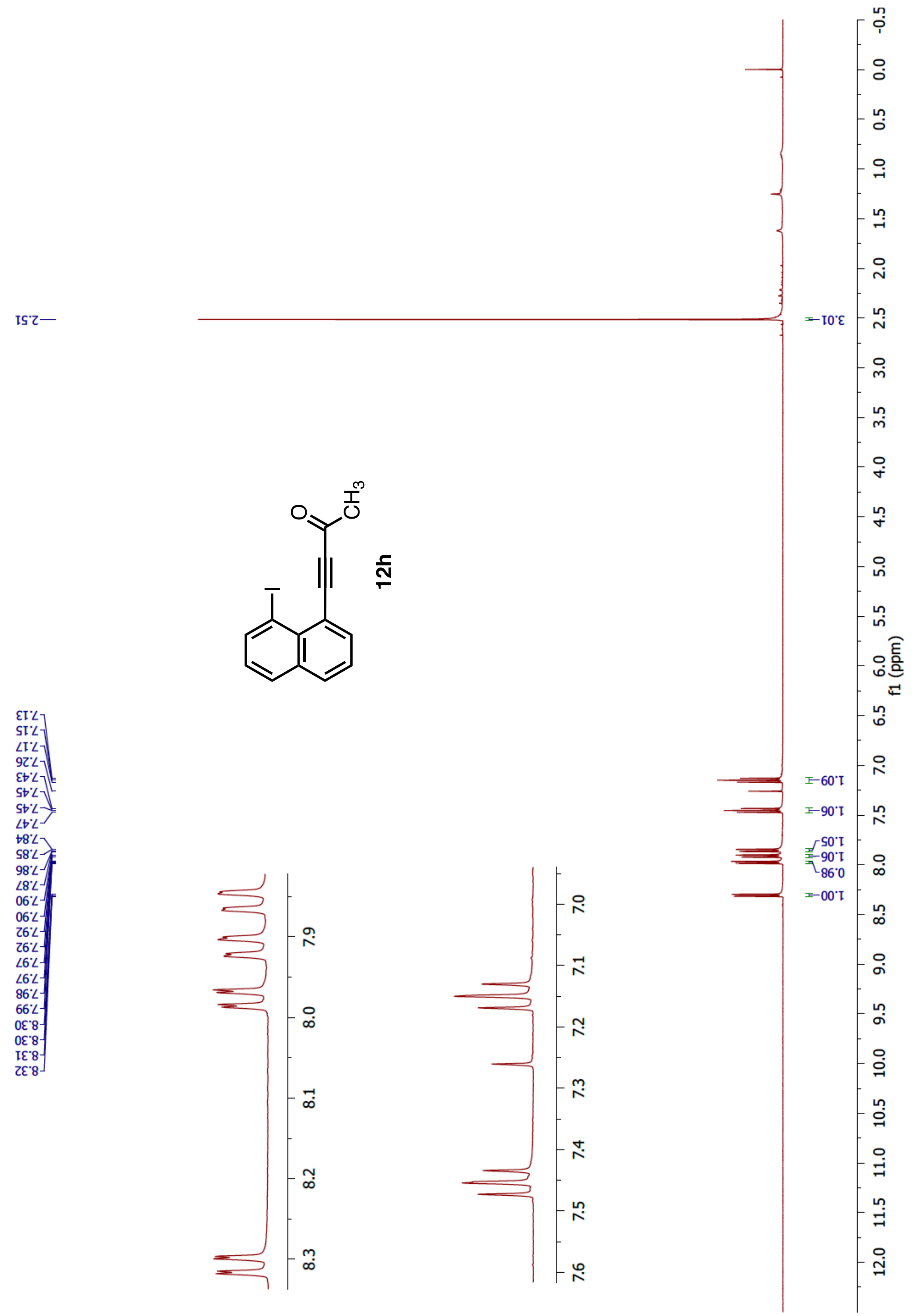

Figure S44. ^{1}H-NMR spectrum of **12h** in $CDCl_3$ (400 MHz).

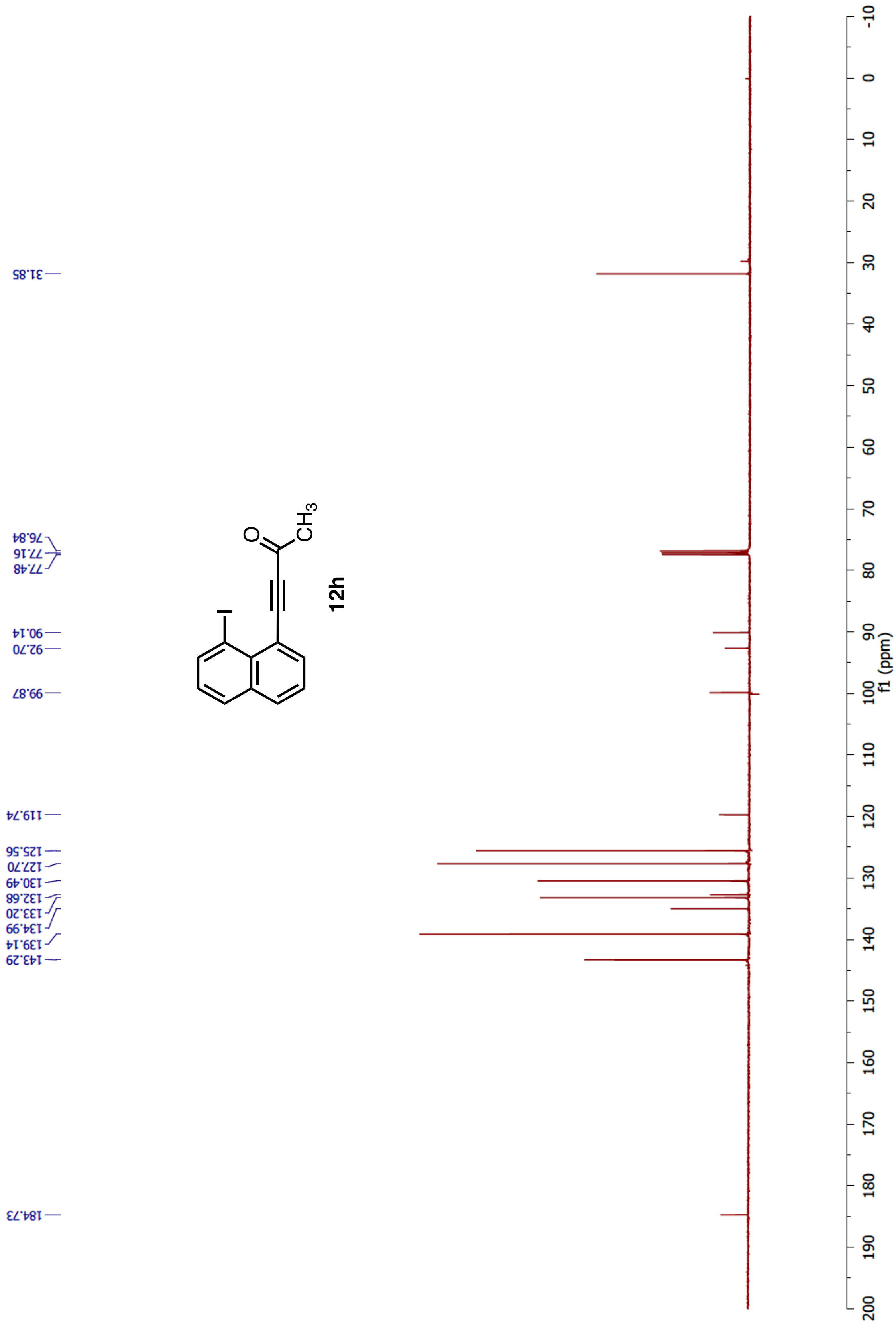

Figure S45. $^{13}C\{^{1}H\}$-NMR spectrum of **12h** in $CDCl_3$ (100 MHz).

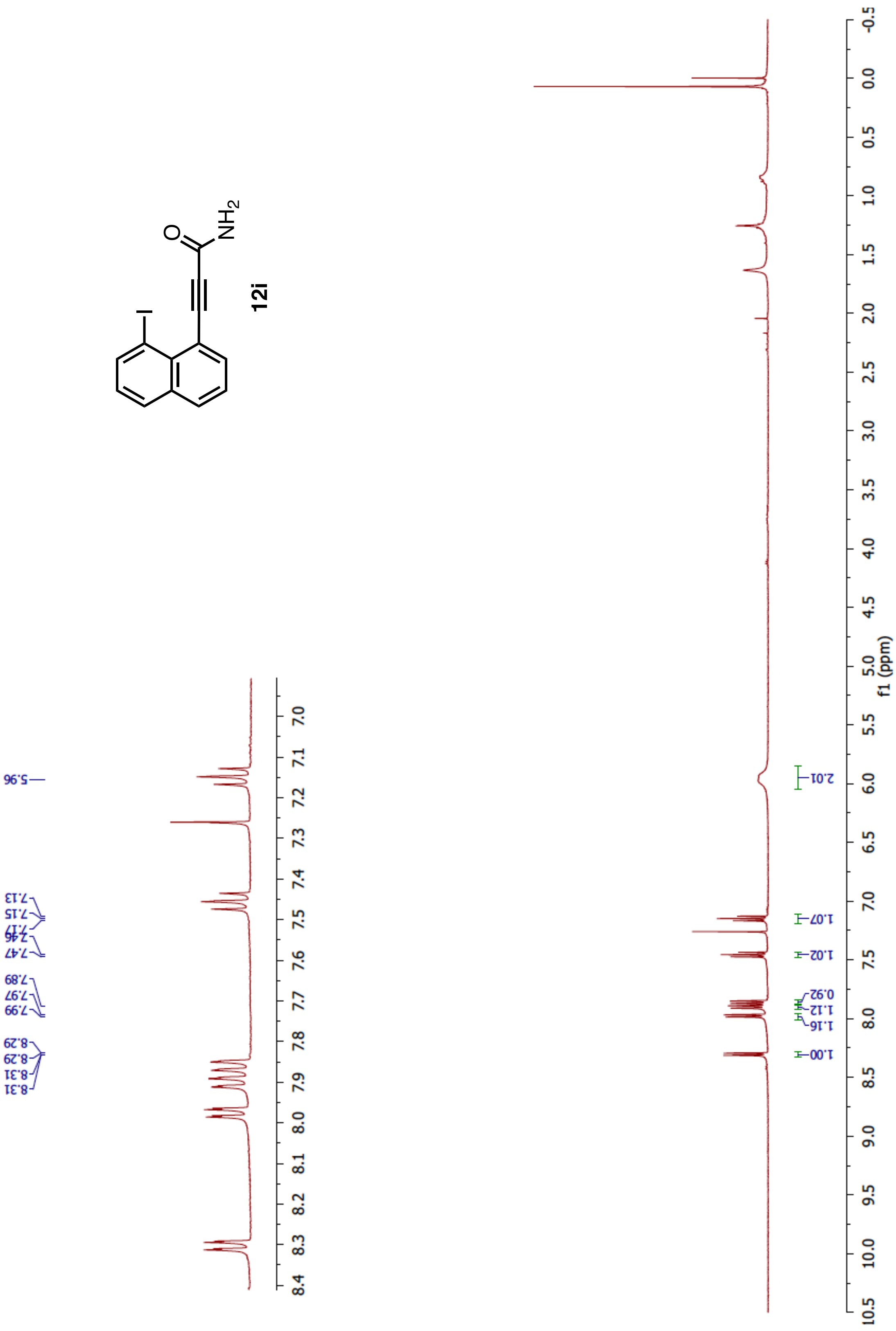

Figure S46. ^{1}H-NMR spectrum of **12i** in $CDCl_3$ (400 MHz).

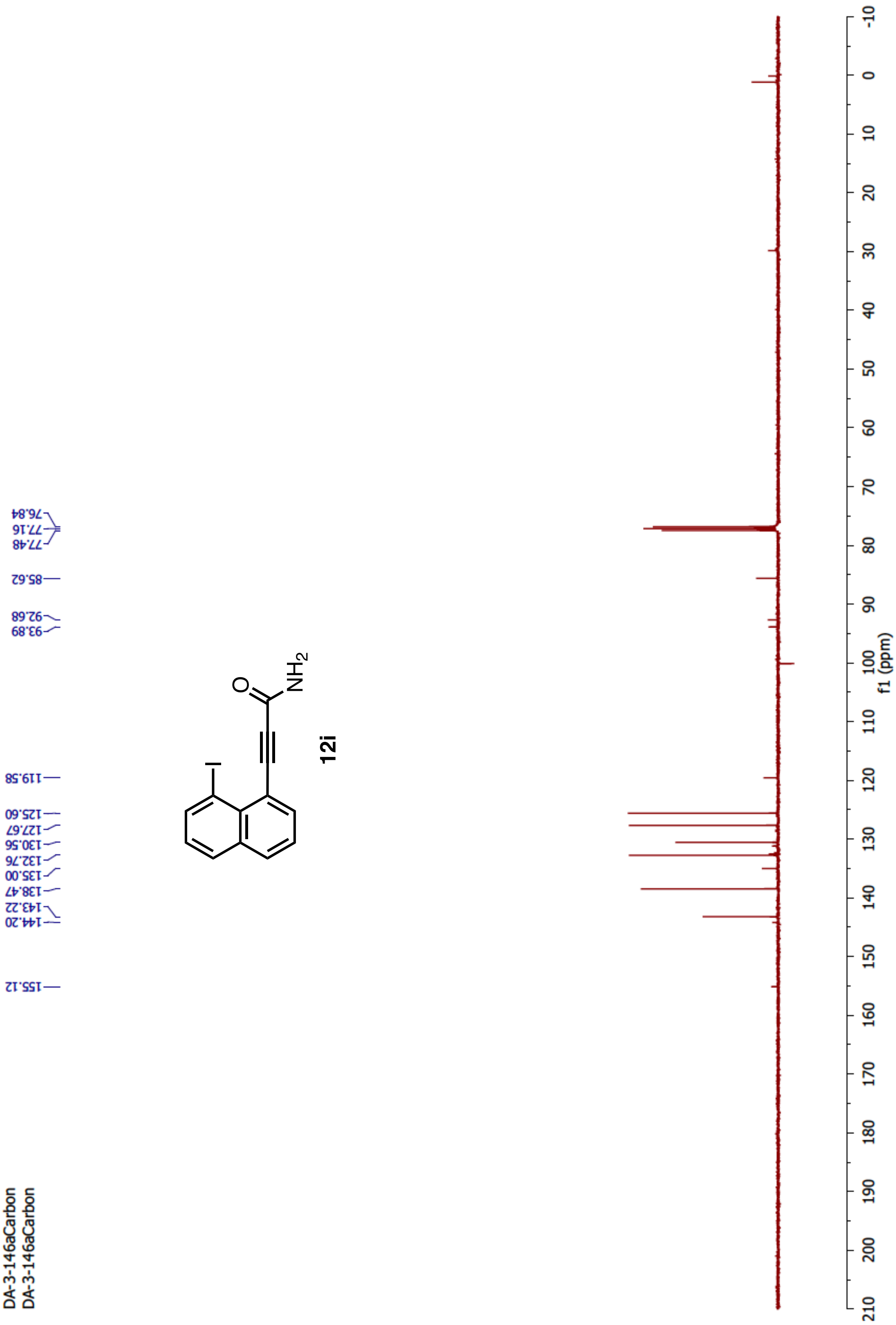

Figure S47. $^{13}C\{^{1}H\}$-NMR spectrum of **12i** in $CDCl_3$ (100 MHz).

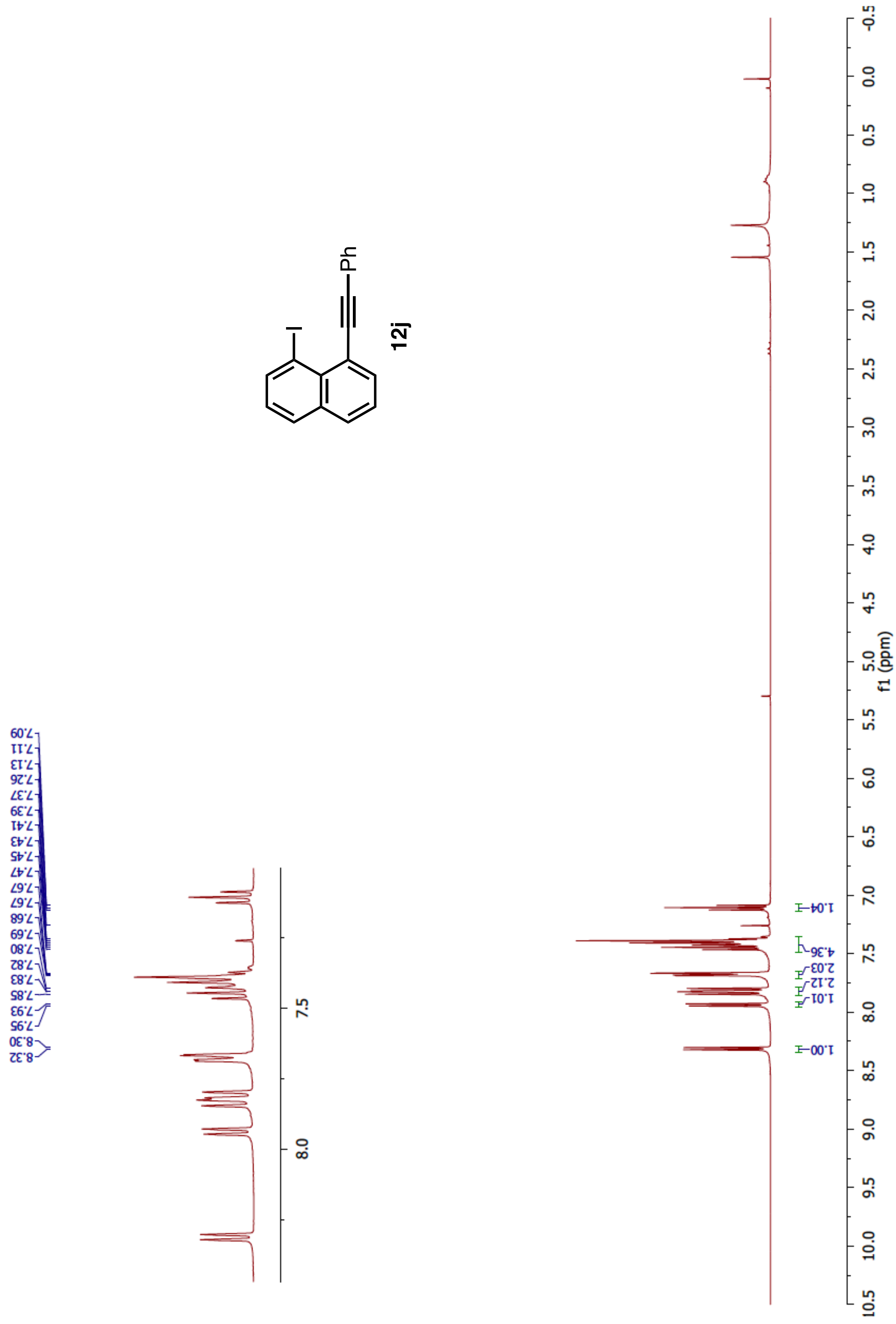

Figure S48. ^{1}H-NMR spectrum of **12j** in $CDCl_3$ (400 MHz).

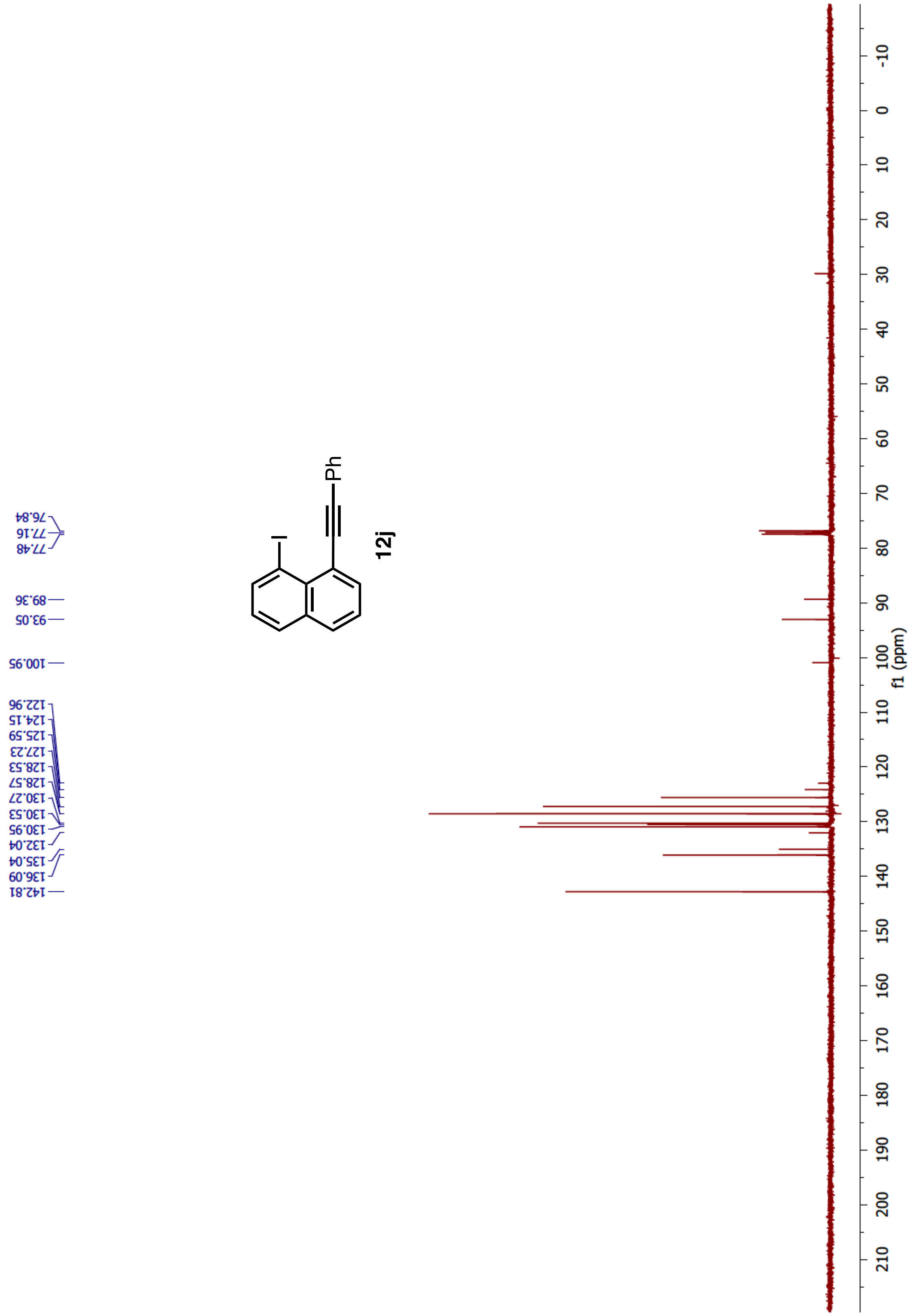

Figure S49. $^{13}C\{^1H\}$-NMR spectrum of **12j** in $CDCl_3$ (100 MHz).

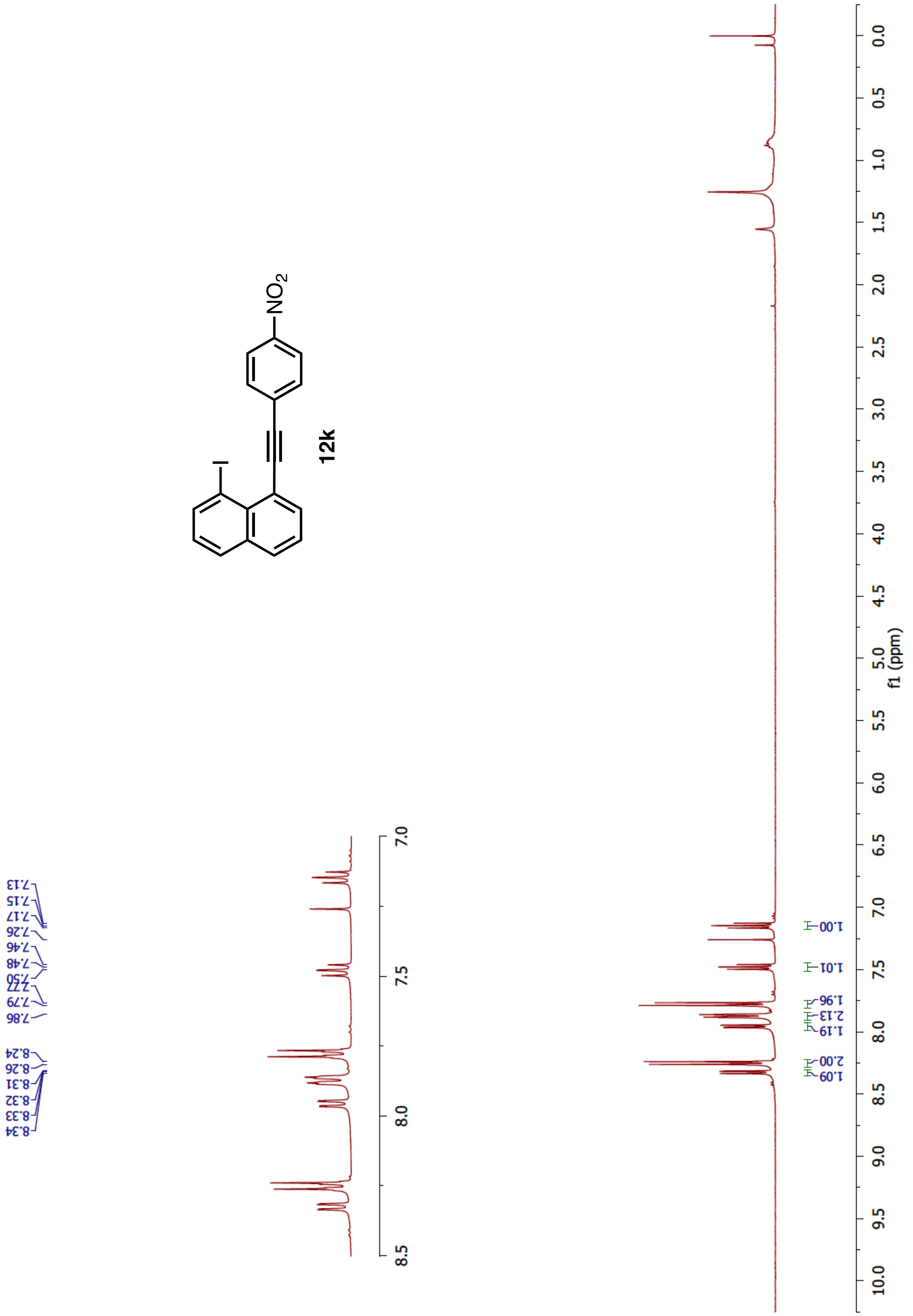

Figure S50. ^{1}H-NMR spectrum of **12k** in $CDCl_3$ (400 MHz).

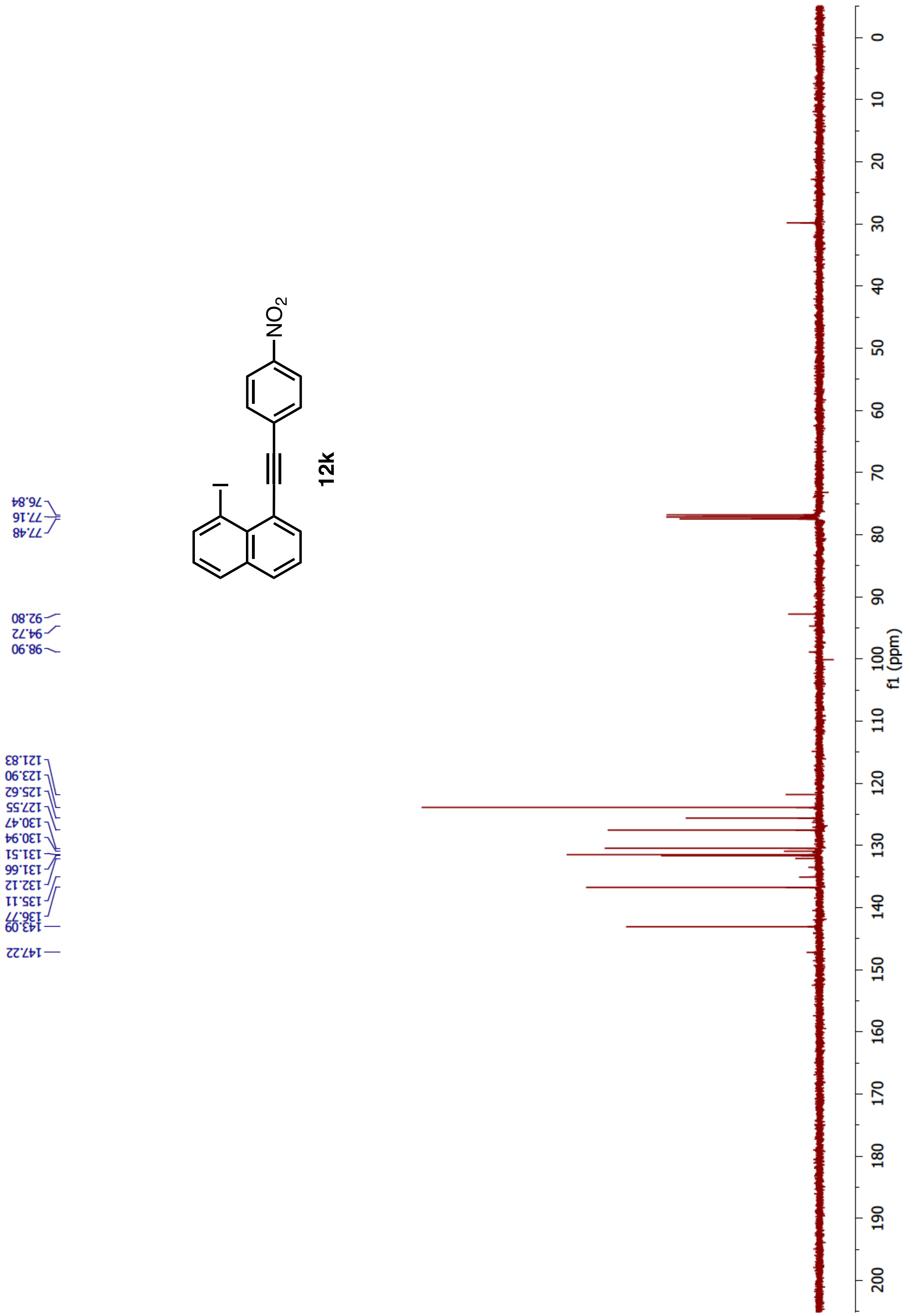

Figure S51. $^{13}C\{^1H\}$-NMR spectrum of **12k** in $CDCl_3$ (100 MHz).

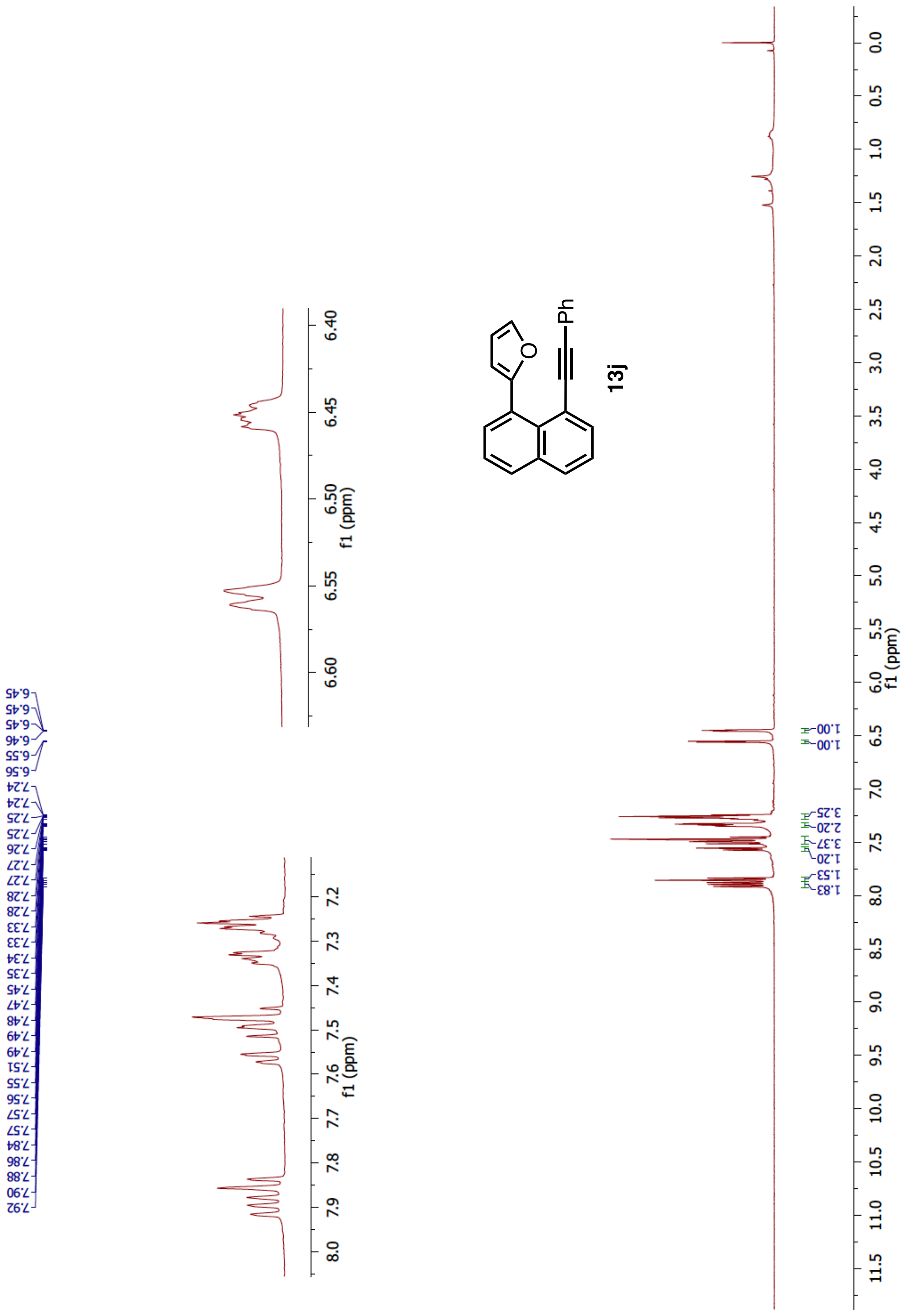

Figure S52. ^{1}H-NMR spectrum of **13j** in $CDCl_3$ (400 MHz).

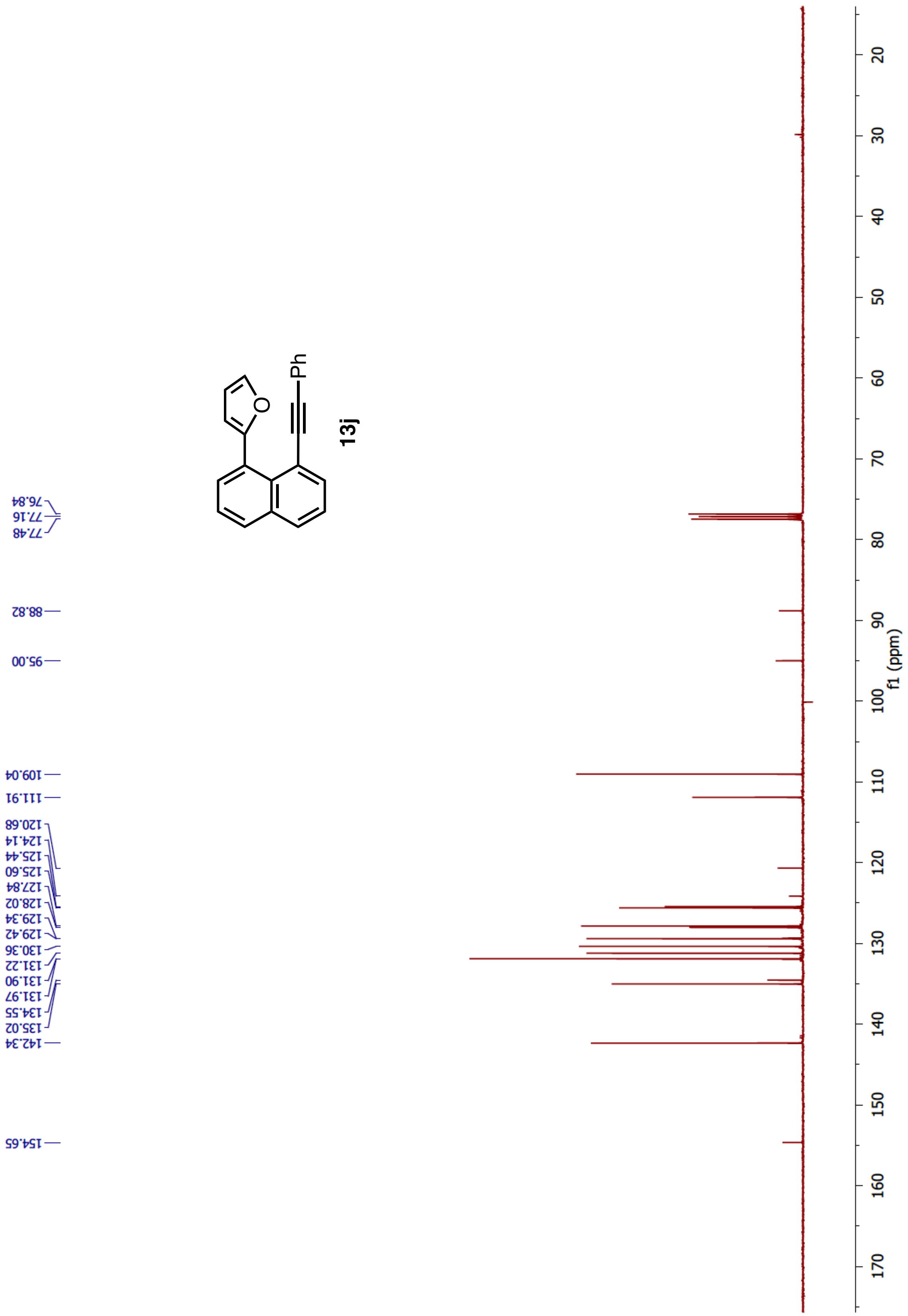

Figure S53. $^{13}C\{^{1}H\}$-NMR spectrum of **13j** in $CDCl_3$ (100 MHz).

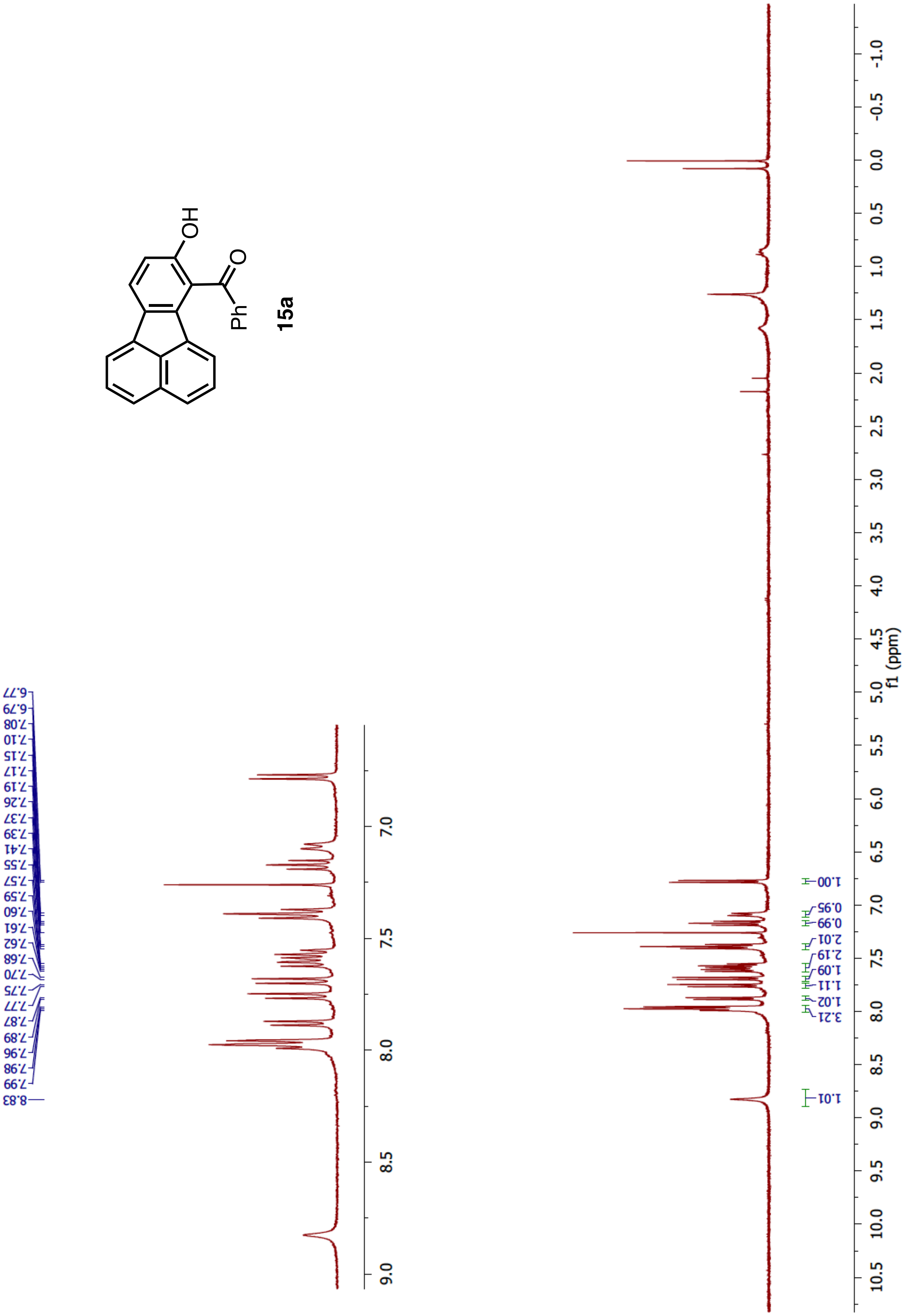

Figure S54. ^{1}H-NMR spectrum of **15a** in $CDCl_3$ (400 MHz).

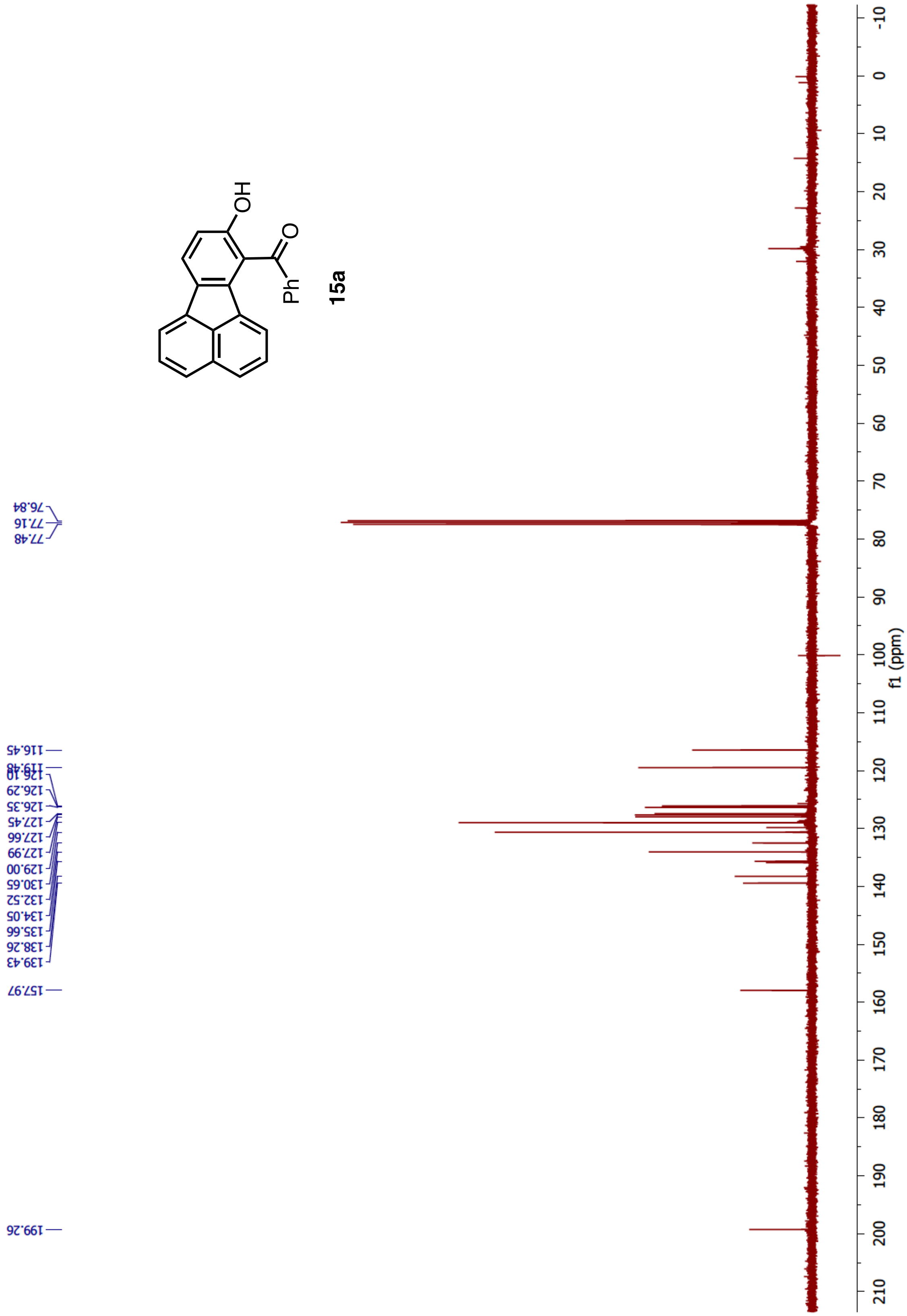

Figure S55. $^{13}C\{^{1}H\}$-NMR spectrum of **15a** in $CDCl_3$ (100 MHz).

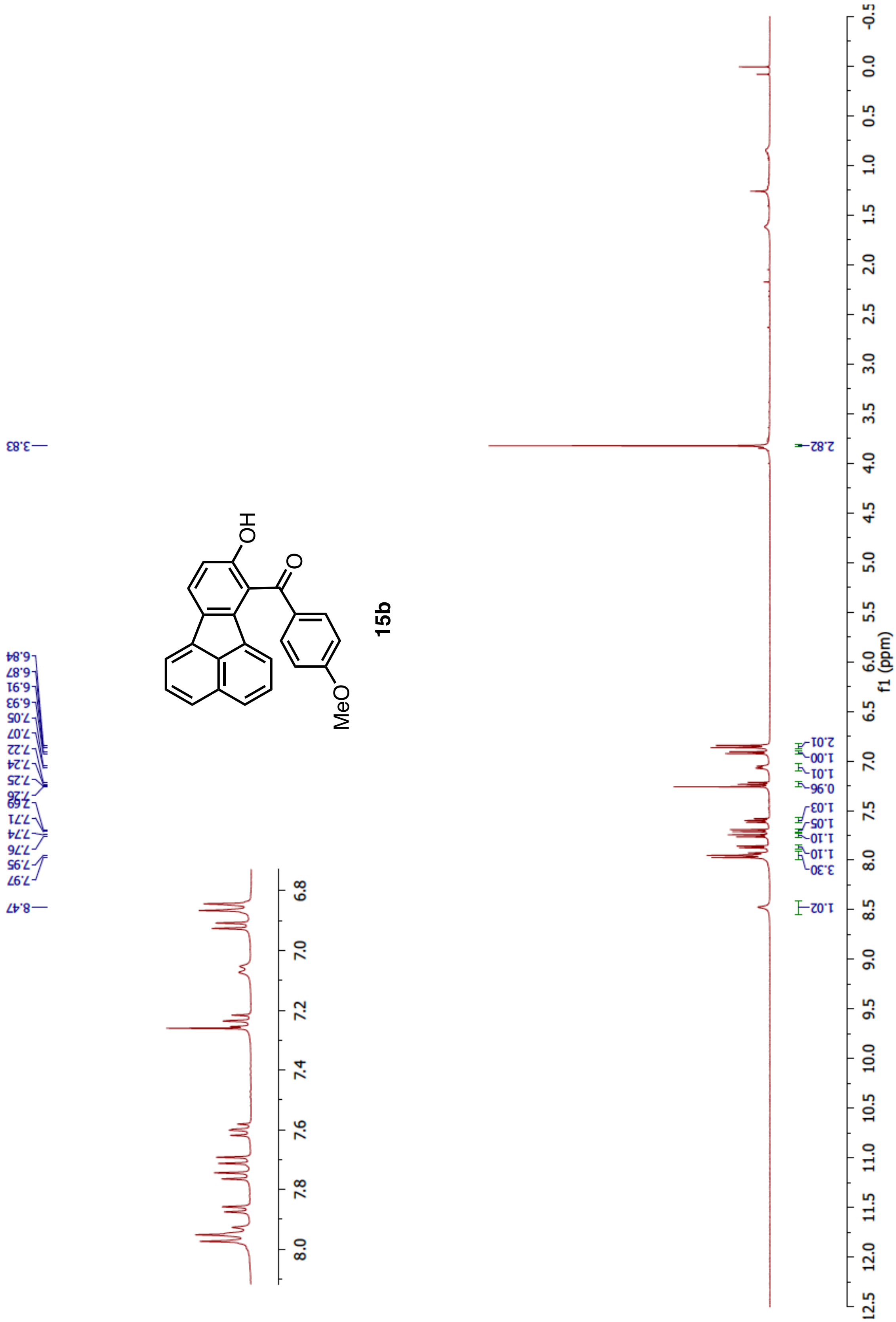

Figure S56. ^{1}H-NMR spectrum of **15b** in $CDCl_3$ (400 MHz).

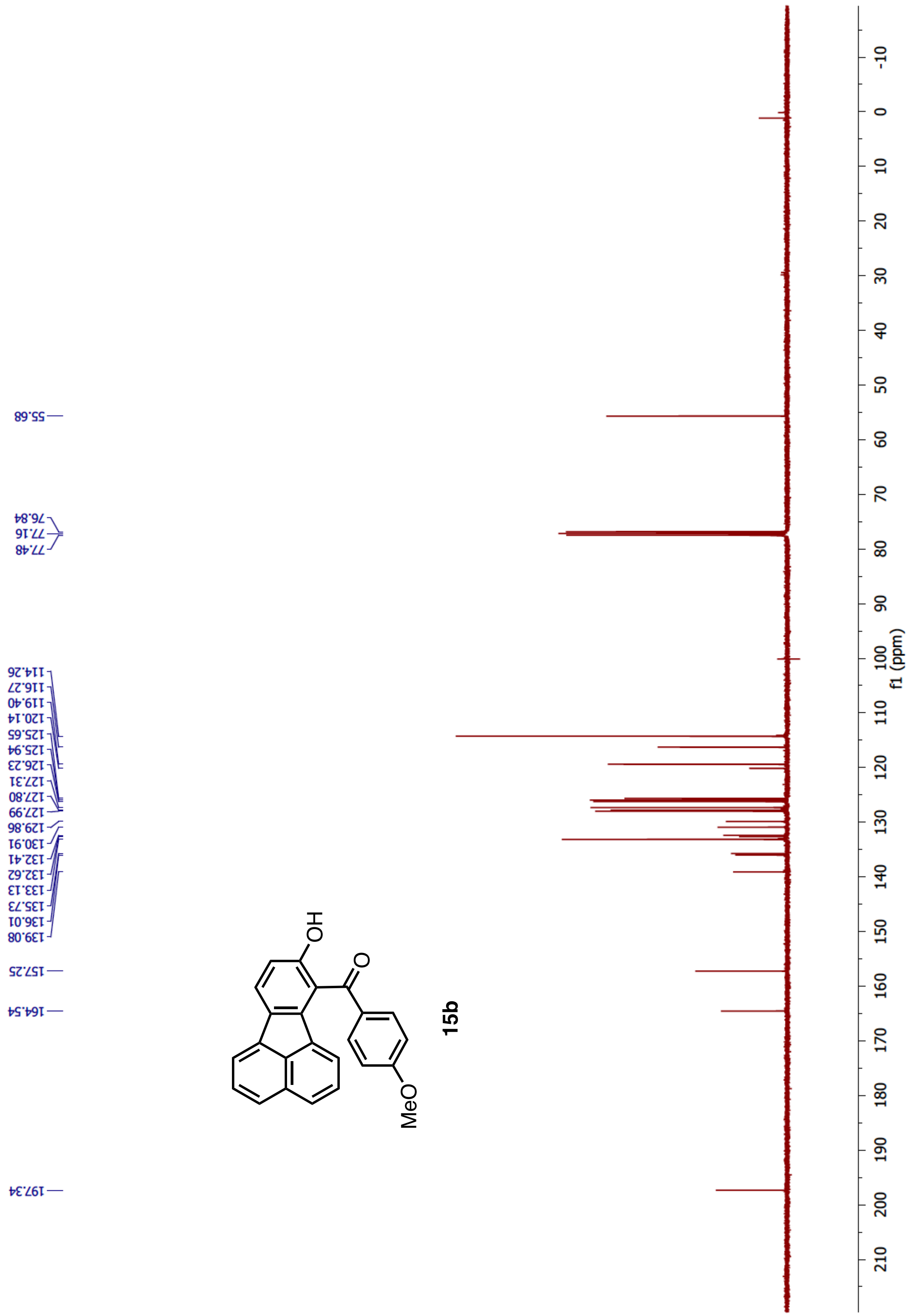

Figure S57. $^{13}C\{^{1}H\}$-NMR spectrum of **15b** in $CDCl_3$ (100 MHz).

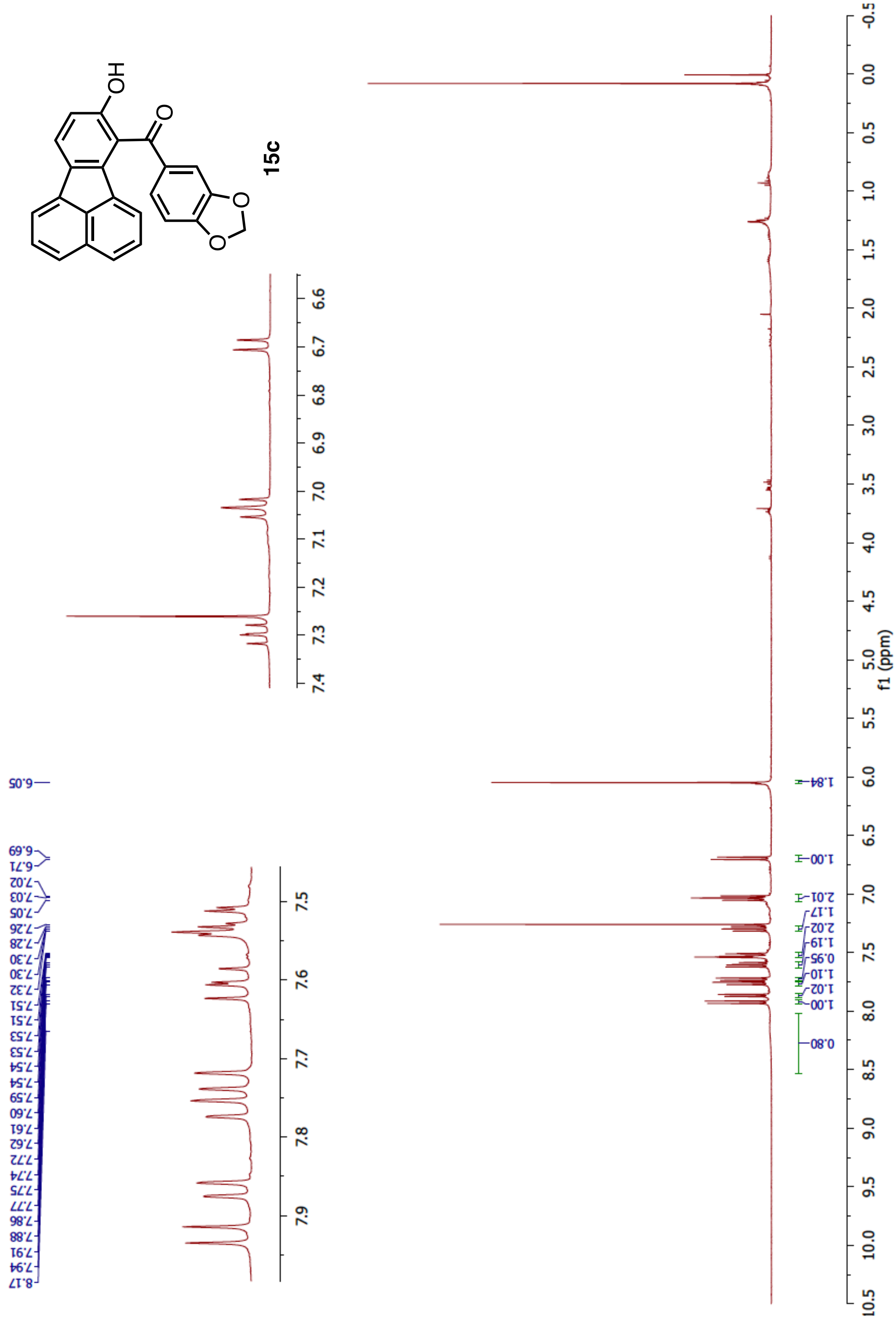

Figure S58. ^{1}H-NMR spectrum of **15c** in $CDCl_3$ (400 MHz).

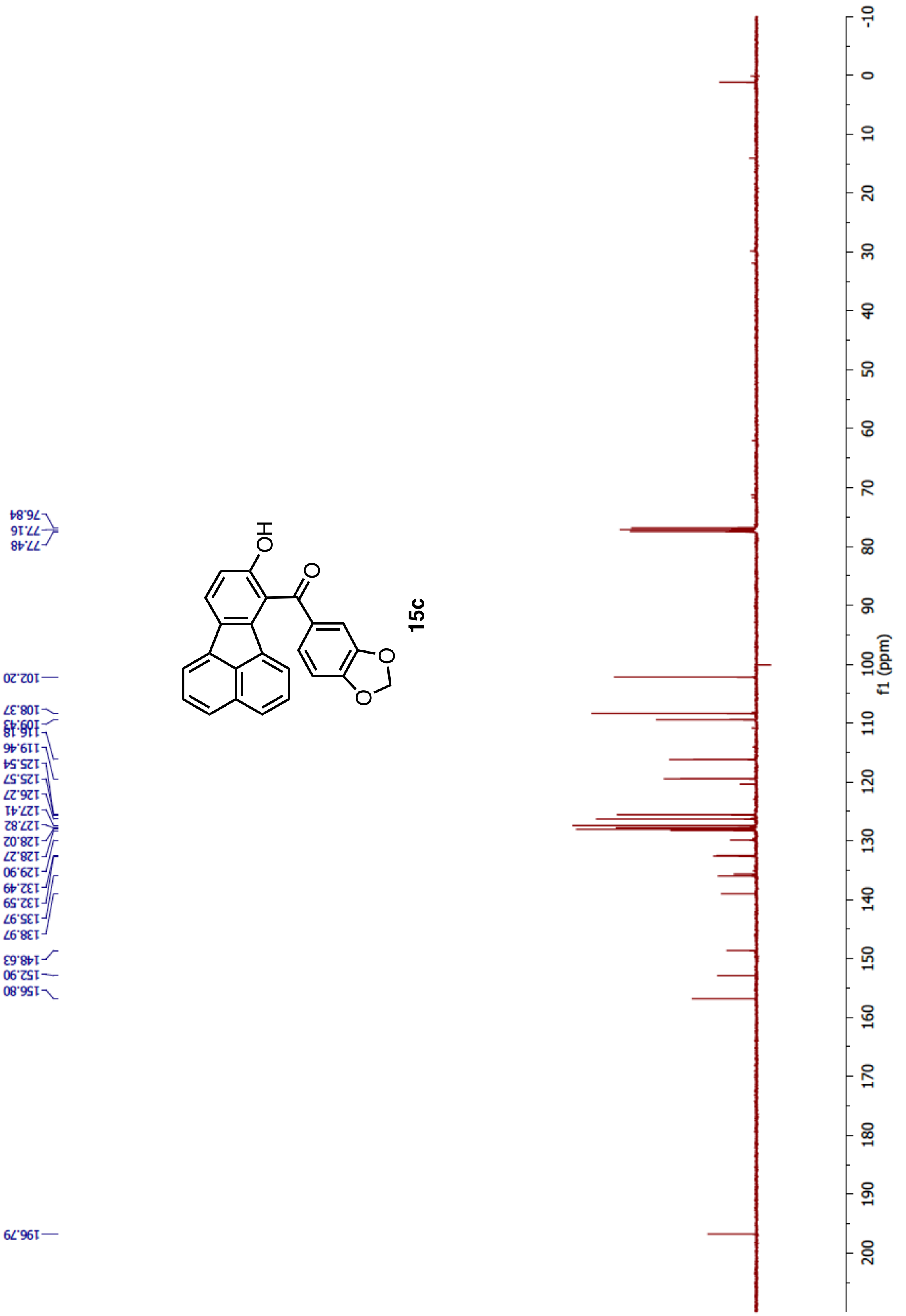

Figure S59. $^{13}C\{^{1}H\}$-NMR spectrum of **15c** in $CDCl_3$ (100 MHz).

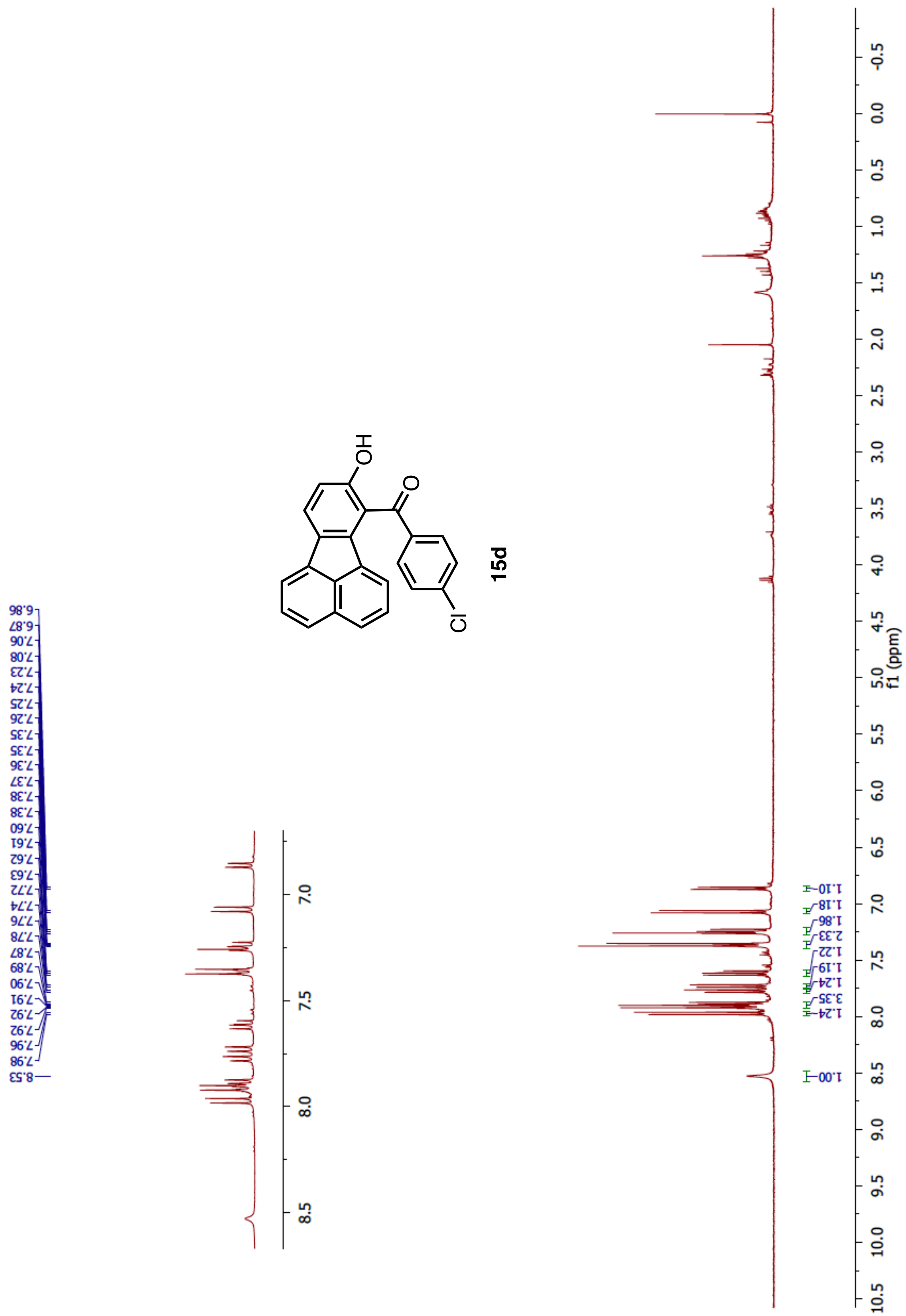

Figure S60. ^{1}H-NMR spectrum of **15d** in $CDCl_3$ (400 MHz).

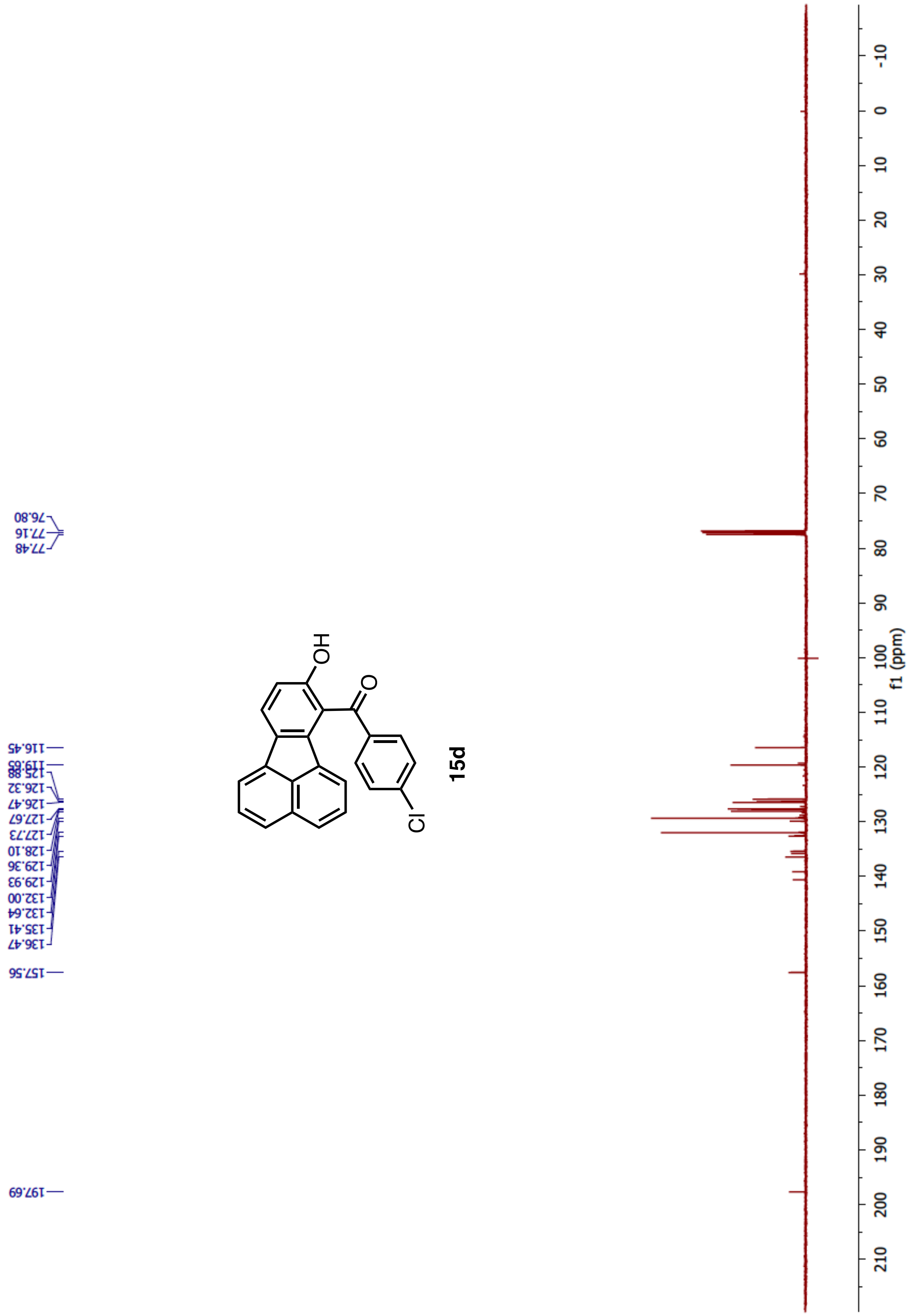

Figure S61. $^{13}C\{^{1}H\}$-NMR spectrum of **15d** in $CDCl_3$ (100 MHz).

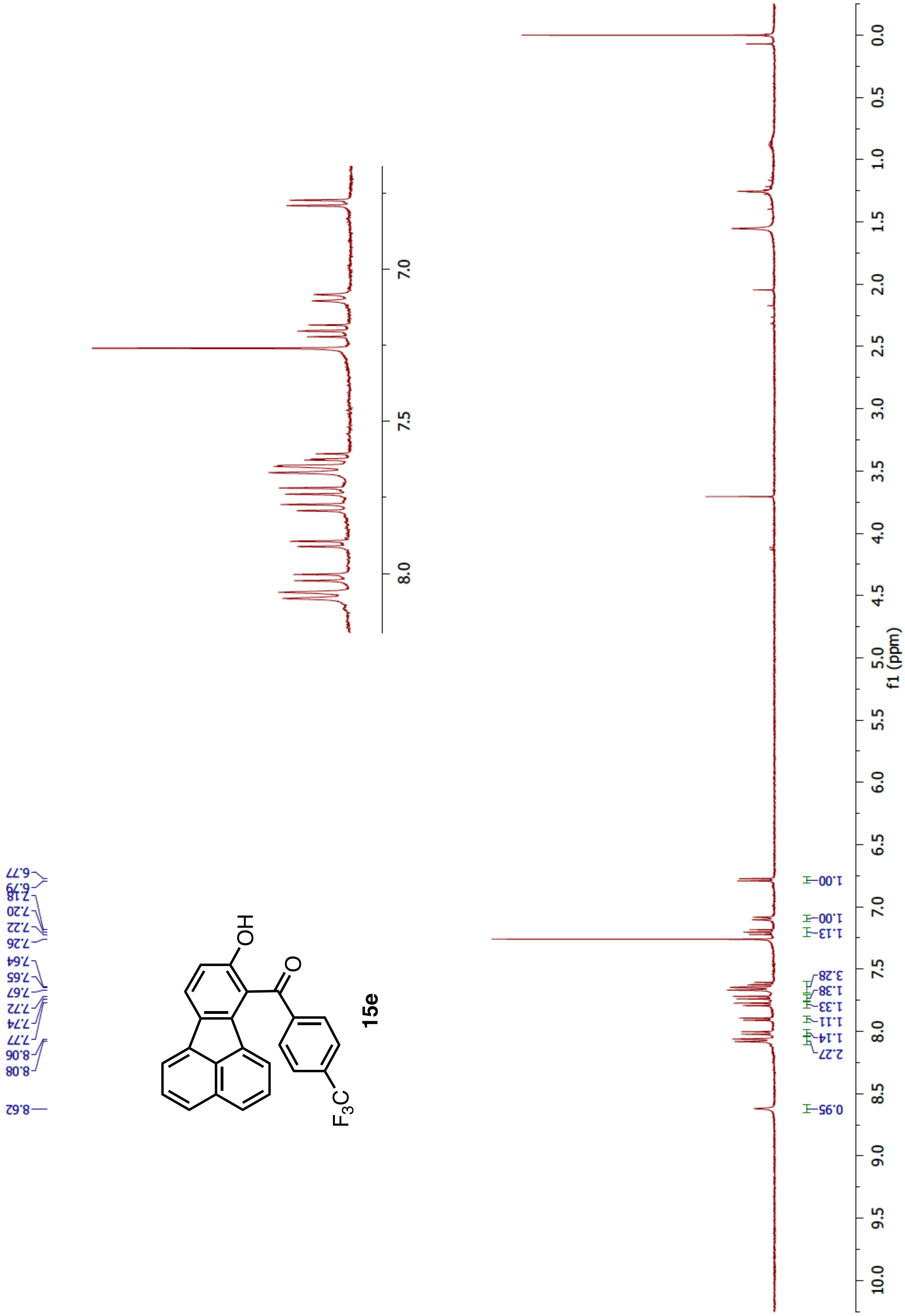

Figure S62. ^{1}H-NMR spectrum of **15e** in $CDCl_3$ (400 MHz).

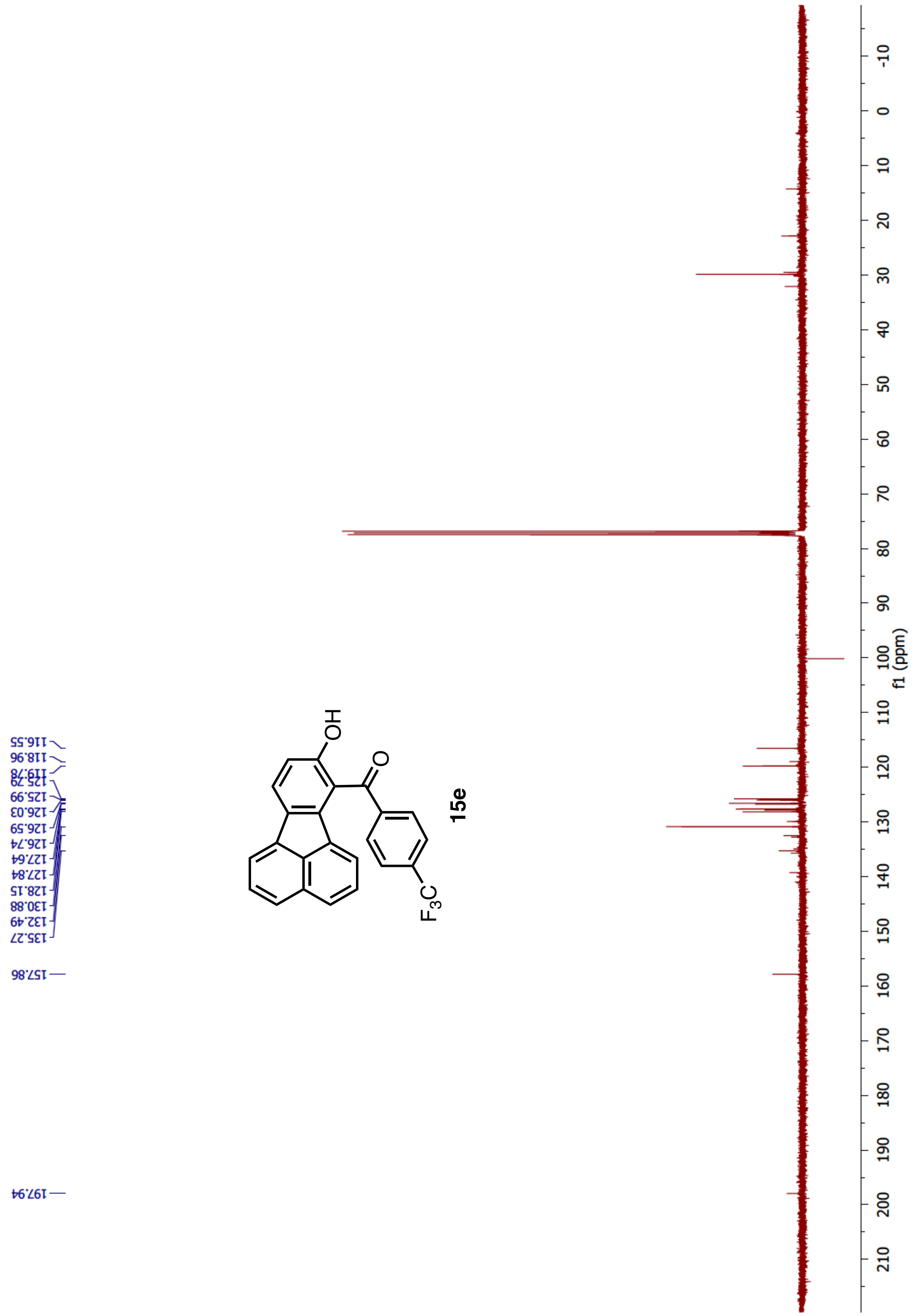

Figure S63. $^{13}C\{^{1}H\}$-NMR spectrum of **15e** in $CDCl_3$ (100 MHz).

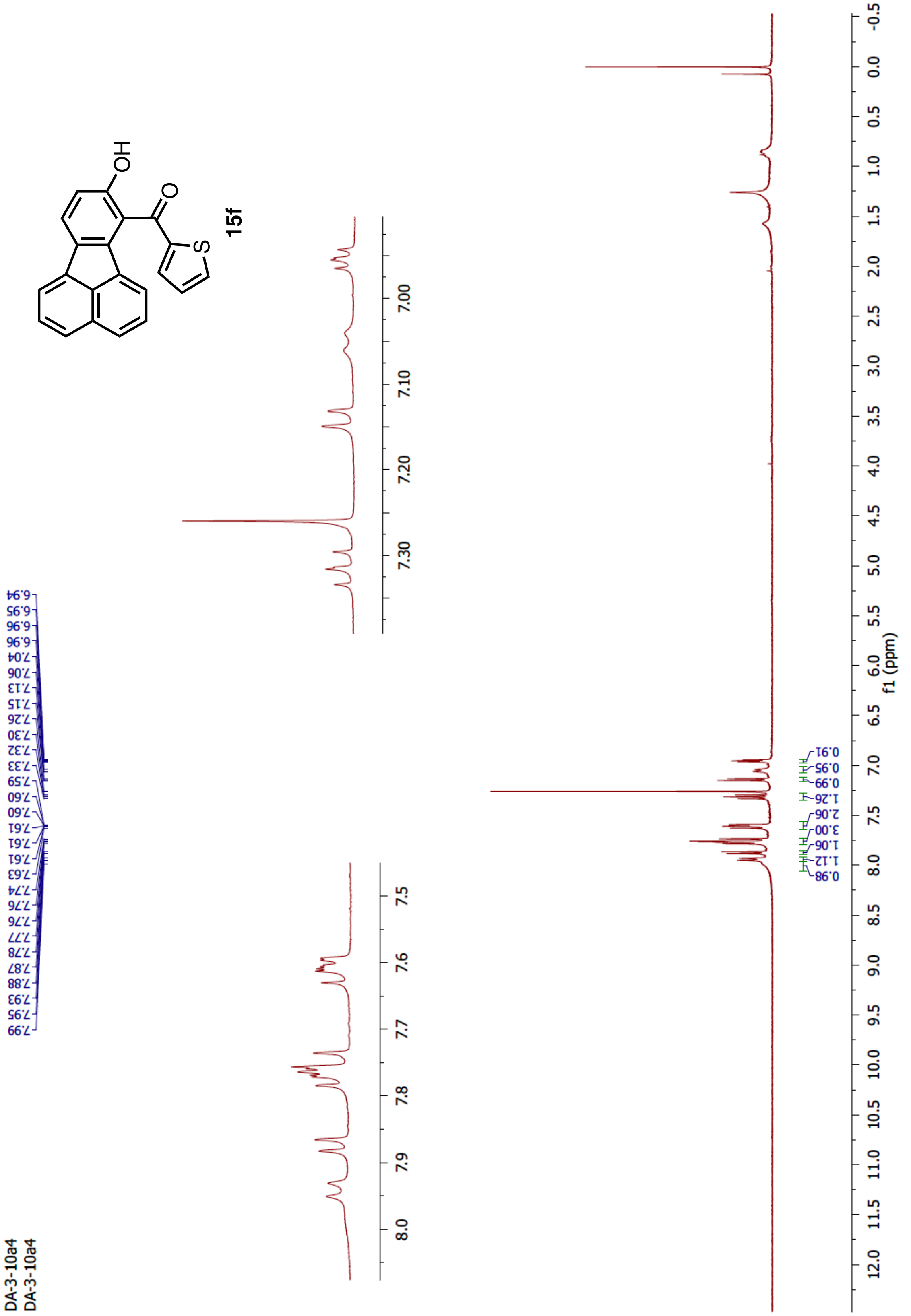

Figure S64. ^{1}H-NMR spectrum of **15f** in $CDCl_3$ (400 MHz).

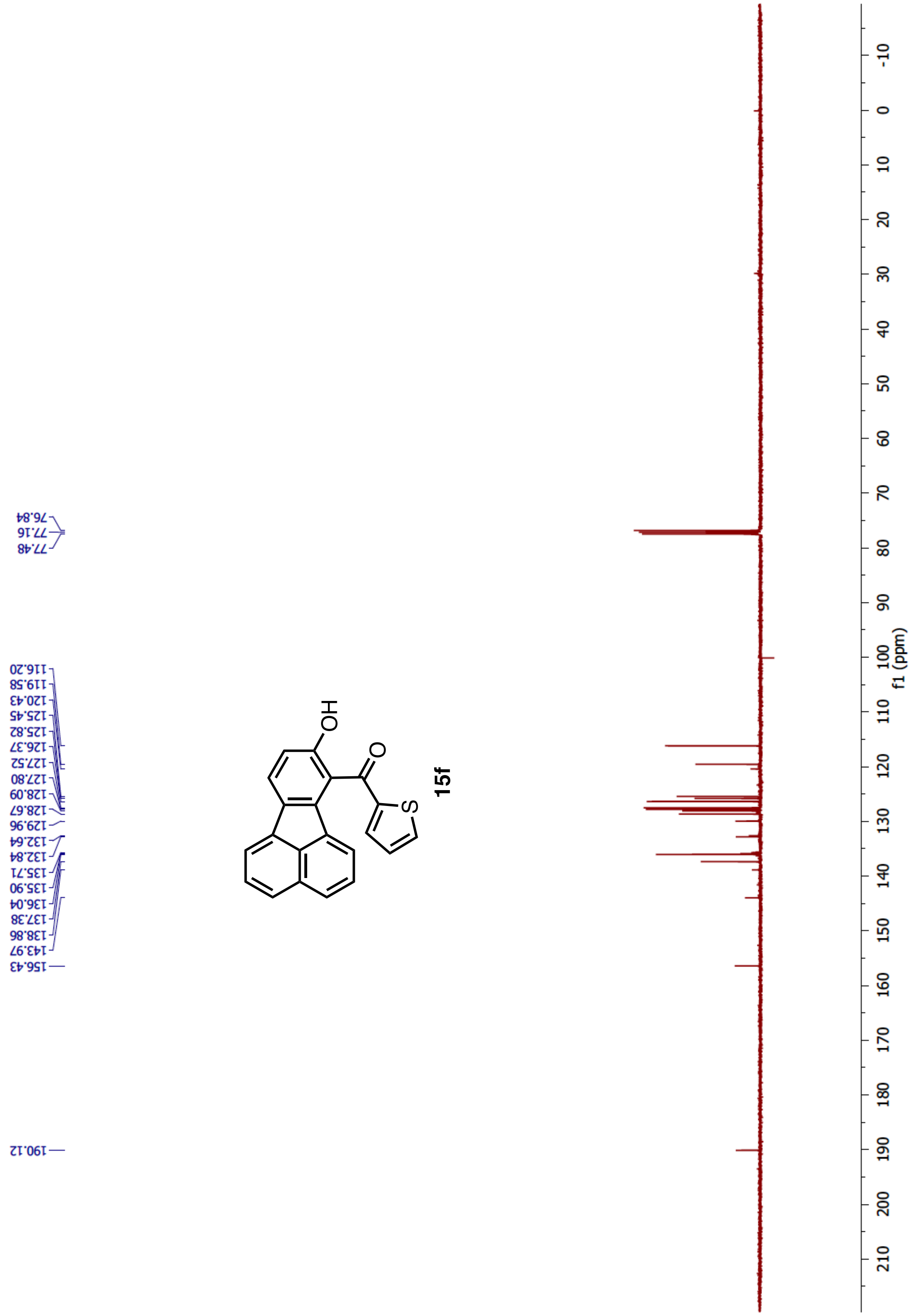

Figure S65. $^{13}C\{^{1}H\}$-NMR spectrum of **15f** in $CDCl_3$ (100 MHz).

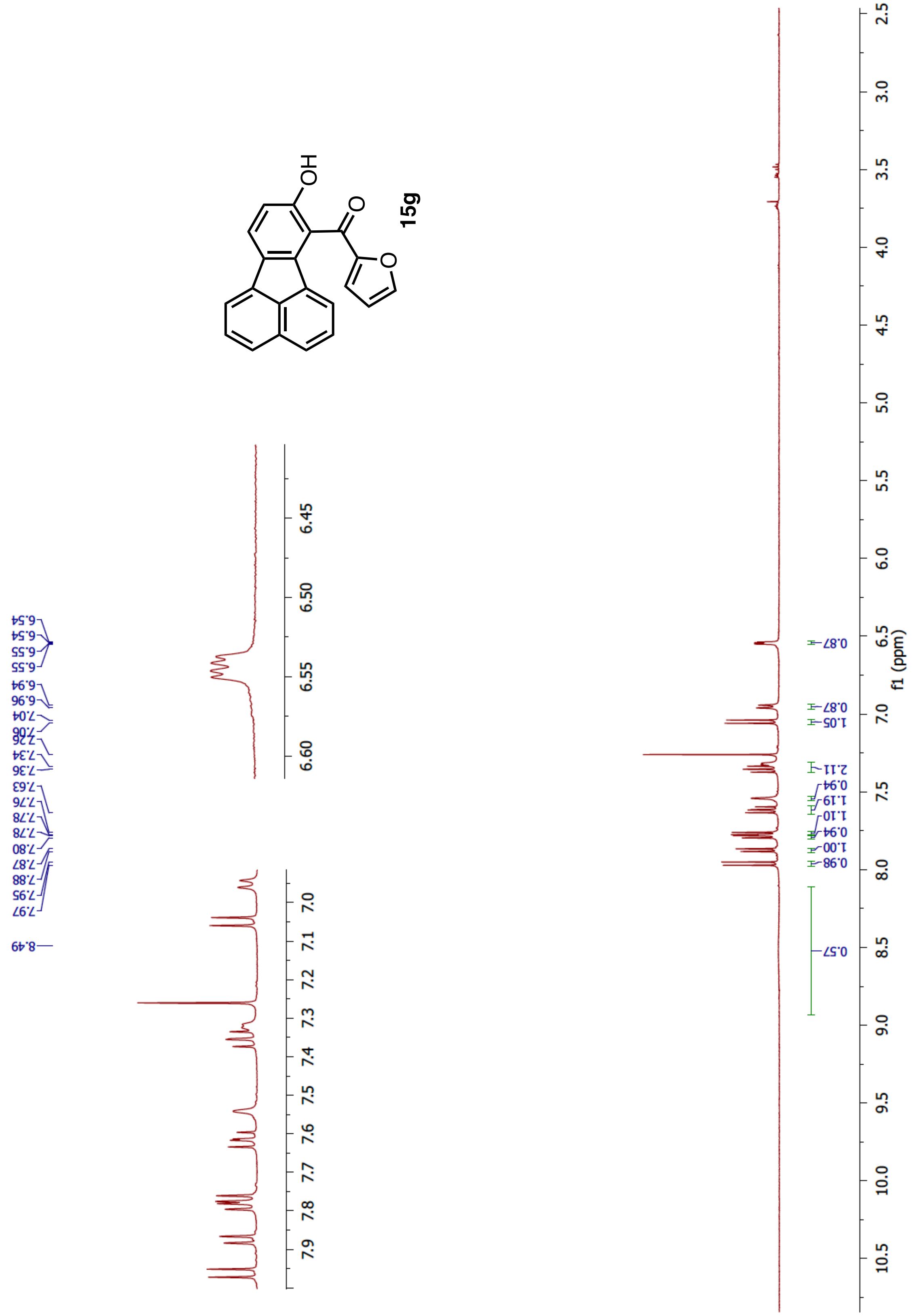

Figure S66. ^{1}H-NMR spectrum of **15g** in $CDCl_3$ (400 MHz).

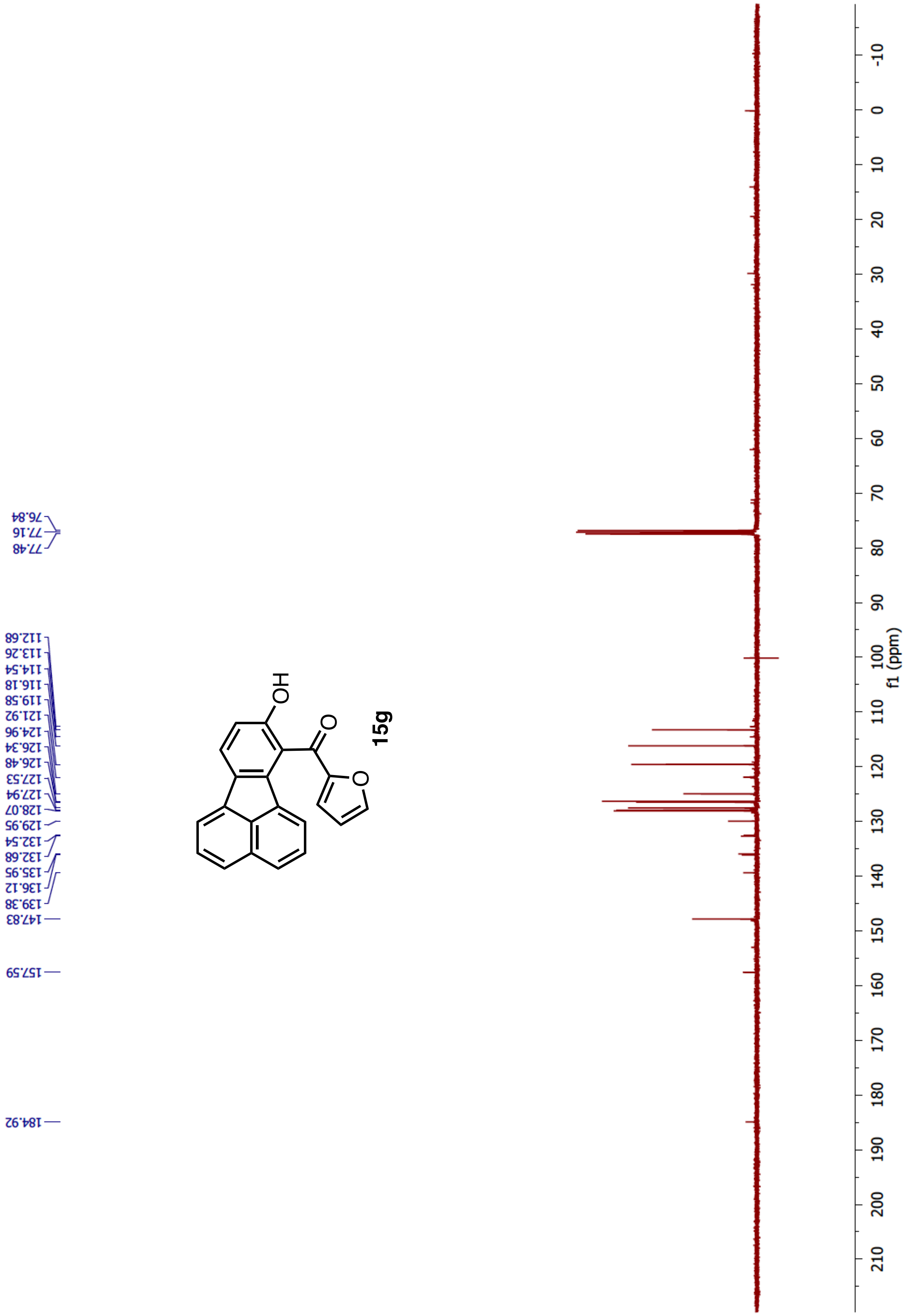

Figure S67. $^{13}C\{^{1}H\}$-NMR spectrum of **15g** in $CDCl_3$ (100 MHz).

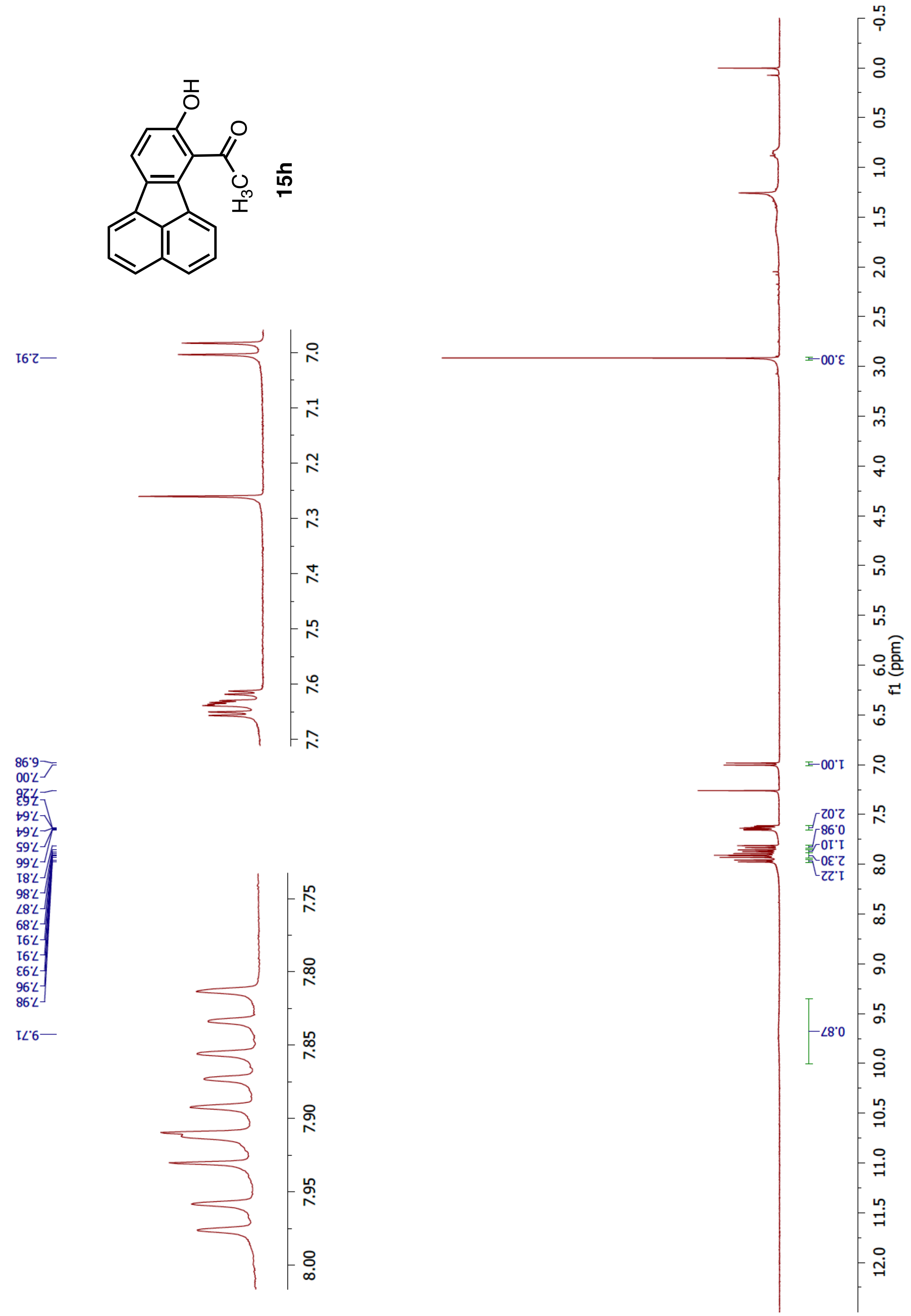

Figure S68. ^{1}H-NMR spectrum of **15h** in $CDCl_3$ (400 MHz).

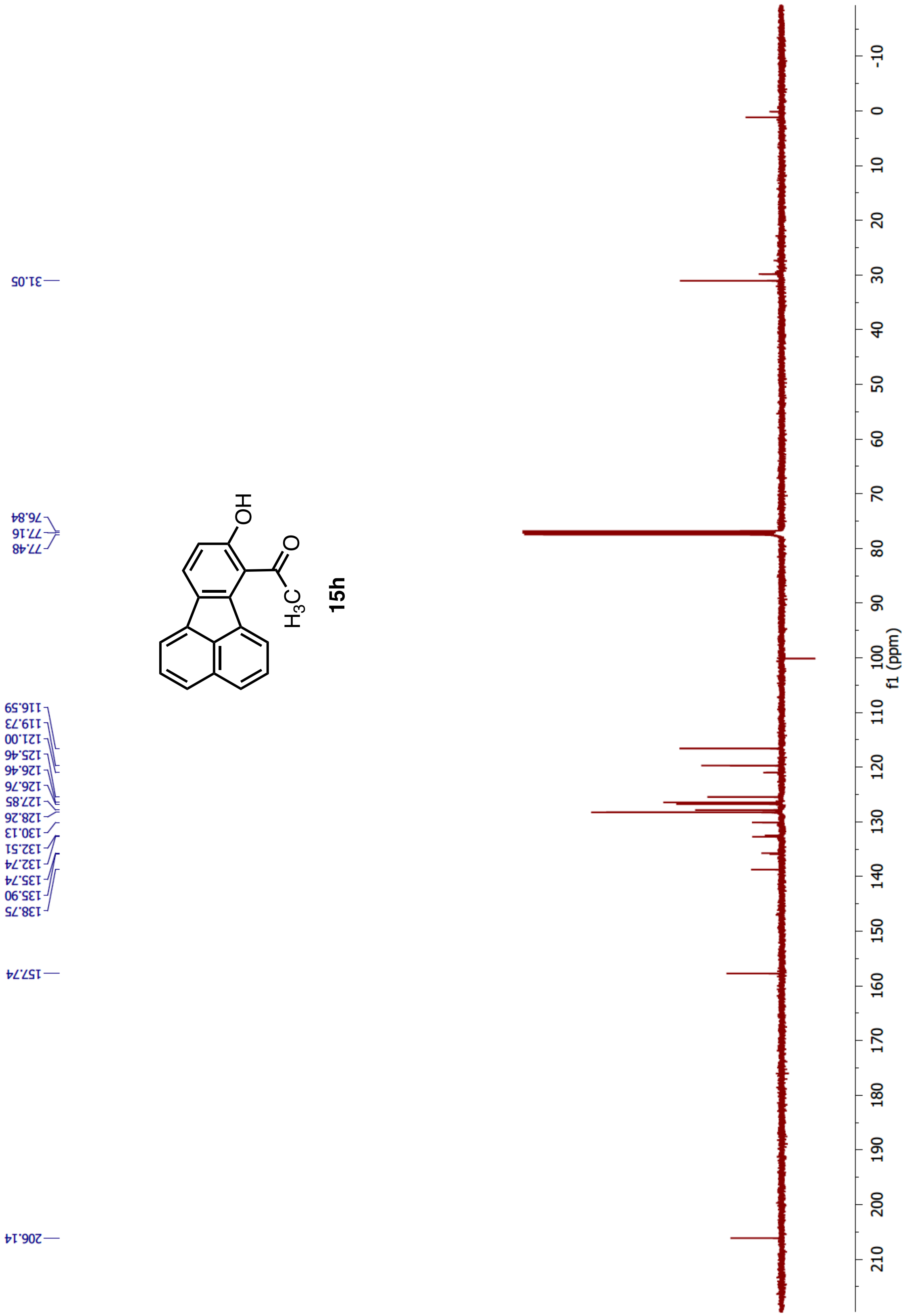

Figure S69. $^{13}C\{^{1}H\}$-NMR spectrum of **15h** in $CDCl_3$ (100 MHz).

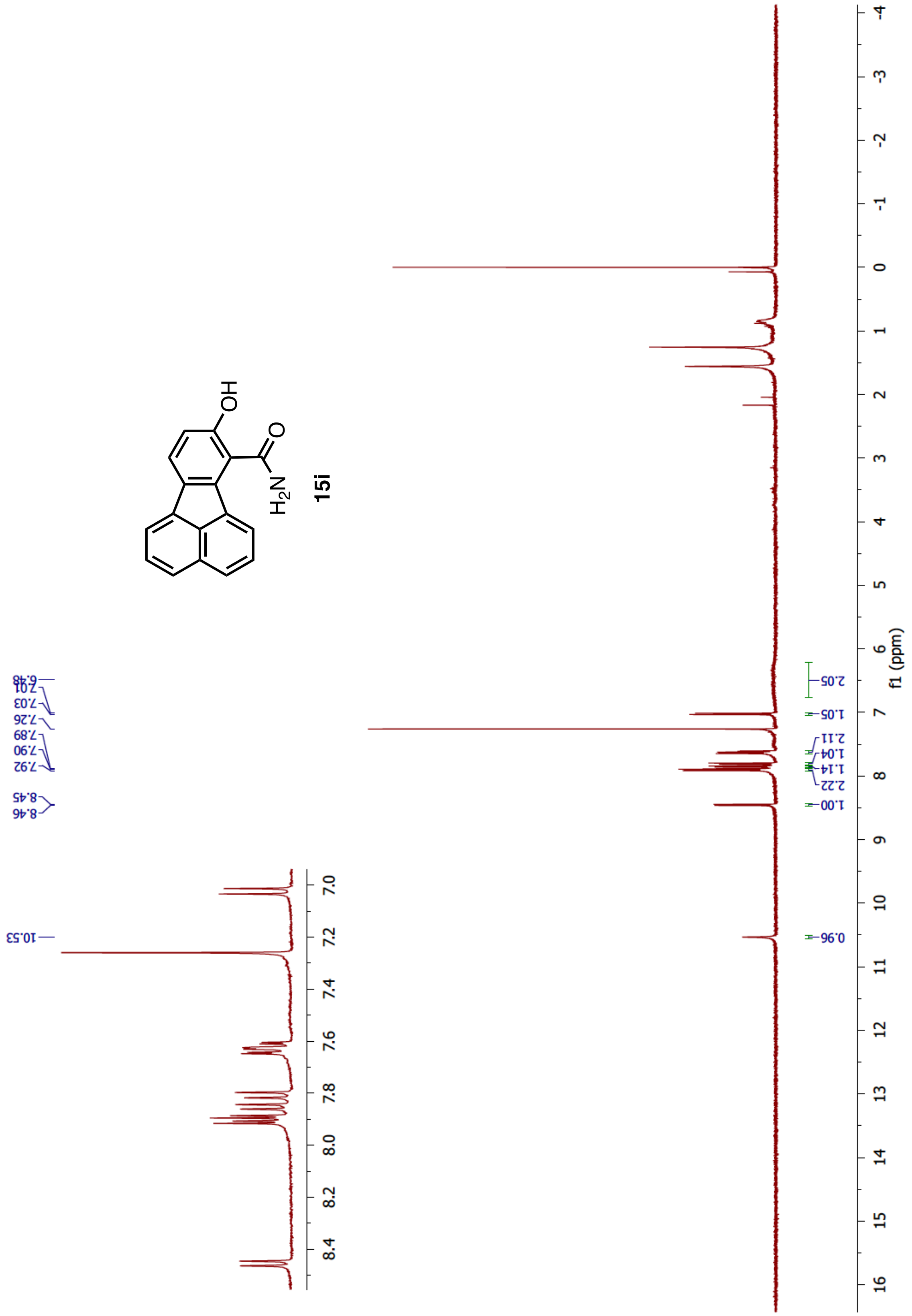

Figure S70. ^{1}H-NMR spectrum of **15i** in $CDCl_3$ (400 MHz).

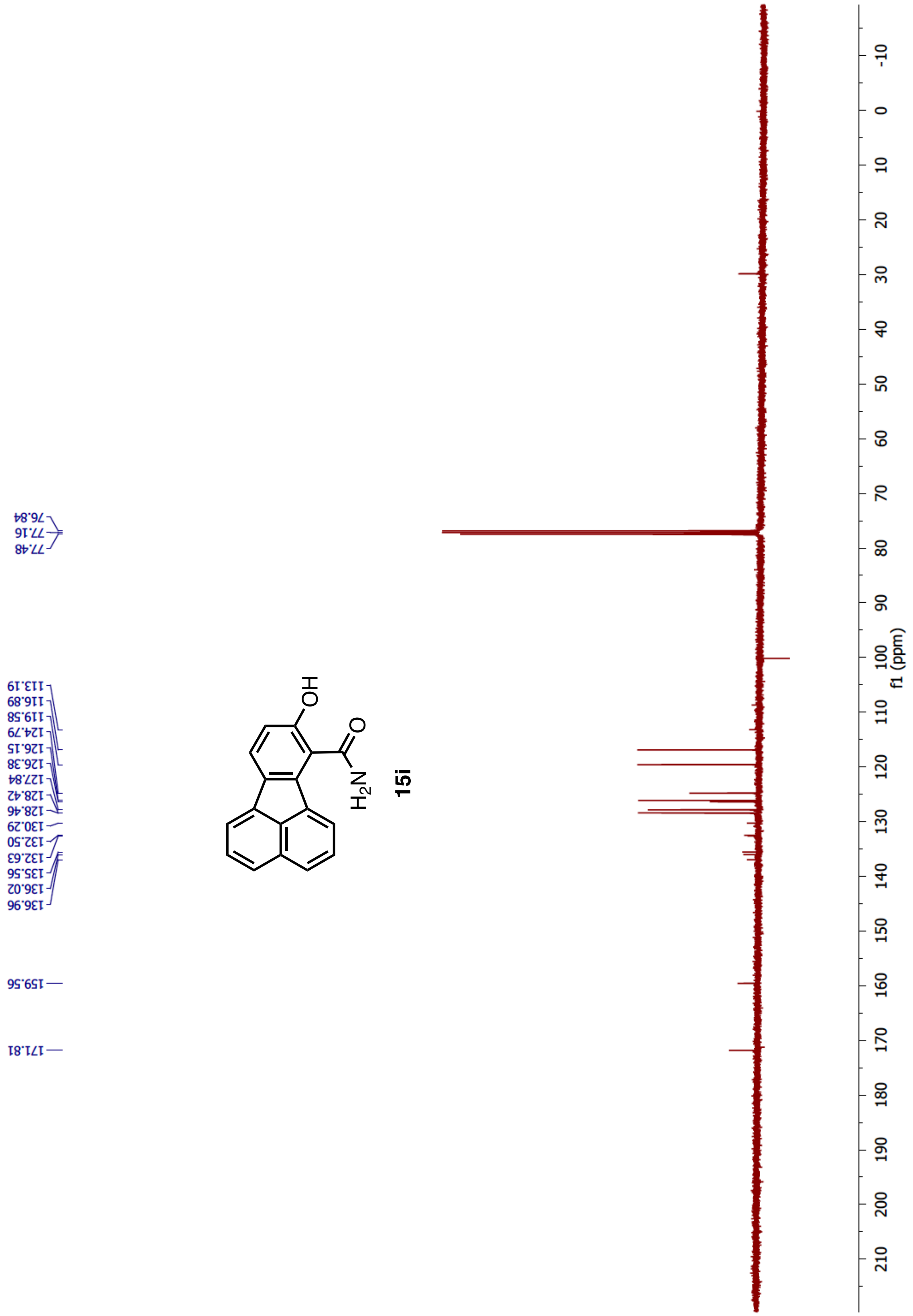

Figure S71. $^{13}C\{^{1}H\}$-NMR spectrum of **15i** in $CDCl_3$ (100 MHz).

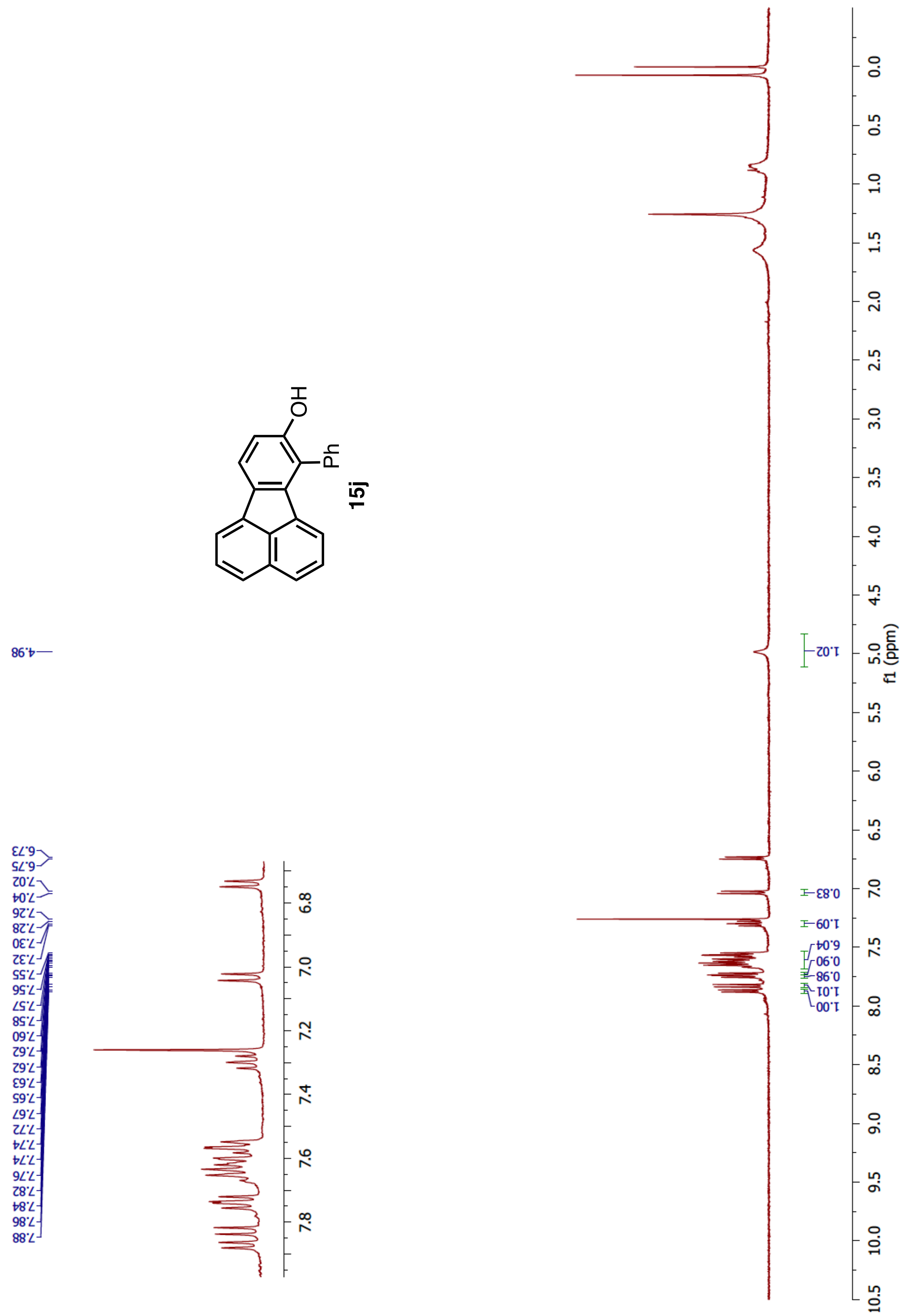

Figure S72. ^{1}H-NMR spectrum of **15j** in $CDCl_3$ (400 MHz).

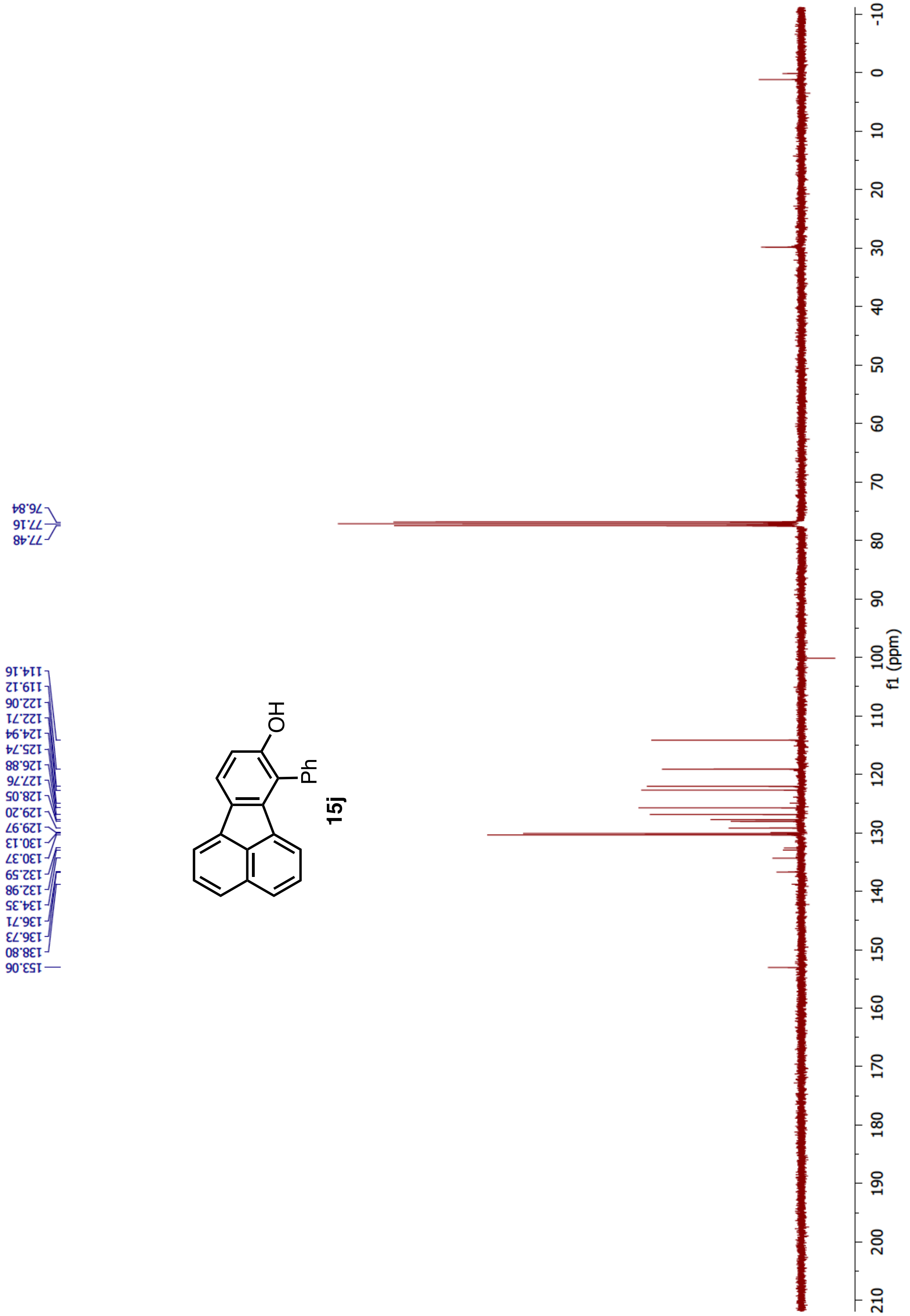

Figure S73. $^{13}C\{^{1}H\}$-NMR spectrum of **15j** in $CDCl_3$ (100 MHz).

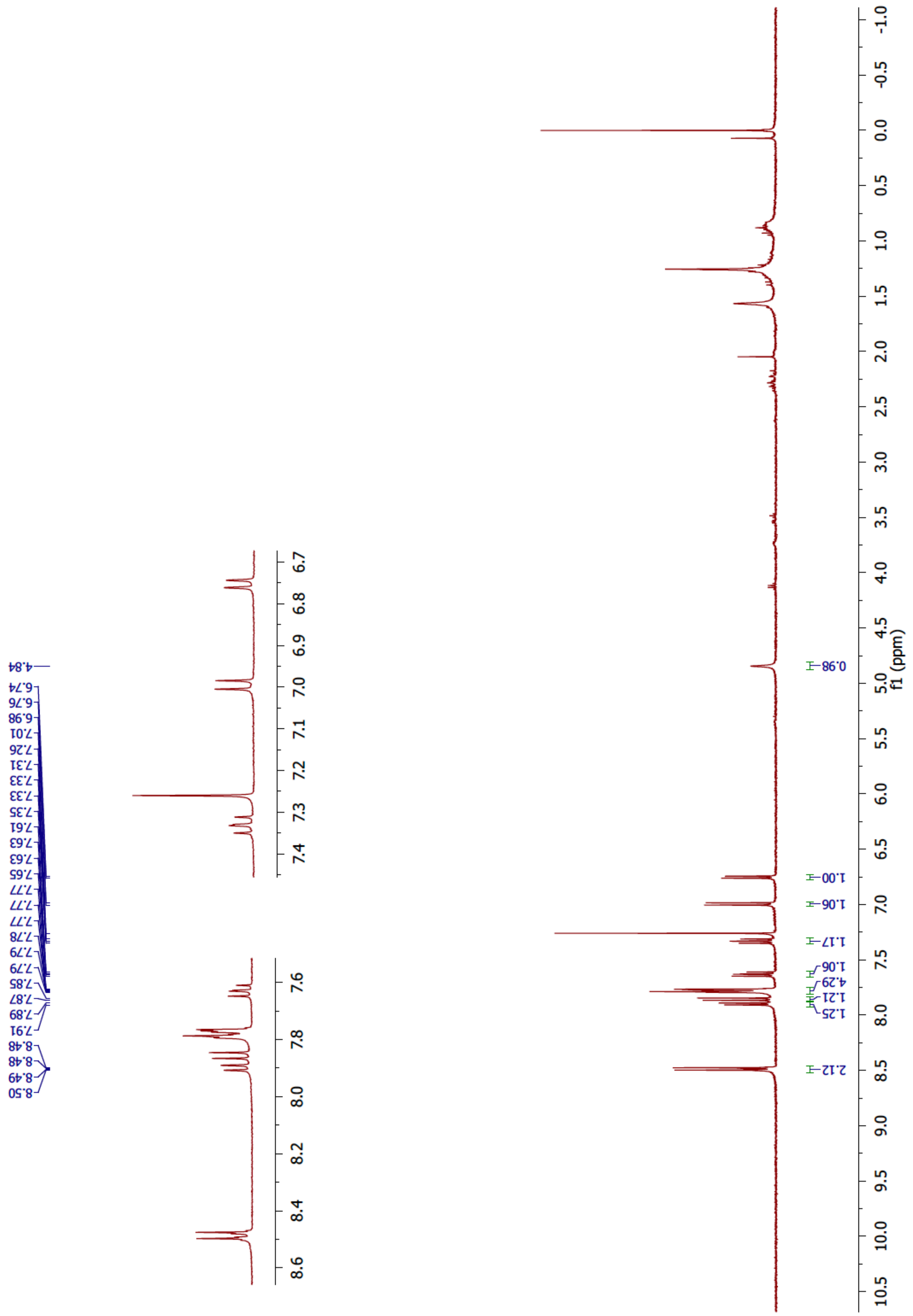

Figure S74. ^{1}H-NMR spectrum of **15k** in $CDCl_3$ (400 MHz).

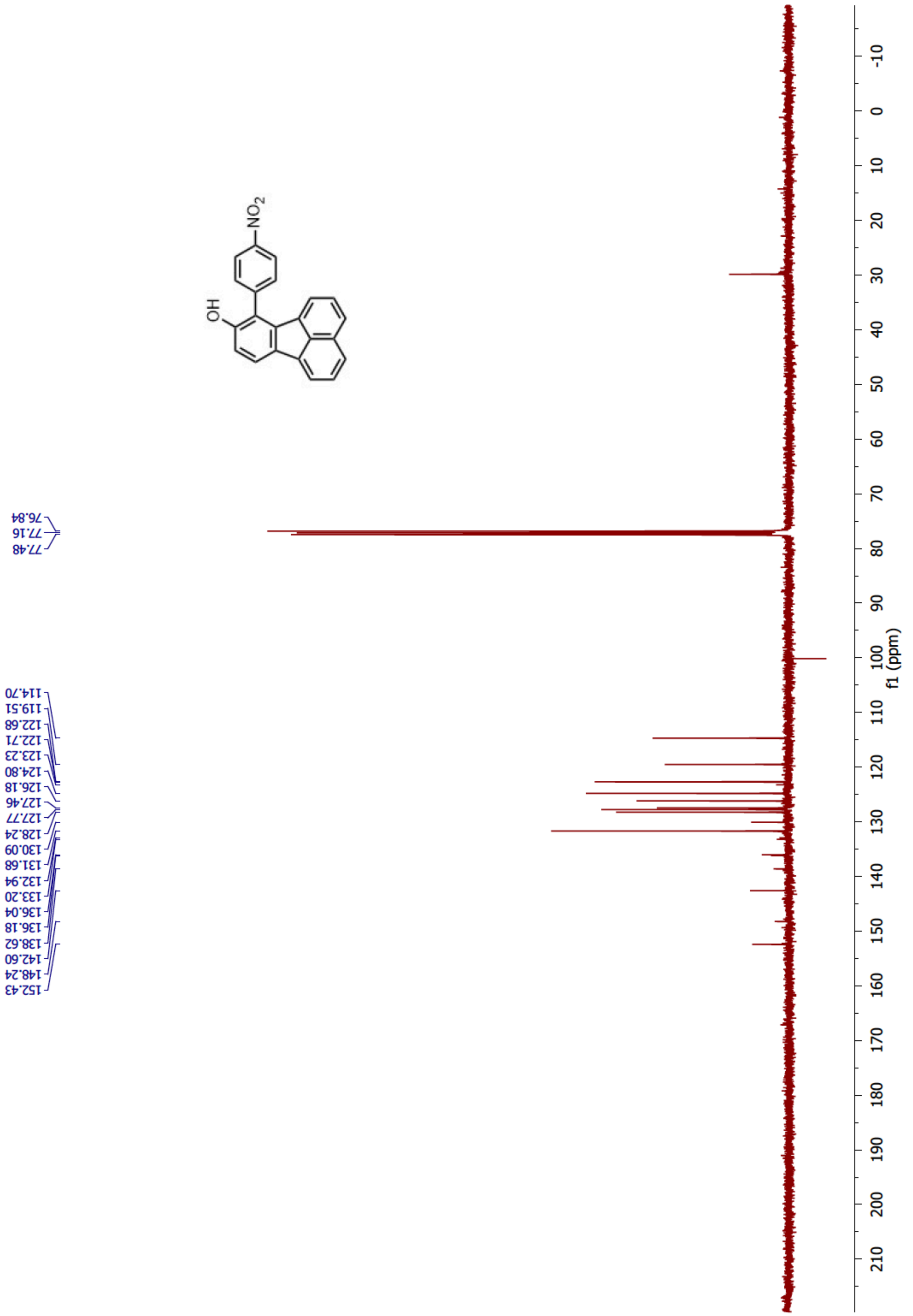

Figure S75. $^{13}C\{^{1}H\}$-NMR spectrum of **15k** in $CDCl_3$ (100 MHz).

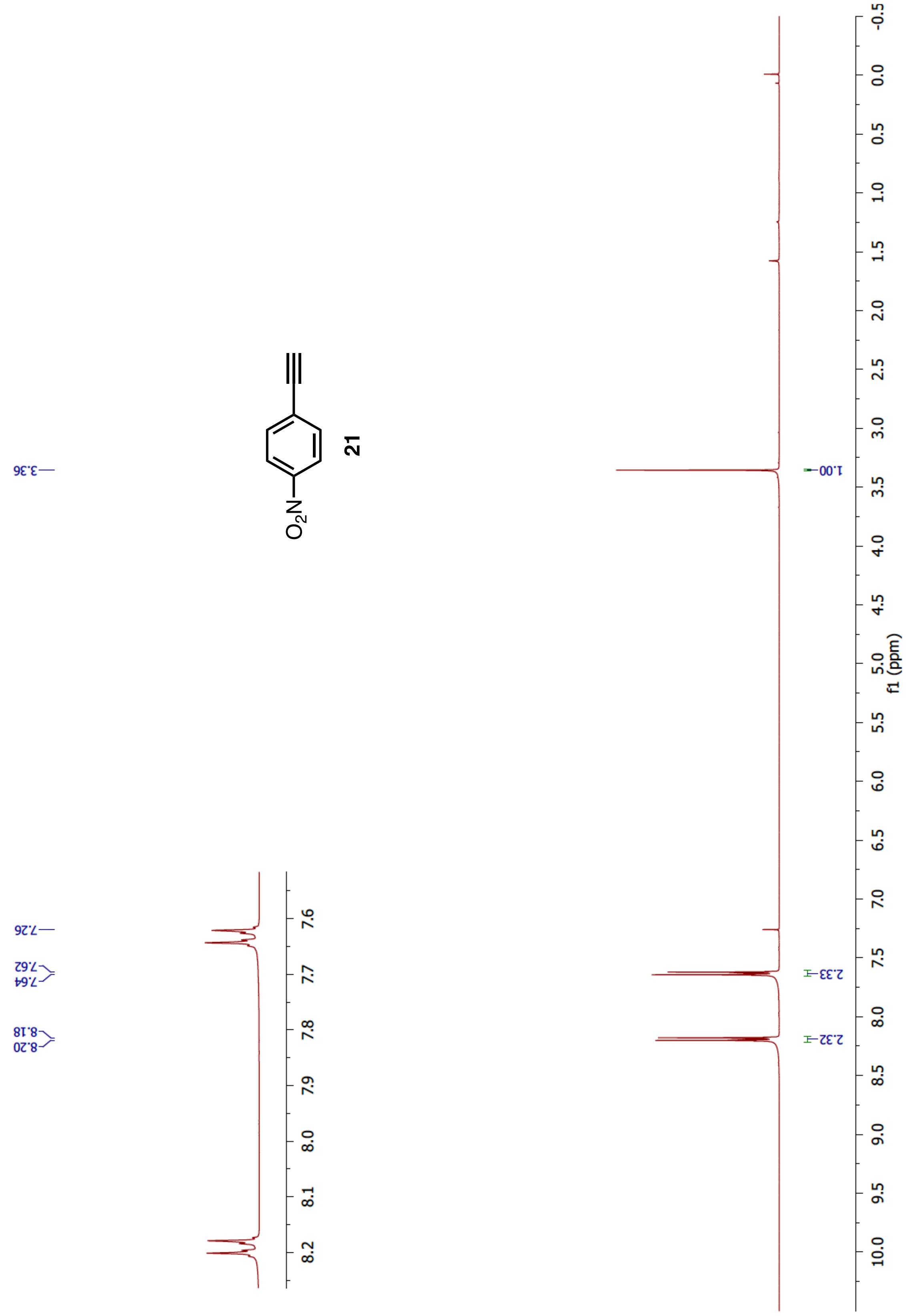

Figure S76. ^{1}H-NMR spectrum of **21** in $CDCl_3$ (400 MHz).

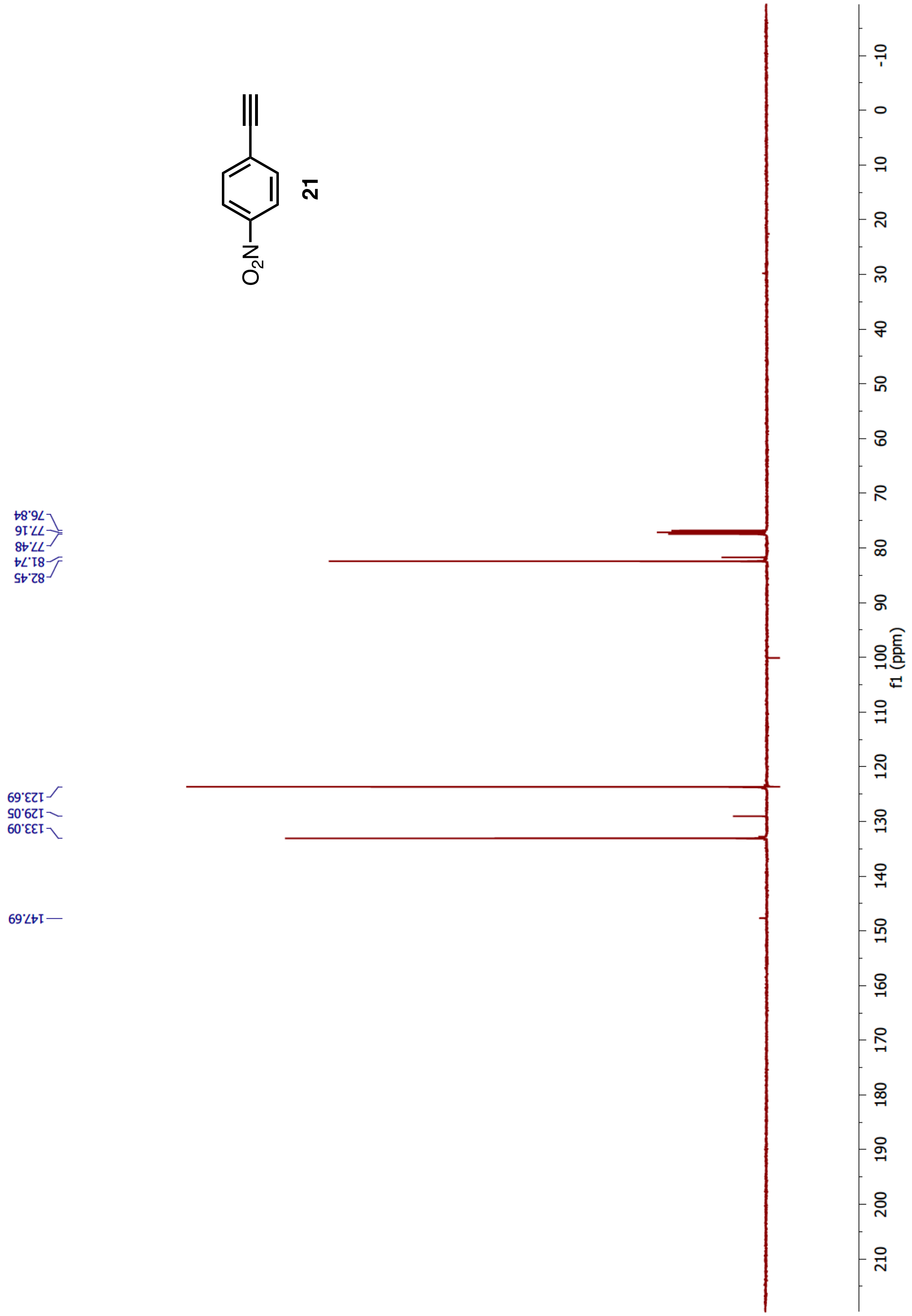

Figure S77. $^{13}C\{^{1}H\}$-NMR spectrum of **21** in $CDCl_3$ (100 MHz).
